# Lactylation of HMGB1 at K177 Drives Nuclear Export of TIAR to Promote Hypoxia‐Induced Stress Granule Formation

**DOI:** 10.1002/advs.202504896

**Published:** 2025-08-11

**Authors:** Chengyu Li, Zhaojun Liu, Linjie Zhu, Gang Wu, Chen Fu, Hongmin Li, Tong He, Ming Shen, Honglin Liu

**Affiliations:** ^1^ College of Animal Science and Technology Nanjing Agricultural University Weigang 1 Nanjing 210095 China

**Keywords:** HMGB1, hypoxic stress granules, lactylation, nuclear export, TIAR

## Abstract

HMGB1, one of the most abundant nuclear non‐histone proteins, also performs extracellular functions, and its nuclear export mechanisms have been extensively studied. Here, a novel mechanism of nuclear export for HMGB1 driven by lactylation is proposed. In addition, it is revealed that hypoxia‐induced lactylation of HMGB1 facilitates its nuclear export in a complex with TIAR, promoting stress granule (SG) formation in the cytosol. Mass spectrometry revealed 12 lysine residues in HMGB1 undergoing lactylation, with K172 and K177 being the most susceptible. Functional studies using lysine‐to‐arginine mutants (K→R) demonstrated that lactylation at K177 is crucial for HMGB1‐TIAR complex export, as K177R mutation completely blocked this export and subsequent SG formation. Notably, this lactylation‐mediated mechanism is specific to hypoxic stress, while other stressors, such as sodium arsenite exposure and heat shock, triggered TIAR nuclear export and SG assembly independently of HMGB1. These findings reveal a previously unrecognized role of HMGB1 lactylation in mediating nuclear export and SG formation under hypoxia.

## Graphical abstract

This study uncovers a novel mechanism in which p300‑catalyzed lactylation of HMGB1 triggers nuclear export of the HMGB1‐TIAR complex, driving TIAR‐dependent SGs formation in the cytoplasm. Mass spectrometry and mutagenesis reveals that K177 lactylation is essential for this export and subsequent SGs formation. The lactylation‐specific pathway operates uniquely under hypoxia, distinguishing it from arsenite‐ or heat shock–induced SG responses.

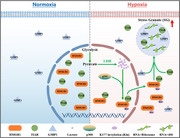

## 1 Introduction

Hypoxia is characterized by reduced oxygen availability in tissues or blood and is defined as a state in which oxygen demand surpasses oxygen supply. This condition is commonly associated with various pathological and physiological processes, including tumors, myocardial infarction, chronic inflammation, and stroke, in which it plays a critical role in disease progression and patient survival.^[^ ^1^ ^]^ As a form of type I cellular stress, hypoxia initially triggers adaptive or survival responses, enabling cells to mitigate the detrimental effects of oxygen deprivation. One such response is the formation of stress granules (SGs) in the cytoplasm.^[^ ^2^ ^]^ Despite ongoing studies, the mechanism by which hypoxia induces the formation of SGs remains unclear.

SGs are membrane‐free organelles formed in the cytoplasm of cells under stress conditions such as hypoxia, heatshock, arsenic exposure, viral infection, and inflammation, and they are one of the main stress‐management mechanisms of the cells.^[^ ^3^ ^]^ When cells are exposed to stress, TIAR, G3BP1, PABP1, or TIA‐1, proteins aggregate with RNA and sequester RNA, translation initiation factors, 40S ribosomal small subunits, and other proteins into SGs through liquid‐liquid phase separation (LLPS).^[^ ^4^ ^]^ When stress is removed, SGs disassemble, releasing RNA and functional proteins, which are then utilized by the cell to regulate behavior and restore homeostasis, thereby protecting cells from stress‐induced damage.^[^ ^5^ ^]^ SGs are known to recruit various functional proteins involved in critical biological processes, including apoptosis, proliferation, cell cycle regulation, metabolism, and immunity.^[^ ^4a^ ^]^ For instance, SGs have been shown to sequester TRAF2 during anti‐inflammatory responses, thereby inhibiting the NF‐κB signaling pathway.^[^ ^6^ ^]^ Under type I stress, SGs prevent MTK1‐mediated apoptosis by isolating RACK1.^[^ ^2^ ^]^ Furthermore, SGs are involved in the genesis and development of several diseases, including cancer and autoimmune and neurodegenerative diseases.^[^ ^3^ ^]^ The clinical mechanism targeting the formation of SGs to inhibit the development and progression of hypoxic tumors has become an important therapeutic approach.^[^ ^7^ ^]^ It is therefore crucial to investigate the molecular mechanisms governing hypoxia‐induced SG formation.

T‐cell intracellular antigen‐related protein (TIAR) is a multifunctional RNA‐binding protein associated with RNA polymerase II, DNA, and RNA that plays critical roles in cytoskeletal organization, mitochondrial function, cell proliferation, and the cell cycle through its regulation of RNA splicing, translation, and stability.^[^ ^8^ ^]^ Upon exposure to certain stresses, eIF2α is phosphorylated, which blocks the initiation of translation by inhibiting the formation of the ternary complex eIF2‐GTP‐tRNAi^Met^. As a consequence, TIAR binds to adenine‐ and uridine‐rich elements in the 3′ untranslated region (UTR) of target mRNAs, promoting the assembly of SGs.^[^ ^9^ ^]^ TIAR is one of the most well‐characterized proteins involved in SG formation.^[^ ^10^ ^]^ Due to this abundance, cytoplasmic TIAR foci, similar to those of G3BP1, are widely used as reliable markers for SG formation. In mammalian somatic cells, TIAR is primarily localized in the nucleus but can shuttle between the nucleus and cytoplasm. Upon exposure to stress, TIAR accumulates in the cytoplasm, facilitating SG assembly.^[^ ^11^ ^]^ However, the molecular mechanisms governing the nuclear export of TIAR, which serves as a foundation for SG assembly under stress, remain poorly understood.

High‐mobility group box 1 (HMGB1) is a highly conserved and ubiquitously expressed nuclear protein in eukaryotic cells known for its abundance in the nucleus, where it binds to DNA, facilitating chromatin organization, transcriptional regulation, and interactions with other proteins such as p53.^[^ ^12^ ^]^ In 1999, Wang et al. detected high levels of HMGB1 protein in serum from endotoxin‐treated mice and identified HMGB1 as a key cytokine in endotoxin‐mediated mouse death.^[^ ^13^ ^]^ Their study demonstrated for the first time that HMGB1 protein, traditionally thought to be located in the nucleus, is a major endogenous signal molecule and has since sparked much research into whether HMGB1 can be released from the nucleus to play a role in the cytoplasm or even outside the cell. Subsequent studies have demonstrated that cellular stress and death, particularly under hypoxic conditions, can trigger HMGB1 export from the nucleus. For instance, hypoxia has been shown to drive HMGB1 release from liver cell nuclei,^[^ ^14^ ^]^ whereas ischemia‐reperfusion promotes its release from brain tissue in ovine fetuses.^[^ ^15^ ^]^ In addition, hypoxia has been shown to induce the death of certain tumor cells and trigger the release of HMGB1 from the nucleus into the extracellular space, where it acts as a signal to promote tumor angiogenesis.^[^ ^16^ ^]^ However, the potential molecular mechanisms underlying hypoxia‐induced HMGB1 nuclear export have not been elucidated.

Lactylation is a post‐translational modification mediated by lactate, a key metabolite of hypoxic glycolysis. During this modification, lactyl‐coenzyme A from L‐lactate neutralizes the positive charge of the lysine side chain via a p300‐mediated enzymatic reaction, resulting in lysine lactylation, which can be removed by delactylases such as SIRT1‐3 and HDAC1‐3.^[^ ^17^ ^]^ Initially identified on histone lysines, lactylation is known to influence chromatin structure, modulate DNA accessibility, and regulate gene expression.^[^ ^18^ ^]^ However, lactylation is not limited to histones; it has also been identified on non‐histone proteins and has been implicated in various biological processes, including neuronal activity, immunosuppression, and exosome release.^[^ ^12^, ^19^ ^]^ A recent study showed that adding lactate to macrophages promotes cytoplasmic accumulation and exosomal secretion of lactylated HMGB1,^[^ ^20^ ^]^ indicating a strong correlation between HMGB1 lactylation and its nuclear export, although the precise mechanisms underlying this correlation remain to be elucidated.

Here, we identified a novel function of HMGB1 in promoting SG formation under hypoxic stress, with lactylation emerging as a critical regulatory mechanism in this process.

## 2 Results

### 2.1 Hypoxia‐Induced SG Formation is Accompanied by HMGB1 Nuclear Export

**Figure 1:**
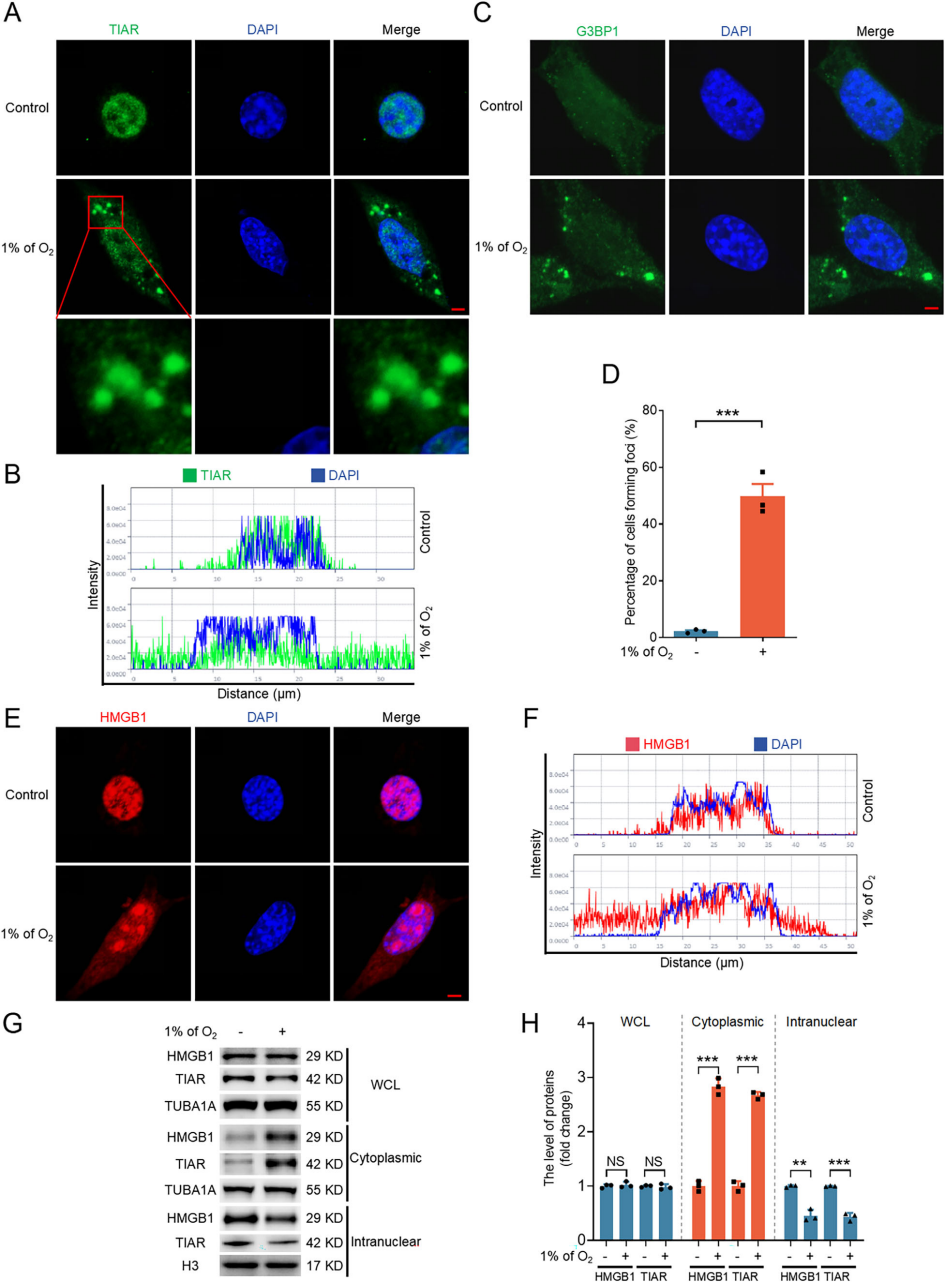
Hypoxia promotes the formation of SGs and the nuclear export of HMGB1. A–G, NIH/3T3 cells were cultured under normoxia (21% of O_2_) or hypoxia (1% of O_2_) conditions for 2 h and then collected to observe the subcellular localization of TIAR A), G3BP1 C), or HMGB1 E) by immunofluorescence assay. The proportion of cells that contain foci formed by TIAR was counted by laser confocal microscopy D). The fluorescence intensity curve shows the distribution of TIAR (green) B) or HMGB1 (red) F) and DAPI (blue) along the cells. Scale bar = 5 µm. Cells were obtained for western blotting analysis of the protein level of HMGB1 or the protein level of TIAR after nuclear and cytoplasmic extraction G) and quantified H). Data are presented as mean ± s.e.m. (n = 3). *P < 0.05, **P < 0.01, ***P < 0.001; NS, not significant (*P* > 0.05).

Hypoxia, as a type I stress, has been reported to promote SG formation, with multiple studies demonstrating that 1% O_2_ can induce SG assembly.^[^ ^21^ ^]^ TIAR and G3BP1 are essential components required for SG assembly.^[^ ^3^ ^]^ To investigate whether hypoxia facilitates SG formation, NIH/3T3 cells were cultured under hypoxic conditions (1% O_2_) for 2 h, followed by immunofluorescence analysis. The results revealed the aggregation of TIAR (**Figure** **1**A,B) and G3BP1 (Figure 1C) into distinct foci, with ≈50% of the cells exhibiting SG formation (Figure 1D). To further confirm that hypoxia induces SG formation, we examined the phosphorylation status of eIF2α at Ser51 (p‐eIF2α), a key marker of the integrated stress response (ISR). Western blot analysis showed a significant upregulation of p‐eIF2α as early as 0.5 h after hypoxic treatment (Figure S1A,B, Supporting Information). Given that SG formation is a reversible process, we returned hypoxia‐treated cells to normoxic conditions and observed a marked reduction in TIAR‐ and G3BP1‐positive foci (Figure S2A–D, Supporting Information). These findings collectively demonstrate that hypoxia induces SG formation in NIH/3T3 cells. Additionally, hypoxia also induced the nuclear export of HMGB1, similar to TIAR (Figure 1E,F). Nuclear‐cytoplasmic fractionation, followed by immunoblotting, was performed to validate the immunofluorescence observations, which confirmed that hypoxia indeed promoted the nuclear export of both HMGB1 and TIAR (Figure 1G,H).

### 2.2 HMGB1 Mediates Hypoxia‐Induced Nuclear Export of TIAR and Promotes SG Formation

**Figure 2:**
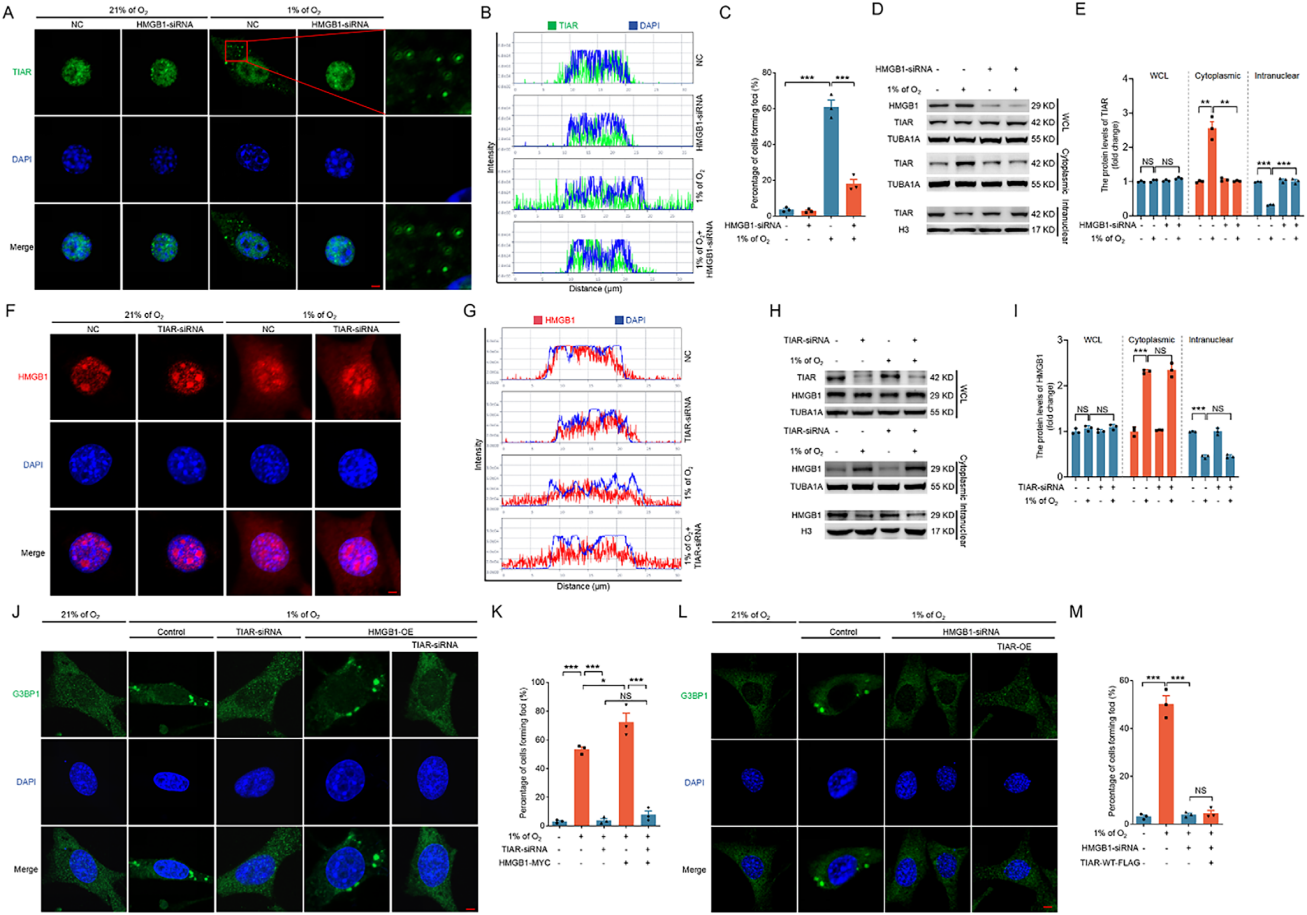
HMGB1 regulates the nuclear export of TIAR for the formation of SGs under hypoxia. A–E) NIH/3T3 cells transfected with HMGB1 siRNA or scramble control siRNA for 24 h were cultured for 2 h under normoxia (21% of O_2_) or hypoxia (1% of O_2_) conditions. Cells were then collected for immunofluorescence assay to observe the subcellular localization of TIAR (A). The fluorescence intensity curve shows the distribution of TIAR (green) and DAPI (blue) along cells (B). Scale bar = 5 µm. The proportion of cells containing TIAR foci was counted by laser confocal microscopy (C). Cells were collected for measuring TIAR expression via western blot analysis of cytoplasmic and nuclear fractions (D), and the data were quantified (E). F–I) Cells transfected with TIAR siRNA or scramble control siRNA for 24 h were cultured for 2 h under normoxia (21% of O_2_) or hypoxia (1% of O_2_) conditions. Cells were then collected to observe the subcellular localization of G3BP1 (C) using immunofluorescence assay (F). The fluorescence intensity curve shows the distribution of HMGB1 (red) and DAPI (blue) along cells (G). Immunoblotting assays were conducted to measure the protein level of HMGB1 after nuclear and cytoplasmic extraction (H) and quantified (I). J,K) Cells transfected with TIAR siRNA before HMGB1 overexpression or no HMGB1 overexpression for 24 h were cultured for 2 h under normoxia (21% of O_2_) or hypoxia (1% of O_2_) conditions. G3BP1 was observed by immunofluorescence assay (J). Proportion of cells containing G3BP1 foci counted by laser confocal microscopy (K). L,M) cells were transfected with or without HMGB1 siRNA followed by TIAR overexpression for 24 h, and then cultured under normoxic (21% of O_2_) or hypoxic (1% of O_2_) conditions for 2 h. G3BP1 granules were visualized by immunofluorescence staining (L). Proportion of cells containing G3BP1 foci counted by laser confocal microscopy (M). Data are presented as mean ± s.e.m. (n = 3). *P < 0.05, **P < 0.01, ***P < 0.001; NS, not significant (*P* > 0.05).

The aforementioned results prompted us to investigate whether the nuclear export of TIAR, which is essential for SG assembly, is linked to hypoxia‐induced HMGB1 nuclear export. To fulfill this aim, we employed siRNA to knock down HMGB1 in NIH/3T3 cells, MEF cells, and mouse GCs. As shown in **Figure** **2**A,B, and Figures S3A,B, and S4A,B (Supporting Information), the nuclear export of TIAR was almost entirely inhibited under hypoxic conditions. Additionally, the percentage of cells forming SGs was significantly reduced following HMGB1 knockdown (Figure 2C; Figures S3C and S4C, Supporting Information). Nuclear‐cytoplasmic fractionation and subsequent immunoblotting confirmed that TIAR nuclear export was substantially suppressed under hypoxia when HMGB1 expression was silenced (Figure 2D,E; Figures S3D,E, and S4D,E, Supporting Information). In contrast, TIAR knockdown did not affect the nuclear export of HMGB1 under hypoxic conditions, as indicated by both the immunofluorescence (Figure 2F,G) and immunoblotting (Figure 2H,I) results. To further elucidate the role of HMGB1 in SG formation via TIAR, we knocked down TIAR under hypoxic conditions, which significantly inhibited the formation of G3BP1‐positive foci. Conversely, HMGB1 overexpression markedly promoted G3BP1 aggregation. Notably, TIAR knockdown in HMGB1‐overexpressing cells significantly suppressed the HMGB1‐induced enhancement of G3BP1 aggregation (Figure 2J,K). Moreover, overexpression of TIAR failed to promote G3BP1 granule formation in HMGB1‐depleted cells under hypoxic conditions (Figure 2L,M). These findings unequivocally substantiate the hypothesis that HMGB1 nuclear export plays a pivotal role in the nuclear output of TIAR under hypoxic conditions, thereby facilitating the formation of SGs.

### 2.3 HMGB1 Forms a Complex with TIAR and Facilitates its Nuclear Export to Promote SG Assembly under Hypoxia

**Figure 3:**
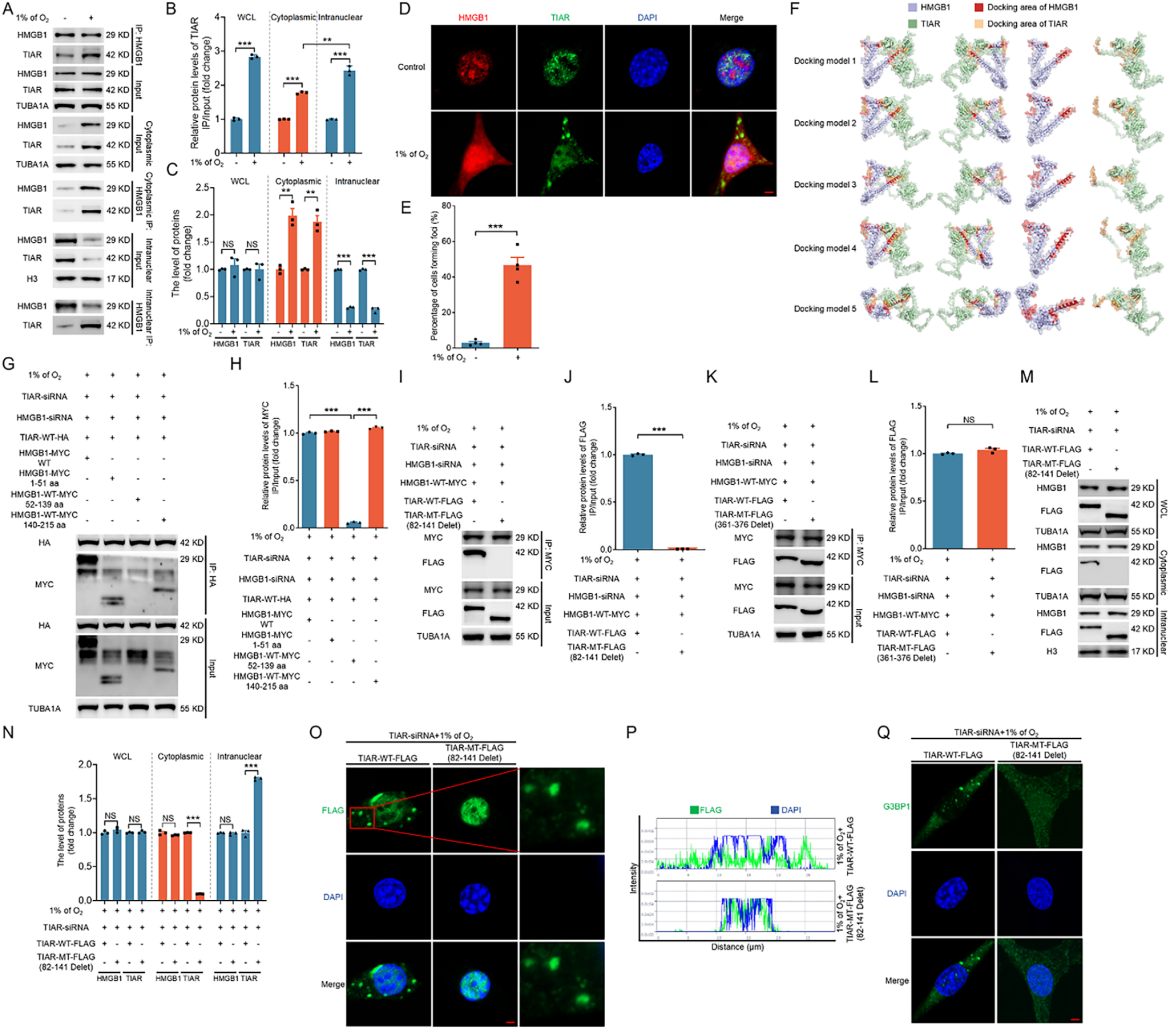
HMGB1 forms a complex with TIAR and promotes the nuclear export of TIAR to form SGs under hypoxia. A–E) NIH/3T3 cells were cultured under normoxic (21% of O_2_) or hypoxic (1% of O_2_) conditions for 2 h. Whole‐cell lysates or nuclear/cytoplasmic fractions were subjected to immunoprecipitation (IP) to assess the interaction between HMGB1 and TIAR (A), and band intensities were quantified (B and C). Immunofluorescence staining was used to assess the nuclear and cytoplasmic distribution of HMGB1 and TIAR (D). The proportion of cells containing TIAR foci was quantified by confocal microscopy (E). F) Interaction modeling of HMGB1 and TIAR was performed using Alphafold2 software, and the top 1–5 docking models are shown according to affinity. G,H) Cells pretreated with HMGB1 and TIAR siRNA were co‐transfected with TIAR‐WT‐HA and HMGB1‐WT‐MYC, HMGB1‐MYC (1–55 aa), HMGB1‐MYC (52–139 aa), or HMGB1‐MYC (140–215 aa) plasmids for 24 h and then cultured for 2 h under hypoxic (1% of O_2_) conditions, followed by IP to assess the interaction between HA and MYC (G), and quantified (H). I‐N, Cells pretreated with HMGB1 and TIAR siRNA were co‐transfected with HMGB1‐WT‐MYC and TIAR‐WT‐FLAG, TIAR‐MT‐FLAG (82–141 aa delete), or TIAR‐MT‐FLAG (361–376 aa delete) plasmids for 24 h and then cultured for 2 h under hypoxic (1% of O_2_) conditions. Cells were collected for detecting the interaction between MYC and FLAG by IP assay (I and K). The cytoplasmic and nuclear fractions of cells were analyzed for detecting protein levels of HMGB1 and FLAG by immunoblotting (M). The protein bands were quantified (J, L, and N). O‐Q, Cells pretreated with TIAR siRNA were transfected with TIAR‐WT‐FLAG or TIAR‐MT‐FLAG (82‐141 aa delete) plasmid for 24 h, cultured for 2 h under hypoxic (1% of O_2_) conditions, and collected to observe the subcellular localization of FLAG (O) and G3BP1 (Q) by immunofluorescence assay. The fluorescence intensity curve shows the distributions of FLAG (green) and DAPI (blue) along the cells (P). Scale bar = 5 µm. Data are presented as mean ± s.e.m. (n = 3). **P < 0.01, ***P < 0.001; NS, not significant (*P* > 0.05).

We examined the interaction between HMGB1 and TIAR under hypoxic conditions to elucidate the molecular mechanism by which HMGB1 regulates the nuclear export of TIAR. Following hypoxia treatment, we observed a significant increase in the binding affinity between HMGB1 and TIAR in both the cytoplasmic and nuclear fractions, as shown in **Figure** **3**A–E. To determine whether HMGB1 and TIAR interact directly, we performed an in vitro immunoprecipitation assay using purified HIS‐TIAR and GST‐HMGB1 proteins (Figure S5, Supporting Information), and the results indicated that they directly interact. And we predicted the 3D structures of both proteins using AlphaFold2 based on their respective amino acid sequences, followed by molecular docking analysis using ClusPro, to further investigate the binding regions between HMGB1 and TIAR. The top five predicted binding poses, ranked by protein binding affinity, are illustrated in Figure 3F, and the corresponding interacting amino acid residues are presented in Table S2 (Supporting Information) using PyMOL software.

We constructed MYC‐tagged WT HMGB1 (HMGB1‐MYC‐WT) and truncated HMGB1 mutants (HMGB1‐MYC 1–51aa, HMGB1‐MYC 52–139aa, and HMGB1‐MYC 140–215aa) to experimentally validate the predicted binding regions. Concurrently, HA‐tagged WT TIAR was overexpressed in cells with knockdown of endogenous TIAR and HMGB1 under hypoxic conditions. IP assays using HA antibodies revealed that TIAR predominantly interacts with the 1–51 aa and 140–215 aa regions of HMGB1, whereas no significant binding occurred with the 52–139 aa region (Figure 3G,H). These findings indicate that the N‐ and C‐terminal regions of HMGB1 are critical for its interaction with TIAR.

Next, we generated FLAG‐tagged WT TIAR (TIAR‐WT‐FLAG) and two deletion mutants (TIAR‐MT‐FLAG 82–141aa deletion and TIAR‐MT‐FLAG 361–376aa deletion) to map the HMGB1 binding sites on TIAR. Following knockdown of endogenous TIAR and HMGB1 under hypoxia, MYC‐tagged HMGB1 and FLAG‐tagged TIAR plasmids were overexpressed, and IP assays using an MYC antibody were performed. The results showed that deletion of the 82–141aa region of TIAR almost completely terminated its interaction with HMGB1, whereas deletion of the 361–376aa region had no effect on HMGB1‐TIAR binding (Figure 3I–L). These findings suggest that the 82–141aa region of TIAR is crucial for its interaction with HMGB1.

We performed nuclear‐cytoplasmic fractionation following overexpression of TIAR‐WT‐FLAG or TIAR‐MT‐FLAG 82–141aa deletion in cells with TIAR knockdown to confirm the role of the HMGB1‐TIAR complex in promoting TIAR nuclear export and subsequent SG formation under hypoxia. The immunoblotting results demonstrated that deletion of the 82–141aa region significantly impaired TIAR nuclear export (Figure 3M,N). Moreover, immunofluorescence analysis revealed that the deletion of the 82–141aa region also completely inhibited SG formation under hypoxic conditions (Figure 3O–Q).

In addition to hypoxia, other type I stresses, such as sodium arsenite exposure and heat stress, have been reported to be inducers of SG formation.^[^ ^2^ ^]^ We exposed NIH/3T3 cells to 200 µM of sodium arsenite or heat shock at 43 °C to determine whether HMGB1 similarly regulates TIAR nuclear export and SG formation under these conditions. Immunofluorescence assays confirmed that both stressors promoted TIAR translocation from the nucleus to the cytoplasm and induced SG formation (Figures S6A–C and S7A–C, Supporting Information). Nuclear‐cytoplasmic fractionation further revealed that sodium arsenite exposure and heat shock also promoted HMGB1 nuclear export (Figures S6D,E, and S7D,E, Supporting Information), similar to the hypoxia response. However, neither sodium arsenite exposure nor heat shock enhanced the interaction between HMGB1 and TIAR (Figures S6F,G, and S7F,G, Supporting Information). Notably, HMGB1 knockdown partially inhibited TIAR nuclear export under sodium arsenite exposure or heat shock but had no effect on SG formation (Figures S6H–J and S7H–J, Supporting Information). These results suggest that unlike hypoxia, other type I stresses such as sodium arsenite exposure and heat shock induce TIAR nuclear export and SG formation independently of HMGB1.

### 2.4 Lactate is Essential for HMGB1‐Mediated TIAR Nuclear Export and SG Formation under Hypoxia

**Figure 4:**
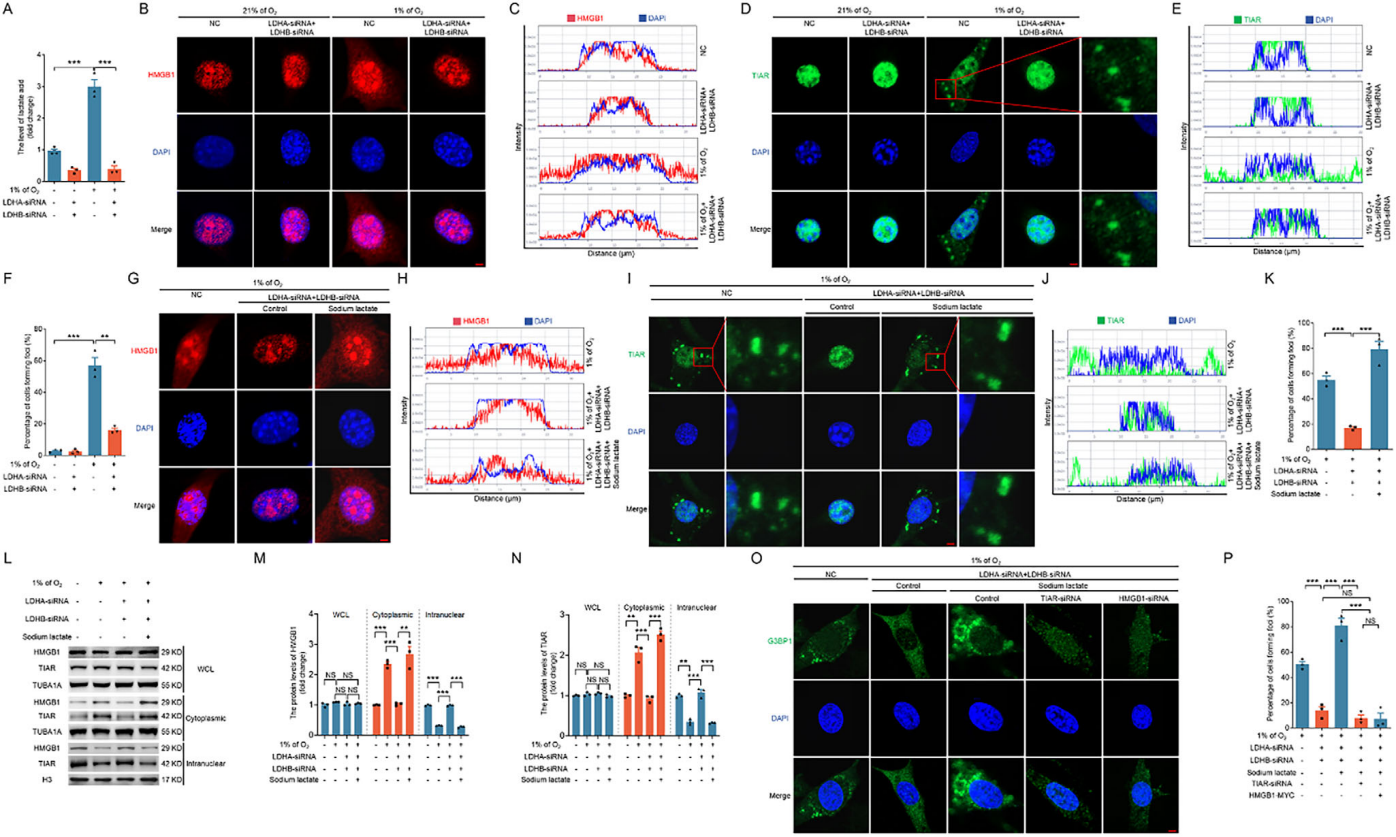
Lactic acid is essential for HMGB1 to regulate TIAR nuclear export to form SG under hypoxia. A–D) NIH/3T3 cells transfected with LDHA/LDHB siRNAs or scramble control siRNA for 24 h and cultured under hypoxia (1% of O_2_) for an additional 2 h and then collected for detecting the levels of lactic acid (A) or for immunofluorescence assay to observe the subcellular localization of HMGB1 (B) or TIAR (D). The fluorescence intensity curve shows the distribution of HMGB1 (red) (C) or TIAR (green) (E) and DAPI (blue) along the cells. Scale bar = 5 µm. The number of foci formed by TIAR was counted using a laser confocal microscope (F). G–N) Cells transfected with LDHA/LDHB siRNAs or scramble control siRNA for 24 h were treated with or without 1 mM of sodium lactate and cultured under hypoxia (1% of O_2_) for an additional 2 h. The subcellular localization of HMGB1 (G) or TIAR (I) was observed by immunofluorescence assay. The fluorescence intensity curve shows the distributions of HMGB1 (red) (H) or TIAR (green) (J) and DAPI (blue) along cells. Scale bar = 5 µm. The proportion of cells that contain foci formed by TIAR was counted by laser confocal microscopy (K). The cytoplasmic and nuclear fractions of cells were analyzed for detecting protein levels of HMGB1 and TIAR by immunoblotting (L). The bands were quantified (M and N). O and P, Cells co‐transfected with LDHA/LDHB siRNAs and TIAR siRNA or HMGB1 siRNA for 24 h were treated with or without 1 mM of sodium lactate and cultured under hypoxia (1% of O_2_) for 2 h and then collected for immunofluorescence assay to observe G3BP1 (O) and count the proportion of cells containing G3BP1 foci by laser confocal microscopy (P). Data are presented as mean ± s.e.m. (n = 3). **P < 0.01, ***P < 0.001; NS, not significant (*P* > 0.05).

To ascertain the underlying cause of hypoxia‐induced nuclear export of the HMGB1‐TIAR complex, we measured intracellular lactate levels at 0, 0.5, 1, and 2 h after hypoxic treatment and observed a significant increase in lactate levels at 1 h (Figure S8, Supporting Information). Furthermore, knockdown of LDHA and LDHB effectively suppressed lactate production (**Figure** **4**A). Knockdown resulted in a marked reduction in hypoxia‐induced nuclear export of both HMGB1 and TIAR (Figure 4B–E) and significantly suppressed SG formation (Figure 4F). Strikingly, supplementation with sodium lactate in LDHA‐ and LDHB‐knockdown NIH/3T3 cells, MEF cells, or mouse GCs restored nuclear export of HMGB1 and TIAR (Figure 4G–J; Figures S9A,B, and S10A,B, Supporting Information) and re‐established SG formation (Figure 4K; Figures S9C, and S10C, Supporting Information) under hypoxic conditions. These observations were validated through immunoblotting following nucleoplasmic fractionation (Figure 4L–N; Figures S9D–F, and S10D–F, Supporting Information). However, although sodium lactate treatment under normoxic conditions promoted the nuclear export of both HMGB1 and TIAR (Figure S11A–C, Supporting Information), it did not induce SG formation (Figure S11D,E, Supporting Information), indicating that additional stress signals are required to trigger SG assembly. Additionally, knockdown of either TIAR or HMGB1 significantly inhibited SG formation in cells that were supplemented with sodium lactate following LDHA and LDHB knockdown under hypoxia (Figure 4O,P). To determine whether sodium lactate or hypoxia promotes HMGB1 nuclear export rather than inhibiting its nuclear import, we treated cells with Leptomycin B (LMB, a specific inhibitor of CRM1) and N‐acetylcysteine (NAC), both of which inhibit HMGB1 nuclear export, followed by sodium lactate or hypoxia treatment. Nuclear‐cytoplasmic fractionation and Western blot analysis revealed that, under LMB or NAC treatment, sodium lactate or hypoxia did not increase cytoplasmic HMGB1 levels or reduce nuclear HMGB1 levels (Figure S12A–D, Supporting Information). These results confirm that sodium lactate and hypoxia specifically facilitate HMGB1 nuclear export rather than inhibiting its nuclear import. Collectively, these findings highlight lactate as a key regulator of hypoxia‐induced nuclear export of the HMGB1‐TIAR complex, thereby driving SG formation.

### 2.5 Hypoxia‐Induced HMGB1 Lactylation Facilitates Nuclear Export of the HMGB1‐TIAR Complex

**Figure 5:**
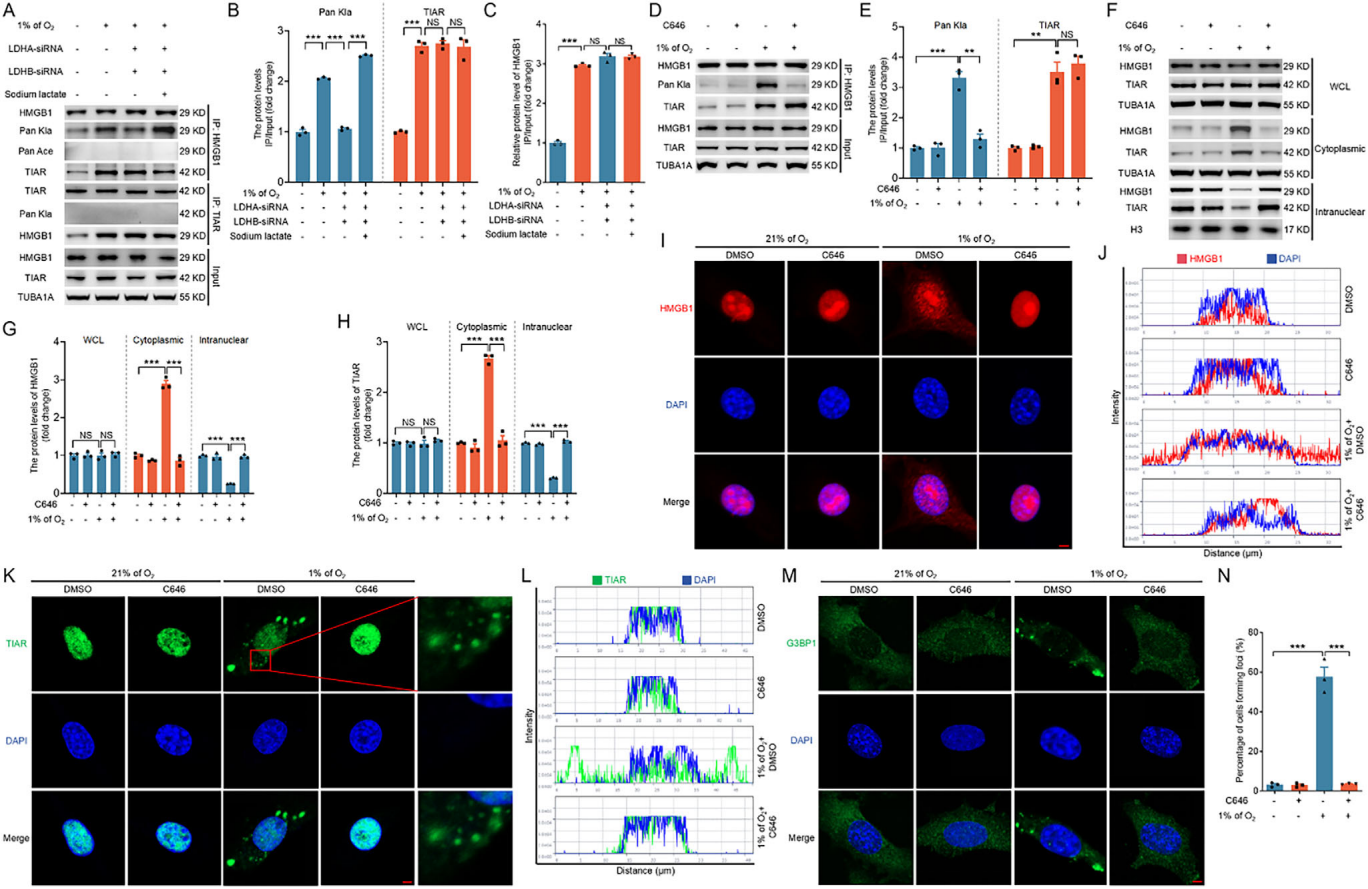
Hypoxia facilitates the lactylation of HMGB1 for the promotion of nuclear export of HMGB1‐TIAR complex. A–C) NIH/3T3 cells transfected with LDHA/LDHB siRNAs or scramble control siRNA for 24 h were treated with or without 1 mM of sodium lactate and cultured under normoxia (21% of O_2_) or hypoxia (1% of O_2_) for 2 h. The cells were collected to determine the interaction between HMGB1 and TIAR, as well as the lactylation and acetylation levels of HMGB1 or TIAR via IP (A), and quantified (B and C). D–N) Cells pre‐treated with 10 µM of C646 for 2 h were cultured under normoxic (21% of O_2_) or hypoxic (1% of O_2_) conditions for 2 h. IP assay was conducted to analyze the interaction of HMGB1 with TIAR or Pan Kla (D), and the protein bands were quantified (E). The protein levels of HMGB1 and TIAR in whole cell lysate (WCL) and cytoplasmic and nuclear fractions were measured by western blot (F) and quantified (G and H). The subcellular localization of HMGB1 (I), TIAR (K), or G3BP1 (M) was observed by immunofluorescence assay. The fluorescence intensity curve shows the distributions of HMGB1 (red) (J) or TIAR (green) (L) and DAPI (blue) along cells. Scale bar = 5 µm. The proportion of cells containing G3BP foci was counted by laser confocal microscopy (N). Data are presented as mean ± s.e.m. (n = 3). **P < 0.01, ***P < 0.001; NS, not significant (*P* > 0.05).

Recent studies have identified lactate‐mediated lactylation as a novel post‐translational modification that modulates protein function.^[^ ^22^ ^]^ In our immunoprecipitation assay, hypoxia significantly enhanced the binding of HMGB1 to TIAR and upregulated HMGB1 lactylation, while no effect was observed on HMGB1 acetylation or TIAR lactylation (**Figure** **5**A–C). Moreover, knockdown of LDHA and LDHB markedly reduced hypoxia‐induced HMGB1 lactylation, but supplementation with sodium lactate restored this modification (Figure 5A–C). These data indicate that the hypoxia‐mediated increase in HMGB1 lactylation is dependent on lactate synthesis, whereas HMGB1‐TIAR binding appears unaffected by lactate or HMGB1 lactylation.

The aforementioned observations prompted us to consider whether lactylation of HMGB1 plays a role in regulating the nuclear export of the HMGB1‐TIAR complex. As shown in Figure 5D–N, inhibition of P300, a key lactylation “writer” by C646 significantly reduced both HMGB1 lactylation and the nuclear export of the HMGB1‐TIAR complex as well as SG formation without affecting the HMGB1‐TIAR interaction. These results suggest that HMGB1 lactylation facilitates hypoxia‐induced nuclear export of the HMGB1‐TIAR complex and subsequent SG formation.

### 2.6 HMGB1 K177 Lactylation Promotes TIAR Nuclear Export and SG Formation

**Figure 6:**
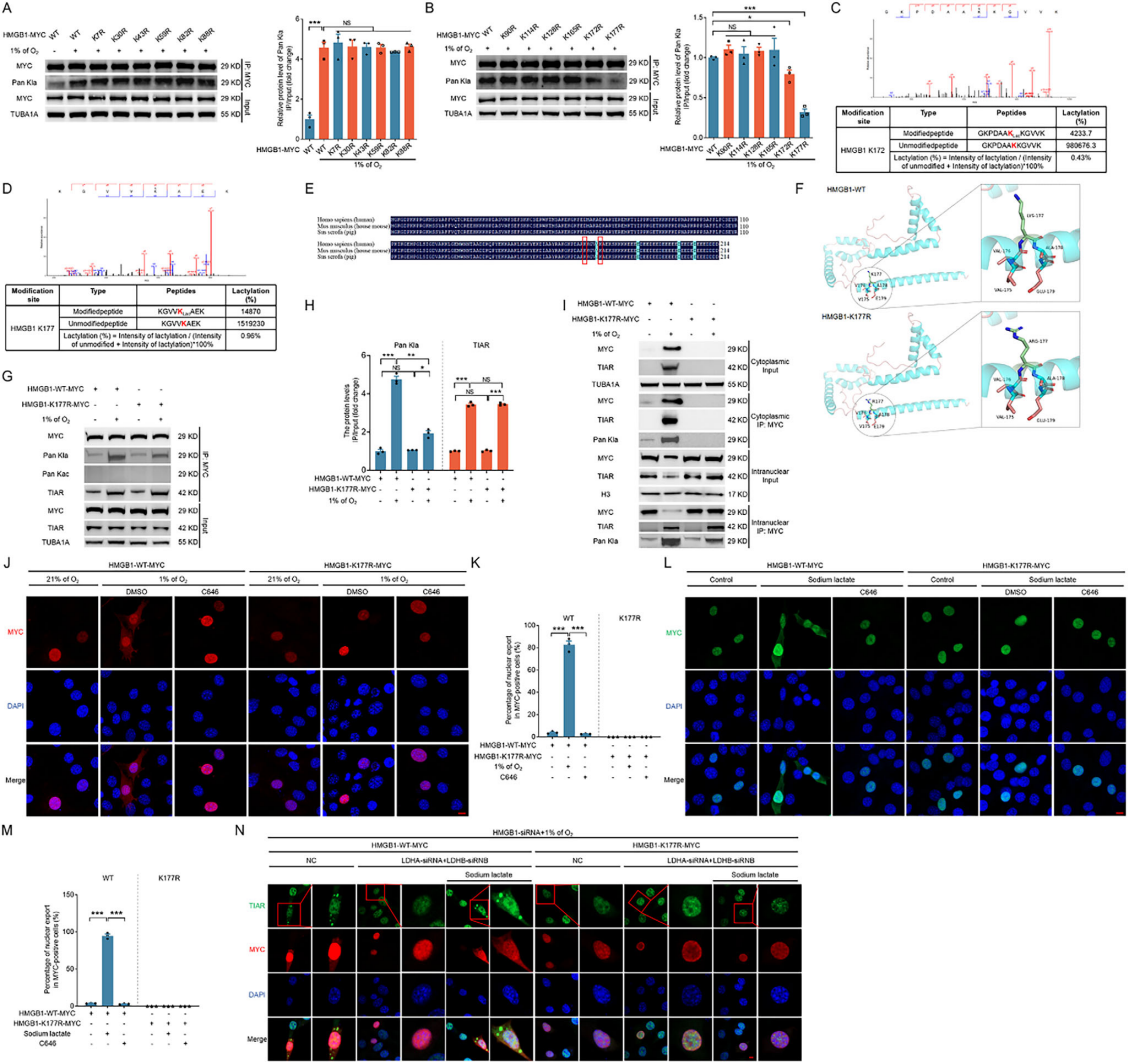
K177 lactylation of HMGB1 promotes nuclear export of TIAR to form SGs. A,B) NIH/3T3 cells overexpressing with vectors encoding MYC‐tagged wild‐type HMGB1 or mutant HMGB1 including K7R, K30R, K43R, K59R, K82R, K88R, K90R, K114R, K128R, K165R, K172R, or K177R for 24 h were cultured under normoxia (21% of O_2_) or hypoxia (1% of O_2_) for 2 h. IP assay was performed to analyze the lactylation level of MYC, and the bands were quantified. C,D) mass spectrometry spectra of HMGB1 K172 and K177 lactylation, and the corresponding calculated proportions of lactylation modification. E) Amino acid sequences of the human, porcine, and murine HMGB1 proteins were compared using DNAMAN software to determine the identity. The amino acids that are circled in the red box are K172 and K177, respectively. F) Comparison of wild‐type and K177R mutant HMGB1 protein structures using Alphafold2 analysis. The stick structures of K177 and R177 are highlighted. G–I) Cells transfected with vectors encoding HMGB1‐WT‐MYC or HMGB1‐K177R‐MYC for 24 h were cultured under normoxia (21% of O_2_) or hypoxia (1% of O_2_) for a further 2 h. IP analysis was performed to determine the interaction between MYC and TIAR, as well as the lactylation and acetylation levels of MYC protein (G). The bands were quantified (H). Cells were harvested to separate nuclear and cytoplasmic fractions, the protein levels of MYC and TIAR were analyzed using immunoblotting, and the interactions between MYC and TIAR proteins and the lactylation levels of MYC were assessed by IP assay (I). J–M) Cells transfected with HMGB1‐WT‐MYC or HMGB1‐K177R‐MYC vectors for 24 h were treated with or without 10 µM of C646 for 2 h and then cultured under normoxia (21% of O_2_) or hypoxia (1% of O_2_) for a further 2 h or cultured with or without 1 mM of sodium lactate for an additional 2 h. Cells were harvested to detect the subcellular localization of MYC by immunofluorescence assay (J and L), and the proportion of cells with cytoplasmic localization of MYC was counted by laser confocal microscopy (K and M). Scale bar = 10 µm. N, Cells co‐transfected with HMGB1 siRNA and LDHA/LDHB siRNAs for 24 h were treated with HMGB1‐WT‐MYC or HMGB1‐K177R‐MYC vectors for 24 h and cultured with or without 1 mM of sodium lactate for a further 2 h and then harvested to observe the subcellular localization of TIAR and HMGB1 using immunofluorescence assay. Scale bar = 5 µm. Data are presented as mean ± s.e.m. (n = 3). *P < 0.05, **P < 0.01, ***P < 0.001; NS, not significant (*P* > 0.05).

We identified the lactylation sites of the HMGB1 protein via mass spectrometry to further investigate how HMGB1 lactylation regulates the nuclear export of the HMGB1‐TIAR complex and promotes SG formation. The analysis revealed 12 lysine lactylation sites on HMGB1: K7, K30, K43, K59, K82, K88, K90, K114, K128, K165, K172, and K177 (Figure S3, Supporting Information). We generated MYC‐tagged WT and mutant HMGB1 plasmids to explore which specific lysine lactylation sites influence the nuclear export of the HMGB1‐TIAR complex under hypoxic conditions. In the mutant plasmids, lysine (K) residues were substituted with arginine (R) to mimic the delactylated state. These constructs were overexpressed in NIH/3T3 cells for 24 h, followed by 2 h of hypoxia treatment. IP using MYC antibodies demonstrated that hypoxia significantly enhanced the lactylation levels of the HMGB1‐WT‐MYC protein (**Figure** **6**A,B). However, mutations at K7, K30, K43, K59, K82, K88, K90, K114, K128, and K165 had no effect on the lactylation of HMGB1 or its hypoxia‐induced nuclear export (Figure 6A,B; Figure S13A–J, Supporting Information). In contrast, the K172R and K177R mutations markedly reduced the lactylation levels of HMGB1 under hypoxic conditions (Figure 6A,B). Mass spectrometry analysis further revealed that K172 and K177 exhibit relatively higher levels of lactylation compared to other lysine residues, with modification ratios of 0.43% (Figure 6C) and 0.96% (Figure 6D), respectively, indicating that K172 and K177 are key sites for HMGB1 lactylation in response to hypoxia. Furthermore, protein sequence homology analysis using DNAMAN software showed that K172 and K177 are highly conserved across species, including in humans, mice, and pigs (Figure 6E), suggesting that the mechanism of HMGB1 lactylation at these residues may be evolutionarily conserved.

**Figure 7:**
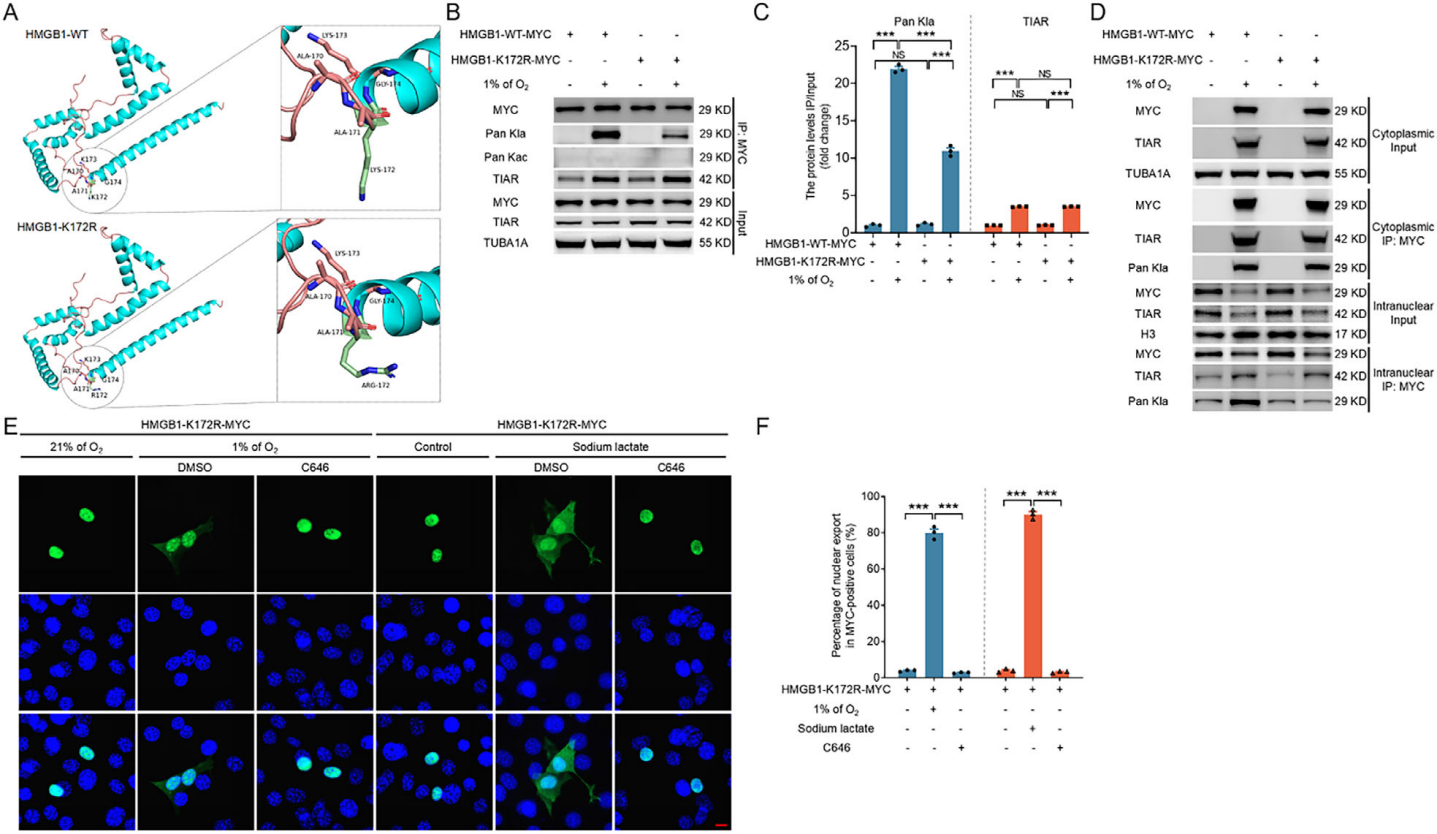
Nuclear export of HMGB1 under hypoxia is not regulated by K172 lactylation of HMGB1. A) Comparison of wild‐type and K172R mutant HMGB1 protein structures using Alphafold2 analysis, and the stick structures of K172 and R172 are highlighted. B–D) NIH/3T3 cells transfected with vectors encoding HMGB1‐WT‐MYC or HMGB1‐K172R‐MYC for 24 h were cultured under normoxia (21% of O_2_) or hypoxia (1% of O_2_) for a further 2 h. IP analysis of the interaction between MYC and TIAR and analysis of the lactylation and acetylation levels of MYC protein were performed (B). The protein bands were quantified (C). Cells were harvested to separate nuclear and cytoplasmic fractions. The protein levels of MYC and TIAR were analyzed using immunoblotting, and the interactions between MYC and TIAR proteins and the lactylation levels of MYC were assessed by IP assay (D). E,F) Cells transfected with HMGB1‐WT‐MYC or HMGB1‐K172R‐MYC vectors for 24 h were treated with or without 10 µM of C646 for 2 h and then cultured under normoxia (21% of O_2_) or hypoxia (1% of O_2_) for a further 2 h or cultured with or without 1 mM sodium lactate for an additional 2 h. Cells were harvested to detect the subcellular localization of MYC by immunofluorescence assay (E), and the proportion of cells with cytoplasmic localization of MYC was counted by laser confocal microscopy (F). Scale bar = 10 µm. Data are presented as mean ± s.e.m. (n = 3). ***P < 0.001; NS, not significant (*P* > 0.05).

We analyzed the 3D structures of wild‐type, K172R, and K177R mutant HMGB1 proteins using Alphafold2 software to rule out the possibility that the nuclear export of HMGB1 is influenced by structural changes resulting from amino acid mutations. The analysis revealed that the K172R and K177R mutations did not alter the overall protein structure, which remained identical to that of WT HMGB1 (Figure 6F and **Figure** **7**A). This finding indicates that the K172R and K177R mutations do not affect the structural integrity of HMGB1. To further validate that K177 is the critical lactylation site on HMGB1 and that p300 functions as the “writer” responsible for this modification, we co‐transfected cells with p300 and either wild‐type HMGB1 or the K177R mutant. Immunoprecipitation analysis revealed that p300 overexpression significantly increased lactylation level of wild‐type HMGB1, whereas substitution of K177 with arginine markedly reduced the lactylation level of HMGB1, although the modification was not completely abolished (Figure S14A,B, Supporting Information). This residual signal likely reflects lactylation at other lysine residues, such as K172. Consistent with the results of the C646 treatment shown in Figure 5, K172R or K177R mutation had no effect on HMGB1 binding to TIAR under hypoxia when they inhibited the lactylation of the HMGB1 protein (Figure 5F, Figure 6G,H, and Figure 7B,C), further confirming that the hypoxia‐promoted upregulation of HMGB1 binding to TIAR was independent of HMGB1 lactylation.

This finding indicates that the mechanism by which HMGB1‐TIAR binding is upregulated under hypoxia remains unclear. Thus, whether HMGB1 lactylation at K172 and K177 is involved in the nuclear export of the HMGB1‐TIAR complex has yet to be determined. To investigate this involvement further, we overexpressed WT HMGB1 (HMGB1‐WT‐MYC), HMGB1‐K172R‐MYC, and HMGB1‐K177R‐MYC in NIH/3T3 cells, followed by 2 h of hypoxia treatment. Nucleocytoplasmic fractionation and IP assays revealed that hypoxia significantly enhanced the lactylation and nuclear export of the HMGB1‐TIAR complex in cells expressing WT HMGB1. In contrast, K177R mutation suppressed the lactylation of HMGB1, resulting in a near‐complete inhibition of MYC‐TIAR complex exit from the nucleus (Figure 6I). However, although K172R mutation reduced HMGB1 lactylation, it had no effect on the nuclear export of the HMGB1‐TIAR complex (Figure 7D). Moreover, nuclear and cytoplasmic lactylation analyses showed that hypoxia significantly promoted the nuclear lactylation of HMGB1‐K177R‐MYC but not HMGB1‐K172R‐MYC (Figure 6I and Figure 7D). This provides further evidence that K177, rather than K172, regulates the nuclear export of the HMGB1‐TIAR complex under hypoxia.

NIH/3T3 cells transfected with HMGB1‐WT‐MYC, HMGB1‐K172R‐MYC, or HMGB1‐K177R‐MYC plasmids were subjected to hypoxic or sodium lactate treatment to substantiate the aforementioned findings. The results of the immunofluorescence analysis, presented in Figure 6J–M, and Figure 7E,F, demonstrate that hypoxia or sodium lactate exposure significantly promoted the nuclear export of HMGB1‐WT‐MYC and HMGB1‐K172R‐MYC. However, this promotion was completely inhibited by the addition of C646. Notably, hypoxia or sodium lactate exposure failed to promote the nuclear export of HMGB1‐K177R‐MYC.

Recent studies have demonstrated that LPS promotes the nuclear export of HMGB1 via two distinct post‐translational modifications: lactylation and acetylation.^[^ ^20^ ^]^ In addition, as shown in Figures S6 and S7 (Supporting Information), both sodium arsenite exposure and heatshock promote the nuclear export of HMGB1. We conducted additional experiments to rule out the possibility that the K177R mutation itself hinders HMGB1 nuclear export. NIH/3T3 cells transfected with the HMGB1‐K177R‐MYC plasmid were treated with LPS or sodium arsenite or subjected to heat shock. Following nucleocytoplasmic fractionation, IP and immunoblotting analyses revealed that LPS significantly promoted the acetylation of HMGB1‐K177R‐MYC but had no effect on lactylation (Figure S15A,B, Supporting Information). LPS also substantially enhanced HMGB1‐K177R‐MYC nuclear export (Figure S15A,C, Supporting Information), as confirmed by the immunofluorescence results (Figure S15D,E, Supporting Information). Testing of sodium arsenite and heat shock treatment revealed that sodium arsenite but not heat shock treatment significantly increased HMGB1‐K177R‐MYC lactylation (Figures S16A,B, and S17A,B, Supporting Information). However, both sodium arsenite exposure and heat shock enhanced the nuclear export of HMGB1‐K177R‐MYC (Figures S16A,C–E and S17A,C–E, Supporting Information). These findings indicate that the failure of HMGB1‐K177R‐MYC to exit the nucleus under hypoxic or sodium lactate treatment is not due to structural changes caused by the K177R mutation but due to the prevention of HMGB1 lactylation by K177R.

Previous studies have shown that CRM1 (also known as Exportin‐1) mediates the nuclear export of acetylated HMGB1.^[^ ^23^ ^]^ To determine whether CRM1 also contributes to the export of lactylated HMGB1, we first examined the interaction between HMGB1 and CRM1 under normoxic and hypoxic conditions. We found that hypoxia markedly enhanced the interaction between HMGB1 and CRM1 (Figure S18A,B, Supporting Information), whereas mutation of lysine 177 to arginine (K177R) significantly impaired this interaction under hypoxia (Figure S18C,D, Supporting Information). These results suggest that lactylation at K177 is critical for the hypoxia‐induced binding of HMGB1 to CRM1. To further confirm the role of CRM1 in promoting the nuclear export of lactylated HMGB1, we treated cells with the CRM1‐specific inhibitor Leptomycin B under hypoxia. We found that Leptomycin B did not alter HMGB1 lactylation levels or its interaction with TIAR, but it significantly inhibited the nuclear export of lactylated HMGB1 and TIAR (Figure S18E–I, Supporting Information). Together, these findings demonstrate that CRM1 is involved in the hypoxia‐induced nuclear export of lactylated HMGB1.

NIH/3T3 cells were transfected with either HMGB1‐WT‐MYC or HMGB1‐K177R‐MYC plasmids following knockdown of endogenous HMGB1 to confirm that K177 lactylation regulates the nuclear export of the HMGB1‐TIAR complex and promotes the formation of SGs. As shown in the immunofluorescence assay (Figure 6N), hypoxia significantly promoted the nuclear export of both MYC and TIAR, as well as the formation of SGs in MYC‐positive cells. This effect was suppressed when LDHA and LDHB were knocked down but was restored by the addition of sodium lactate, which reinstated the nuclear export of MYC and TIAR and the formation of SGs. However, in cells overexpressing the HMGB1‐K177R‐MYC plasmid, neither MYC nor TIAR exited the nucleus in MYC‐positive cells, and as a result, SGs failed to form (Figure 6N). These results suggest that lactylation at K177 is crucial for the nuclear export of the HMGB1‐TIAR complex and subsequent SG formation.

### 2.7 In Vivo Validation of HMGB1 Lactylation in Regulating TIAR Nuclear‐to‐Cytoplasmic Translocation and SG Formation

**Figure 8:**
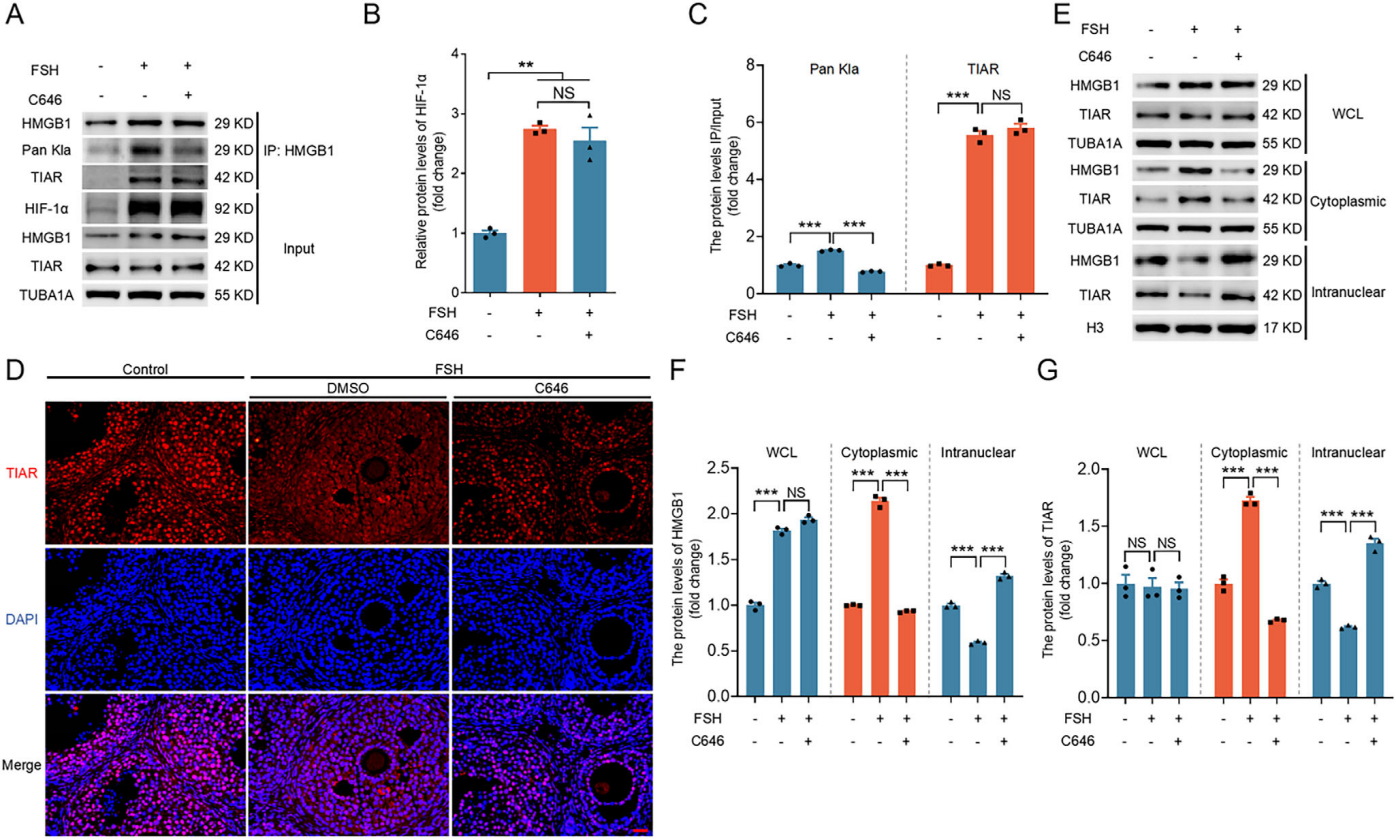
In vivo validation shows that lactylation of HMGB1 regulates TIAR translocation from the nucleus to the cytoplasm to form SGs. A–F) GCs were collected to measure the levels of HIF‐1α, HMGB1, and TIAR by immunoblotting (A). IP assay was performed to determine the lactylation levels of HMGB1 and the interaction between HMGB1 and TIAR (A), and the bands were quantified (B,C). Ovaries were then harvested for immunofluorescence assays to observe the subcellular localization of TIAR in mice following FSH and C646 injections (see Materials and Methods for details) (D). Scale bar = 250 µm. The protein levels of HMGB1 and TIAR in whole cell lysate (WCL) and cytoplasmic and nuclear fractions were measured by western blot (E) and quantified (F and G). Data are presented as mean ± s.e.m. (n = 3). **P < 0.01, ***P < 0.001; NS, not significant (P > 0.05).

We used the well‐established model of FSH‐induced hypoxia in ovarian GCs to verify the molecular mechanism by which the lactylation of HMGB1 regulates the nuclear to cytoplasmic transfer of TIAR for the formation of SGs in vivo. As shown in **Figure** **8**A, FSH treatment markedly elevated HIF‐1α protein levels in GCs, confirming successful hypoxia induction. IP assays and immunofluorescence staining of GCs and ovarian tissue sections revealed that FSH significantly increased HMGB1 lactylation and its interaction with TIAR while concurrently promoting nuclear export of TIAR and SG formation in the cytoplasm (Figure 8A–D). Nucleocytoplasmic fractionation further demonstrated that FSH substantially enhanced the nuclear export of both HMGB1 and TIAR compared to controls (Figure 8E–G). Mice treated with FSH received continuous injections of the p300 inhibitor C646 for 1 week to directly assess the impact of lactylation in vivo. As shown in Figure 8A, C646 significantly suppressed HMGB1 lactylation under hypoxic conditions. Notably, C646 did not affect HMGB1‐TIAR binding but effectively reduced the nuclear export of both HMGB1 and TIAR, thereby inhibiting cytoplasmic SG formation (Figure 8A–G). These results align with our in vitro findings, underscoring the pivotal role of HMGB1 lactylation in SG formation via TIAR translocation.

## 3 Discussion

HMGB1, a highly conserved non‐histone nuclear protein, contains two nuclear localization sequences (NLS), NLS1 (28–44 aa) and NLS2 (179–185 aa).^[^ ^24^ ^]^ Previous studies have demonstrated that post‐translational modifications (PTMs) such as acetylation, phosphorylation, and N‐glycosylation influence the nuclear export of HMGB1.^[^ ^24^, ^25^ ^]^ For example, acetylation of lysine residues in the NLS region induced by LPS has been shown to affect HMGB1 subcellular localization.^[^ ^26^ ^]^ In 2014, Lu et al. reported that LPS increases acetylation at multiple lysine residues, including K43, K179, and K181, with the acetylation of NLS residues identified as key regulators of HMGB1 nuclear export.^[^ ^27^ ^]^ Hwang et al. found that LPS‐induced acetylation at K28, K29, and K30 disrupts the binding of HMGB1 to Sirt1, further facilitating its export from the nucleus.^[^ ^23^ ^]^ In addition to acetylation, LPS also promotes the phosphorylation of serine residues located in the NLS, which mediates the nuclear export of HMGB1 via classical protein kinase (cPKC) or calcium/calmodulin dependent protein kinase (CaMK) IV.^[^ ^25^, ^28^ ^]^ N‐glycosylation at N37, N134, and N135 under endoplasmic reticulum (ER) stress has also been implicated in promoting nuclear export by inhibiting the DNA‐binding ability of HMGB1.^[^ ^25b^ ^]^ Moreover, the redox states of C23 and C45 have been linked to the translocation of HMGB1 from the nucleus to the cytoplasm.^[^ ^29^ ^]^

In this study, we identified lactylation as a novel PTM that regulates HMGB1 nuclear export under hypoxic conditions. Using liquid chromatography‐tandem mass spectrometry (LC‐MS), we discovered lactylation modifications at 12 lysine residues in HMGB1 (K7, K30, K43, K59, K82, K88, K90, K114, K128, K165, K172, and K177). Among these, K177 emerged as a critical site, with the K177R mutation almost entirely inhibiting HMGB1 nuclear export under hypoxia. Although K172 is also sensitive to hypoxia‐induced lactylation, the K172R mutation did not influence HMGB1 nuclear translocation. Similarly, K43, located within the NLS1 region, also underwent lactylation under hypoxia, but its mutation (K43R) only partially reduced without completely blocking HMGB1 export. Given the location of NLS1 within the A‐box structural domain responsible for DNA binding, it is plausible that the K43R mutation affects the DNA‐binding ability of HMGB1, which in turn modulates its nuclear export capacity.

It is important to note that acylation, which includes both lactylation and acetylation, shares many similarities. Both modifications occur on lysine residues, utilizing shared acyltransferases and deacylases. In this study, modification‐specific mass spectrometry analysis revealed that K43 of HMGB1 undergoes lactylation. According to previous reports, K43 can also be acetylated.^[^ ^27^ ^]^ However, we did not detect HMGB1 acetylation under hypoxic conditions. This suggests that lactylation may be a specific acylation modification regulating HMGB1 export in response to hypoxia.

In addition, although the K177R mutation inhibited the lactylation of HMGB1, it remains possible that this amino acid mutation might affect the nuclear export of HMGB1 by altering the protein's structure rather than solely inhibiting lactylation. In this study, we compared the protein structures of both HMGB1 WT and HMGB1 (K177R) using AlphaFold2 and found that the K177R mutation does not affect the overall protein structure of HMGB1. It is noteworthy that LPS promotes the acetylation of HMGB1 (K177R) and facilitates its export from the nucleus, as well as that stressors such as sodium arsenite exposure and heat shock also promote the nuclear translocation of HMGB1 (K177R). This suggests that HMGB1 (K177R) retains the ability to be acetylated and exported from the nucleus, further ruling out the possibility that acetylation at K177 or the K‐R mutation itself impairs the nuclear export of HMGB1.

As a major chromatin‐associated protein, HMGB1 is well established for its interactions with nuclear proteins in regulating DNA transcription and for its extracellular function as an immunomodulatory cytokine mediator.^[^ ^30^ ^]^ Our study reveals for the first time a novel function of HMGB1 in the promotion of SG formation, a complex process that includes translation arrest, aggregation of ribonucleoproteins (RNPs), protein‐protein interactions, and the recruitment of non‐RNA‐bound proteins.^[^ ^31^ ^]^ In mammalian cells, SGs are formed through the phosphorylation of eIF2α, which inhibits translation initiation, leading to mRNA dissociation from ribosomes and subsequent aggregation of proteins to form SGs.^[^ ^11^, ^32^ ^]^ This process is typically induced under environmental stress and reversed upon removal of the stress.^[^ ^10^ ^]^ Additionally, several signaling pathways, including RAS gene mutations, mTORC1 activation, and glycolysis, have been reported to regulate SG formation.^[^ ^33^ ^]^ The assembly of SGs depends on nucleating proteins with intrinsically disordered domains that facilitate phase separation.^[^ ^4b^ ^]^ These nucleating proteins can be induced even under non‐stress conditions in vitro.^[^ ^34^ ^]^

Previous studies have largely focused on characterizing these proteins and their post‐translational modifications. For instance, under heat stress, Nien‐Pei Tsai et al. found that growth factor receptor 7 (Grb7), an RNA‐binding translational regulator, directly interacts with HuR to mediate SG formation.^[^ ^35^ ^]^ In addition, phosphorylation of TIAR‐2, FMRP, and FUS, as well as methylation of DDX4 and hnRNPA2 and acetylation of DDX3X, is also critical in regulating the formation of SGs.^[^ ^36^ ^]^ However, few relevant studies have investigated the role of non‐SG proteins that assist in the aggregation of SG components. In this study, we elucidated a novel mechanism wherein lactylated HMGB1 transports TIAR proteins to the cytoplasm, providing the material basis for hypoxia‐induced SG formation. This finding uncovers a new function of HMGB1 as an essential protein in SG assembly, extending knowledge of functioning beyond that of the core SG components.

TIAR is a key component of SGs.^[^ ^10^ ^]^ Under stress conditions, SGs form exclusively in the cytoplasm, whereas TIAR is primarily localized within the nucleus.^[^ ^37^ ^]^ This leads to consideration of how various SG components, including TIAR, are translocated to the cytoplasm under stress. Previous studies have reported several mechanisms for the nuclear export of TIAR. TIAR contains three RNA recognition motifs (RRMs), with RRM2 playing a crucial role in regulating nuclear localization and RRM3 being essential for its export to the cytoplasm.^[^ ^38^ ^]^ Zhang et al. demonstrated that RRM2 is the key structural domain that retains TIAR in the nucleus and that mutating this region disrupts TIAR's ability to remain nuclear‐localized.^[^ ^39^ ^]^ Moreover, RRM3 mutations impair the export of TIAR to the cytoplasm under stress, highlighting the distinct roles of these two domains.^[^ ^39^ ^]^ Kedersha et al. found that disrupting TIAR's RNA‐binding ability by mutating RRM domains results in abnormal cytoplasmic accumulation of TIAR, which in turn inhibits proper SG formation.^[^ ^11^ ^]^ Our study revealed a novel mechanism by which HMGB1 mediates the nuclear export of TIAR specifically under hypoxic conditions. We found that HMGB1 binds to the 82–141 aa region of TIAR, a sequence entirely contained within RRM2 (87–185 aa), and facilitates the translocation of TIAR to the cytoplasm. This novel interaction between HMGB1 and RRM2 reveals a previously unrecognized molecular mechanism for TIAR nuclear export.

Type I stresses, such as hypoxia, heat stress, and arsenite exposure, are known to induce SG formation,^[^ ^2^ ^]^ leading us to investigate whether the same mechanism is involved in TIAR nuclear export during heat stress and arsenite exposure. Our findings revealed that HMGB1 and TIAR maintain a basal level of interaction, and regardless of the stress type, HMGB1 is exported from the nucleus, facilitating the export of TIAR. These findings suggest that HMGB1‐mediated TIAR nuclear export is a constitutive mechanism under stress conditions. However, unlike hypoxia, neither heat stress nor arsenite exposure enhanced HMGB1‐TIAR binding. When HMGB1 was knocked down to disrupt the HMGB1‐TIAR interaction, most of the TIAR was still able to leave the nucleus to form SGs. This indicates that hypoxia employs a unique mechanism for TIAR nuclear export that is primarily dependent on HMGB1, whereas other types of stress trigger TIAR nuclear export through alternative mechanisms that remain to be elucidated. Additionally, because TIAR does not undergo lactylation and the use of C646 did not affect the HMGB1‐TIAR interaction under hypoxia, we can conclude that hypoxia‐enhanced TIAR binding to HMGB1 is independent of lactylation and that the underlying mechanism thus requires further investigation. Moreover, it remains to be explored whether lactylation sites that do not affect HMGB1 nuclear export play other functional roles.

Hypoxia is a central feature of numerous physiological and pathological processes, including tumorigenesis, neurodegenerative diseases, exercise‐induced muscle hypoxia, high‐altitude adaptation, ischemia‐reperfusion injury, and stroke.^[^ ^40^ ^]^ As a potent type I cellular stressor, hypoxia induces the formation of SGs, which serve as key adaptive structures that help cells cope with environmental challenges.^[^ ^2^ ^]^ They regulate stress responses by sequestering signaling molecules such as astrin, TOR, RACK1, and TRAF2, thereby modulating downstream pathways and suppressing apoptosis under adverse conditions.^[^ ^2^, ^6^, ^41^ ^]^ In addition, SGs enhance the innate immune responses and antiviral defenses by enriching immune effectors like TRAF2, RIG‐1, PKR, OAS, and RNaseL.^[^ ^4^, ^42^ ^]^ Disruption of SG formation has been linked to the pathogenesis of several degenerative diseases, including amyotrophic lateral sclerosis (ALS), frontotemporal lobar degeneration (FTLD), and certain myopathies.^[^ ^43^ ^]^ In cancer, SG dynamics have been implicated in tumor progression and chemoresistance. For instance, YB‐1 overexpression in human sarcomas promotes G3BP1 expression and SG formation, contributing to metastasis and poor prognosis,^[^ ^44^ ^]^ while chemotherapeutics like Bortezomib and 5‐Fluorouracil facilitates SG formation to protect tumor cells from apoptosis.^[^ ^45^ ^]^ HMGB1 has emerged as a critical mediator in hypoxia‐related diseases and cancer.^[^ ^46^ ^]^ Under low‐oxygen conditions, HMGB1 becomes post‐translationally modified and released or exported from the nucleus, where it can provoke inflammation in models such as collagen‐induced arthritis,^[^ ^47^ ^]^ foster mitochondrial biogenesis in pancreatic tumors,^[^ ^48^ ^]^ and exacerbate ischemia–reperfusion injury in liver and heart.^[^ ^49^ ^]^ In this study, we show that hypoxia drives lactylation of HMGB1 at lysine 177, which in turn promotes TIAR nuclear export and SG assembly in NIH/3T3 fibroblasts, MEFs, and granulosa cells. We further validate this mechanism in vivo using an FSH‐induced follicular hypoxia model, shedding light on how granulosa cells adapt to the low‐oxygen microenvironment of folliculogenesis. These findings identify HMGB1 as a novel regulator of SG dynamics under hypoxia and suggest that manipulation of HMGB1 lactylation may modulate both inflammatory and stress‐response pathways in hypoxia‐related pathologies, including cancer. We recognize that our primarily in vitro and ex vivo hypoxia models may not fully capture the complexity of living tissues. Future studies employing hypoxic tumor cell lines, in vivo cancer or ischemia models, and clinical samples will be essential to determine the broader physiological and pathological relevance of HMGB1 K177 lactylation‐mediated SG formation—and to explore its potential as a therapeutic target in hypoxia‐associated diseases.

**Figure 9:**
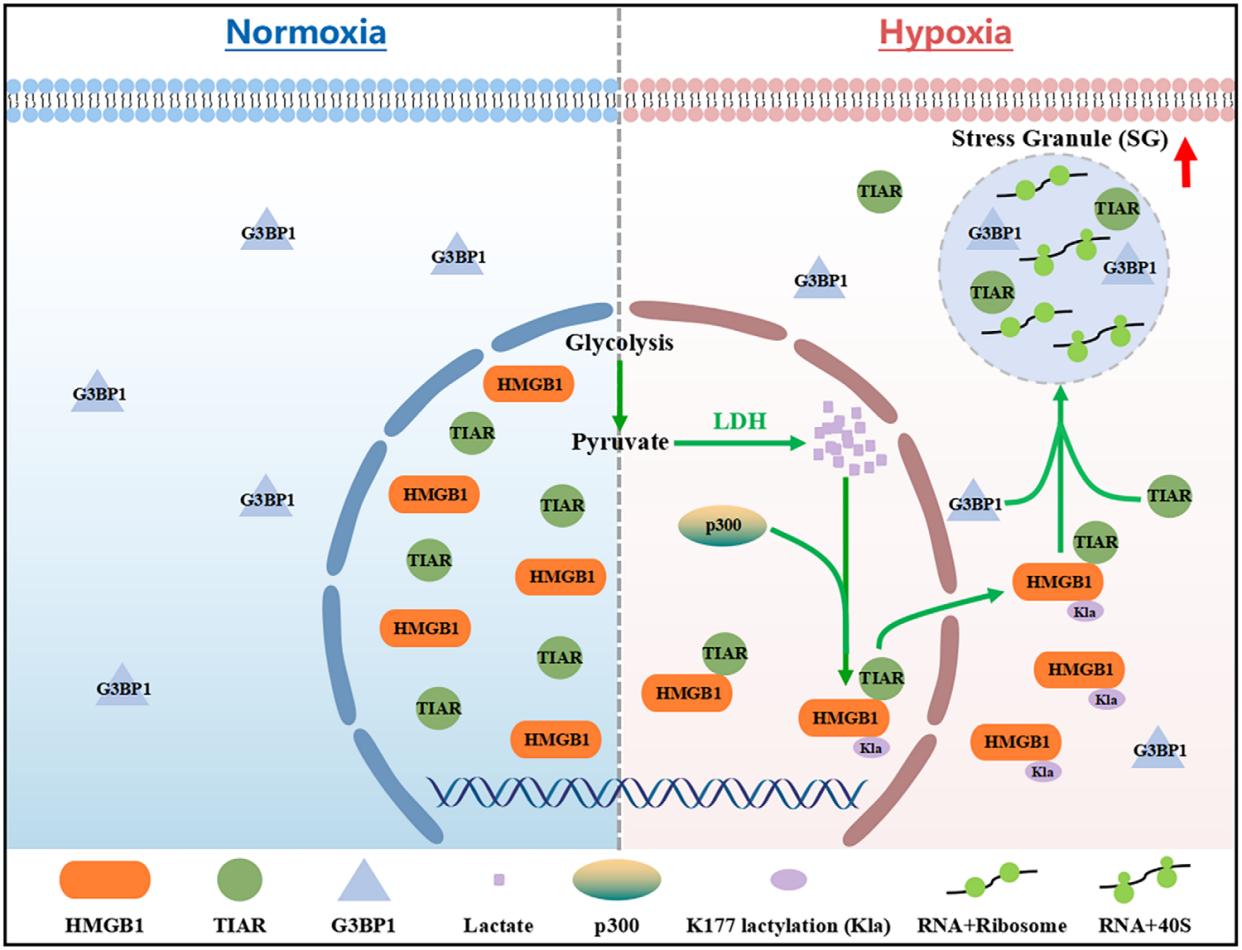
Schematic model illustrating the regulation of the formation of hypoxic SGs by the lactylation of HMGB1. Under normoxic conditions, G3BP1 and TIAR, the key proteins for the formation of SGs, were diffusely distributed in the cytoplasm and nucleus, respectively. Moreover, HMGB1 was mainly distributed in the nucleus. When cells were exposed to hypoxic stress, the binding of HMGB1 and TIAR increased, as did lactate production. This resulted in the lactylation of HMGB1 at K177, which was catalyzed by p300. This modification led to the nuclear export of the HMGB1‐TIAR complex. Subsequently, TIAR was localized in the cytoplasm, where it formed SGs in conjunction with G3BP1, RNP, 40S, and RNA.

In summary, this study uncovered a novel mechanism of HMGB1 nuclear export driven by lactylation, wherein HMGB1 binds to TIAR and transports it to the cytoplasm, facilitating SG formation under hypoxia (**Figure** **9**). Our findings highlight a previously unrecognized role of lactylation as a critical regulator linking cellular metabolism to stress response mechanisms. This discovery not only enhances our understanding of SG dynamics but also identifies potential therapeutic targets for diseases characterized by impaired stress responses, such as cancer and neurodegenerative disorders.

## 4 Experimental Section

### Reagents and Antibodies

Antibodies against G3BP1 (61559), TIAR (8509), MYC‐tag (2276), FLAG‐tag (14793), HIS‐tag (2365S), and HA‐tag (3724) were obtained from Cell Signaling Technology. HMGB1 (ab190377), secondary antibodies (horseradish peroxidase [HRP]‐conjugated goat anti‐mouse IgG H&L (ab6789) and HRP‐conjugated goat anti‐rabbit IgG H&L (ab6721) were obtained from Abcam. p‐eIF2α (Ser51) (68023‐1‐Ig), eIF2α (11170‐1‐AP), CRM1 (66763‐1‐Ig), GST‐tag (66001‐2‐Ig), TUBA1A (66031‐1‐Ig) and HMGB1 (66525‐1‐Ig) were purchased from Proteintech. H3 (CPA6331) was purchased from Cohesion Biosciences. Rabbit monoclonal anti‐PanKla (PTM‐1401RM) and anti‐PanKace (PTM‐102) were purchased from PTMBIO. Alexa Fluor 488 AffiniPure goat anti‐mouse IgG (ZF‐0512) and Rhodamine AffiniPure goat anti‐rabbit IgG (ZF‐0316) were obtained from ZSGB‐BIO. Liposaccharide (LPS; HY‐D1056) and Leptomycin B (HY‐16909) were obtained from MedChemExpress. C646 (S7152) was purchased from Selleck. N‐Acetylcysteine (A233740) was obtained from AmBeed. Sodium lactate (71718) was purchased from Sigma–Aldrich. Sodium arsenite (XFE2922) was purchased from TMRM.

### Animals and Collection of Ovaries

All animal experiments and treatments followed the recommendations of the Care and Use of Animals in Research and Teaching, and all protocols were approved by the Animal Ethics Committee of Nanjing Agricultural University, China (SYXK2022‐0086). Three‐week‐old female Institute of Cancer Research (ICR) mice (Nanjing Medical University, Animal Breeding Center) were grouped and housed in a temperature‐controlled (22 + 2 °C) room with a light‐dark cycle of 12:12 h (lights on from 7:00 a.m. to 7:00 p.m.) with free access to water and food. The mice were randomly divided into control, follicle‐stimulating hormone (FSH)‐injected, and FSH+C646‐injected groups, each group consisting of 10 mice. Mice in the FSH group were injected intraperitoneally (i.p.) with FSH at 7:00 a.m. for 6 h at a dose of 10 IU. Mice in the control group were injected i.p. with 0.9% saline. Mice in the FSH+C646‐injected group were injected i.p. with C646 (0.2 mg/mouse) at 7:00 p.m. and 7:00 a.m. for 7 days. Forty‐eight hours after the first FSH treatment, mice in each group were sacrificed to collect the ovaries and GCs. Separated GCs from the right ovaries were used for immunoprecipitation, extraction of nuclear and cytoplasmic materials, and western blotting analysis. The left ovaries were fixed with 4% paraformaldehyde for immunofluorescent staining.

### Sample Collection, Cell Culture, and Treatments

NIH/3T3 cells were purchased from Cell Bank, Chinese Academy of Sciences, which had obtained the cells from a highly contact‐suppressed continuous cell line established from National Institutes of Health (NIH) Swiss mouse embryo cultures. Mouse fetal fibroblasts (MEFs) were isolated from mouse embryos between E12.5 and E18.5. Briefly, mouse embryos were minced after removal of limbs, head, tail, and most of the internal organs and digested with trypsin for ≈20 min, centrifuged to remove the supernatant, and resuspended in medium before being seeded into T‐75 cell culture dishes. Stable MEF cells were obtained after 3–4 passages and then preserved for subsequent experiments. NIH/3T3 cells and MEF cells were cultured in Dulbecco's modified eagle medium (DMEM, Gibco) supplemented with 10% fetal bovine serum (FBS; Sigma–Aldrich) and 1% penicillin‐streptomycin (Gibco) at 37 °C in a humidified atmosphere with 5% CO_2_. Primary‐cultured granulosa cells (GCs) were isolated from mouse ovaries by puncturing follicles with 1 ml syringes under a stereoscopic microscope and then cultured in DMEM/F‐12 medium (Life Technologies) supplemented with 10% FBS and 100 U mL^−1^ of penicillin/streptomycin at 37 °C in a humidified atmosphere with 5% CO_2_. The cells were treated with 1% of O_2_ for 2 h, heatshock (43 °C) for 0.5 h, and 200 µM of sodium arsenite for 1 h to achieve the formation of SGs. Where indicated, cells were treated with 15 mM of sodium lactate or 500 ng mL^−1^ of LPS for 2 h.

### Nuclear and Cytoplasmic Extraction

The extraction of nuclear and cytoplasmic materials was conducted according to the manufacturer's instructions (ThermoFisher Scientific, 78833). Specific methods are described in a previously published work.^[^ ^50^ ^]^

### RNA Interference

siRNAs specific for LDHA‐siRNA, LDHB‐siRNA, TIAR‐siRNA, or HMGB1‐siRNA and scrambled control siRNAs (see Table S1, Supporting Information for siRNA sequences) were purchased from GenePharma. Transfection of siRNAs was conducted using Lipofectamine 3000 (Invitrogen) following the manufacturer's instructions.

### Plasmid Construction

The cDNA of *HMGB1 and TIAR* was amplified from MEF cells and subcloned into the pcDNA3.1 vector (KeyGEN). All cell lines were transiently transfected using Lipofectamine 3000 (Invitrogen) based on the manufacturer's protocols 24 h before other treatments.

### Determination of Lactate Levels

Intracellular lactate levels were determined using a lactate assay kit (A019‐2‐1, Nanjing Jiancheng Bioengineering Institute). Briefly, the cells were lysed in lysis buffer, and the insoluble fraction was removed by centrifugation (12 000 g × 15 min at 4 °C). After 20 µL of supernatant was mixed with 120 µL of reaction mix for 10 min at 37 °C, 200 µL of stop solution was added to terminate the reaction following the manufacturer's instructions. The concentrations of chromogenic products were quantified by measuring absorbance at 570 nm using a TECAN microplate reader. Intracellular lactate levels were normalized to the protein concentration of each sample.

### Immunoprecipitation and Western Blotting

For immunoprecipitation, cells were washed with 1× phosphate‐buffered saline (PBS) and lysed in immunoprecipitation (IP) buffer (Pierce, 26149) containing protease inhibitor cocktail (Roche, 04693132001). The insoluble fraction was removed by centrifugation (12 000 g × 15 min at 4 °C). IP was conducted overnight at 4 °C using anti‐HMGB1, anti‐TIAR, anti‐MYC, anti‐HA, and anti‐FLAG antibodies mixed with Protein A/G Magnetic beads (88802, ThermoFisher Scientific) for 1.5 h at room temperature. The beads were then washed in IP buffer and denatured with SDS loading buffer (SunShineBio, Nanjing, China) before the supernatants were processed for immunoblotting. For western blot analysis, cells from six well plates were washed with PBS, harvested using scrapers, and lysed in RIPA buffer (Beyotime, P0013B) with protease inhibitors. Lysates were centrifuged at 12 000 g for 15 min at 4 °C. Total protein was quantified with a BCA Protein Assay Kit (Beyotime, P0012). Cell samples were heated at 100 °C for 10 min before the protein extracts were separated by electrophoresis on a 4%–20% SurePAGE gel (Genscript, Nanjing, China) and transferred onto polyvinylidene fluoride (PVDF) membranes (Millipore, Bedford, MA, USA). After blocking with 5% bovine serum albumin (BSA) in TBST (Solarbio, Shanghai, China) for 1 h, the membranes were incubated with the appropriate primary antibodies overnight at 4 °C, followed by incubation with the horseradish peroxidase‐conjugated secondary antibody for 2 h at room temperature. The bands were visualized using an ECL HRP Substrate Kit (Advansta), and the signals were quantified using ImageJ software.

### Protein Purification and In Vitro Interaction

To investigate the direct interaction between HMGB1 and TIAR, HIS‐TIAR and GST‐HMGB1 expression vectors were separately transfected into NIH/3T3 cells cultured in 10 cm dishes. The respective proteins were purified using Ni‐Charged MagBeads (Cat. No. L00295, GenScript) and Glutathione MagBeads (Cat. No. L00895, GenScript) following the manufacturer's instructions. For HIS‐TIAR purification, transfected cells were lysed in ice‐cold lysis equilibration buffer (50 mM NaH_2_PO_4_, 300 mM NaCl, pH 7.4), sonicated on ice (180 × 1‐second bursts with 3‐second cooling intervals), and centrifuged at 12 000 × g for 15 min at 4 °C to remove cellular debris. The supernatant was incubated with Ni‐Charged MagBeads at room temperature for 60 min with gentle mixing, followed by three washes with wash buffer (50 mM NaH_2_PO_4_, 300 mM NaCl, 10 mM imidazole, pH 7.4) and three elutions with elution buffer (50 mM NaH_2_PO_4_, 300 mM NaCl, 250 mM imidazole, pH 7.4). For GST‐HMGB1 purification, cell lysates were prepared using a French press or sonication, incubated with pre‐washed Glutathione MagBeads for 120 min, and washed three times with Binding/Wash Buffer. Proteins were eluted by incubating the beads with 100 µL of Elution Buffer at room temperature for 5 min, and the elution step was repeated twice to maximize recovery. All purified proteins were quantified and stored at −80 °C for further experiments. To assess the direct interaction between HIS‐TIAR and GST‐HMGB1, 500 µL of each purified protein was mixed and incubated at room temperature for 12 h, followed by immunoprecipitation with an anti‐HIS antibody to detect their interaction.

### Immunofluorescent Staining

For immunofluorescence staining of ovarian tissue sections, mice ovaries (n = 5 in each group) obtained as previously described^[^ ^51^ ^]^ were embedded in paraffin, continuously sectioned to 5 µm, and mounted on glass slides. The sections near the middle of the ovaries (of maximum diameter) were used for histological examination. Briefly, the slides were deparaffinized in xylene, rehydrated, and retrieved by heating with citrate buffer (10 mM of sodium citrate, 0.05% Tween‐20, pH 6.0) for 0.5 h. Endogenous peroxidase activity was eliminated by incubation with 3% H_2_O_2_ for 10 min. After blocking with 2% BSA for 1 h, the slides were incubated with antibodies against TIAR (1:50) overnight at 4 °C, followed by incubation with rhodamine (TRITC)‐conjugated goat anti‐rabbit IgG (1:100) for 2 h at room temperature. The nuclei were stained with DAPI for 6 min before images were acquired using a Zeiss LSM 900 confocal microscope.

For immunofluorescence staining of cultured cells before treatment, cells were seeded on glass coverslips. After the cells were fixed with 4% paraformaldehyde (Sigma–Aldrich, P‐6148) for 30 min, the samples were washed three times with PBS for 5 min each time and permeabilized with 0.5% Triton‐X‐100 (Sigma–Aldrich, T8787) for 10 min at room temperature. The samples were then incubated with blocking buffer (2% BSA in PBS) (Sigma–Aldrich, A3059) before being processed for incubation with primary antibodies overnight at 4 °C. Following three rinses with PBS, the coverslips were incubated with secondary antibodies conjugated to Alexa Fluor 488 or rhodamine for 2 h. The cell samples were then washed three times with PBS and stained with DAPI (Sigma–Aldrich, D8417) before images were acquired using a Zeiss LSM 900 confocal microscope.

### Ultra High‐Performance Liquid Chromatography‐Mass Spectrometry Analysis

After Coomassie Brilliant Blue staining, corresponding gel bands were excised with a razor blade, cut into ≈1 mm^3^ pieces, and transferred into Eppendorf (EP) tubes filled with deionized water. The samples were lysed with a four‐fold volume of urea lysis buffer, followed by sonication and centrifugation. The resulting supernatant was used for protein concentration determination and subsequent digestion with trypsin. After digestion, peptides were subjected to acetone precipitation, followed by reduction with DTT and alkylation with IAA. Subsequently, the tryptic peptides were dissolved in solution A and directly loaded onto a self‐made reversed‐phase analytical column (25 cm in length, 100 µm in diameter). The mobile phase consisted of solution A (0.1% formic acid, 2% acetonitrile/in water) and solution B (0.1% formic acid, 90% acetonitrile/in water). Peptides were then separated with the gradient according to the following specifications, all conducted at a constant flow rate of 500 nl min^−1^ on a Vanquish Neo UHPLC System (ThermoFisher Scientific): 0–68 min, 6%–23% B; 68–82 min, 23%–32% B; 82–86 min, 32%–80% B; 86–90 min, 80% B.

The obtained peptides were analyzed using an Orbitrap Exploris 480 with a nano‐electrospray ion source. The electrospray voltage was 2300 V, and the FAIMS compensate voltage (CV) was set at –45 and –65 V. Precursors and fragments were analyzed using the Orbitrap detector. The full mass spectrometry (MS) scanning resolution was set at 60 000 for a scanning range of 400–1200 m z^−1^. The MS/MS scanning rate was fixed first as 110 m z^−1^ at a resolution of 15 000 with the TurboTMT set as off. Up to 25 of the most abundant precursors were then chosen for MS/MS analyses with a 20‐s dynamic exclusion. The higher energy collisional dissociation (HCD) fragmentation was conducted at a normalized collision energy (NCE) of 27%. The automatic gain control (AGC) target was set at 100%, with an intensity threshold of 50 000 ions/s and a maximum injection time of Auto. The MS/MS data were processed using PD search engine (v.2.4). Trypsin/P was specified as the cleavage enzyme, allowing up to four missing cleavages. The mass tolerance for precursor ions was set as 10 ppm in the first search, and the mass tolerance for fragment ions was set as 0.02 Da. Carbamidomethyl on Cys was specified as a fixed modification, and lactylation was specified as a variable modification. The false discovery rate (FDR) was adjusted to <1%. The minimum peptide ion scoring requirement was set above 0, with the identification confidence set as low.

### Analysis of Identity of HMGB1 Protein in Different Species

HMGB1 protein sequences of humans, mice, and pigs were obtained from the National Center for Biotechnology Information (NCBI; https://www.ncbi.nlm.nih.gov/). Protein sequence alignment was conducted using DNAMAN software.

### Structural Analysis of HMGB1 Protein

Alphafold2 software was applied to predict the protein structure of wild‐type (WT), K177R, and K172R mutant HMGB1 from amino acid sequences (NP_001300823.1).

### Protein‐Protein Docking

The protein‐protein docking study was conducted by performing the following steps. 1) Structure acquisition: Use Alphafold2 software to perform structure prediction based on the sequence, and obtain the protein structure protein data bank (PDB) file. 2) Molecular docking: Use ClusPro software (version 2.0 https://cluspro.bu.edu/) to perform molecular docking of HMGB1 and TIAR protein structures. 3) Visualizing the results: Select the docking energy top 5 pose, which was the binding pose showing the top 5 binding affinities of the two proteins. Visualize the docking results using Pymol software, and annotate the key amino acid residues of the docking located on the docking interaction surface.

### Statistical Analysis

Statistical significance was determined using SPSS version 20.0. software. Data are expressed as the mean ± standard error. All experiments were performed in triplicate and repeated three times. Differences between two groups were determined using the Student *t*‐test. Comparisons involving more than two groups were analyzed using one‐way analysis of variance (ANOVA), followed by the least significant differences (LSD) post hoc test. P values > 0.05 were considered statistically significant (NS), P values < 0.05 indicate significant (*P < 0.05), and P values < 0.01 or 0.001 indicate highly significant (**P < 0.01 or *** P < 0.001).

## Conflict of Interest

The authors declare no conflict of interest.

## Author Contributions

C.L. and Z.L. contributed equally to this work. C.L. and Z.L. as co‐first authors. M.S., G.W., C.L., and H.L. performed conceptualization; H.L., C.L., and M.S. performed experiment design; C.L., M.S., and H.L. performed validation; C.L., Z.L., L.Z., G.W., C.F., H.L., T.H., and M.S. performed investigation; C.L. and Z.L. performed data analysis and interpretation; C.L. wrote the original draft; M.S. and C.L. wrote and reviewed; M.S. and H.L. performed supervision; M.S., C.L., and H.L. performed project administration; C.L., M.S., and H.L. performed funding acquisition; Final approval of manuscript from all authors.

## Supporting information



Supporting Information

Supporting Information

## Data Availability

The data that support the findings of this study are available from the corresponding author upon reasonable request.
