# Supplementary material for: Lactylation of HMGB1 at K177 Drives Nuclear Export of TIAR to Promote Hypoxia‐Induced Stress Granule Formation
Source: Adv Sci (Weinh). 2025 Aug 11;12(41):e04896. doi: 10.1002/advs.202504896 (PMC12591107; doi:10.1002/advs.202504896)
Supplement: Supplementary file 1 — Supporting Information [file ADVS-12-e04896-s001.doc]

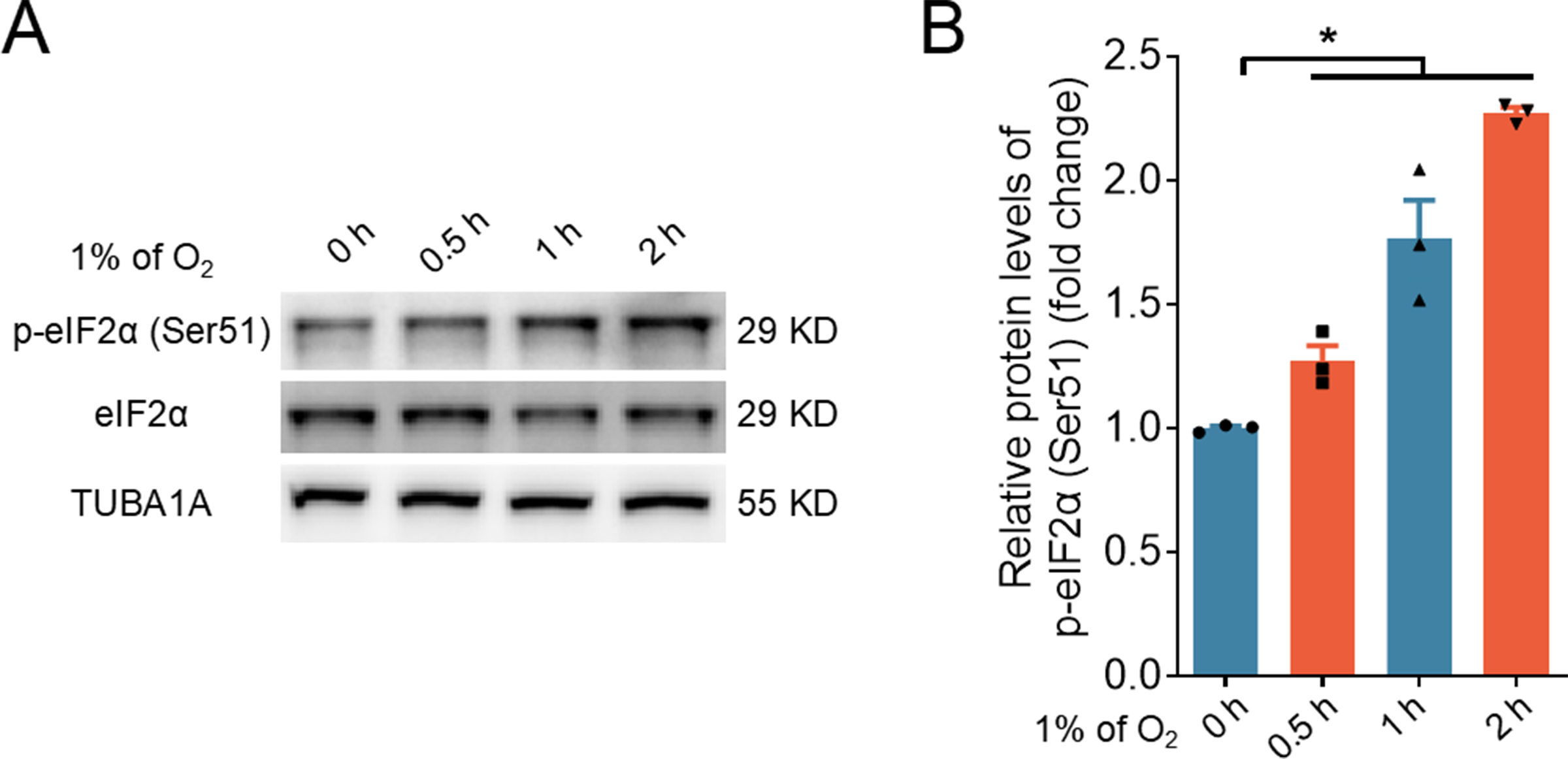


**Figure S1.** Hypoxia promotes the phosphorylation of eIF2α at Ser51. (A, B) NIH/3T3 cells were cultured under hypoxic conditions for 0 h, 0.5 h, 1 h, or 2 h, and Western blot analysis was performed to examine the protein levels of p-eIF2α (Ser51), total eIF2α, and TUBA1A (A). Band intensities were quantified (B). Data are presented as mean ± s.e.m. (n = 3). **P* < 0.05.


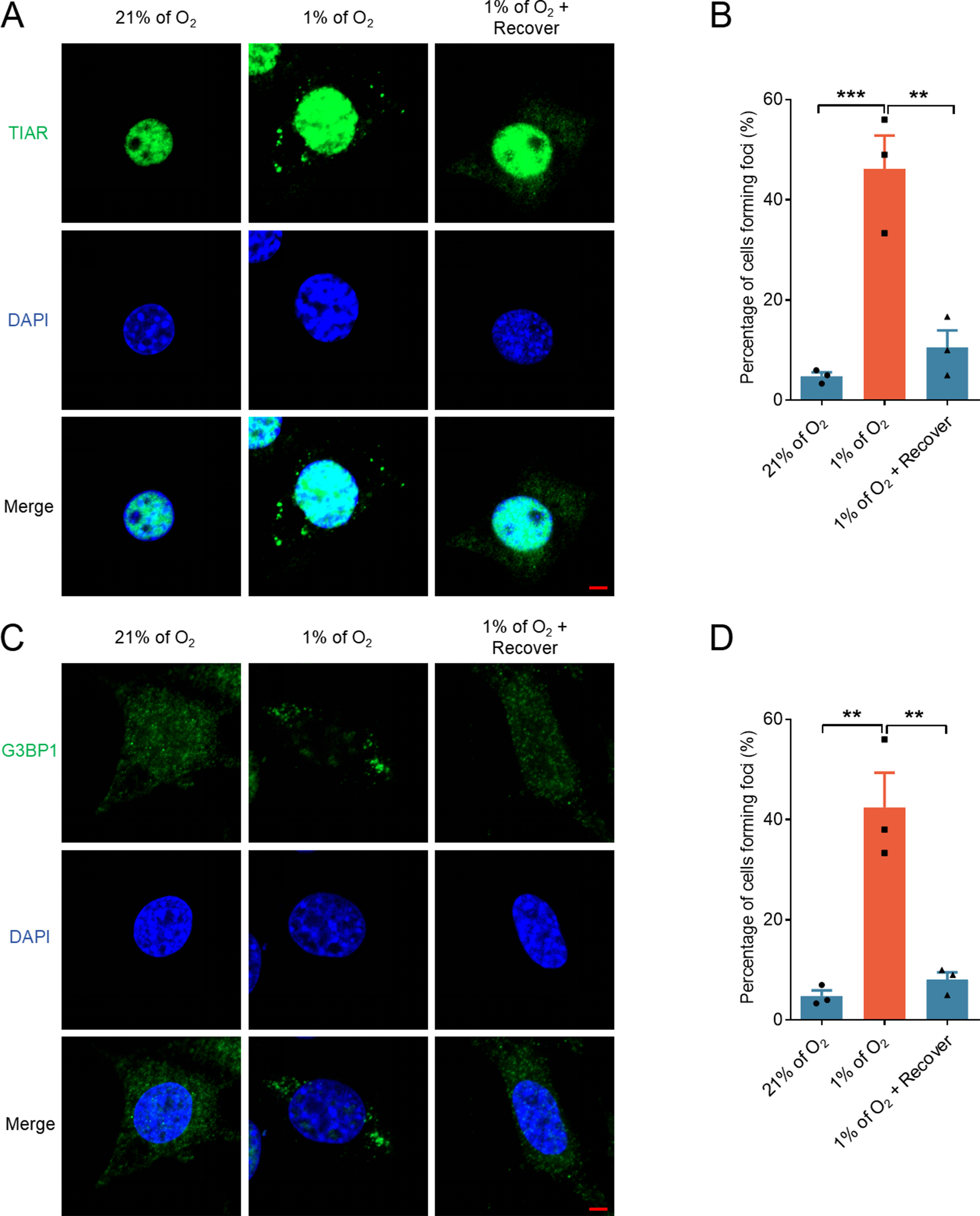


**Figure S2.** Hypoxia-induced SG formation is reversible under normoxic conditions. (A-D) NIH/3T3 cells were exposed to hypoxia for 2 h, then returned to normoxia, and the subcellular localization of TIAR (A) and G3BP1 (C) was examined by immunofluorescence. The proportion of cells containing TIAR-positive (B) and G3BP1-positive (D) foci was quantified using laser confocal microscopy. Data are presented as mean ± s.e.m. (n = 3). ***P* < 0.01, ****P* < 0.001.


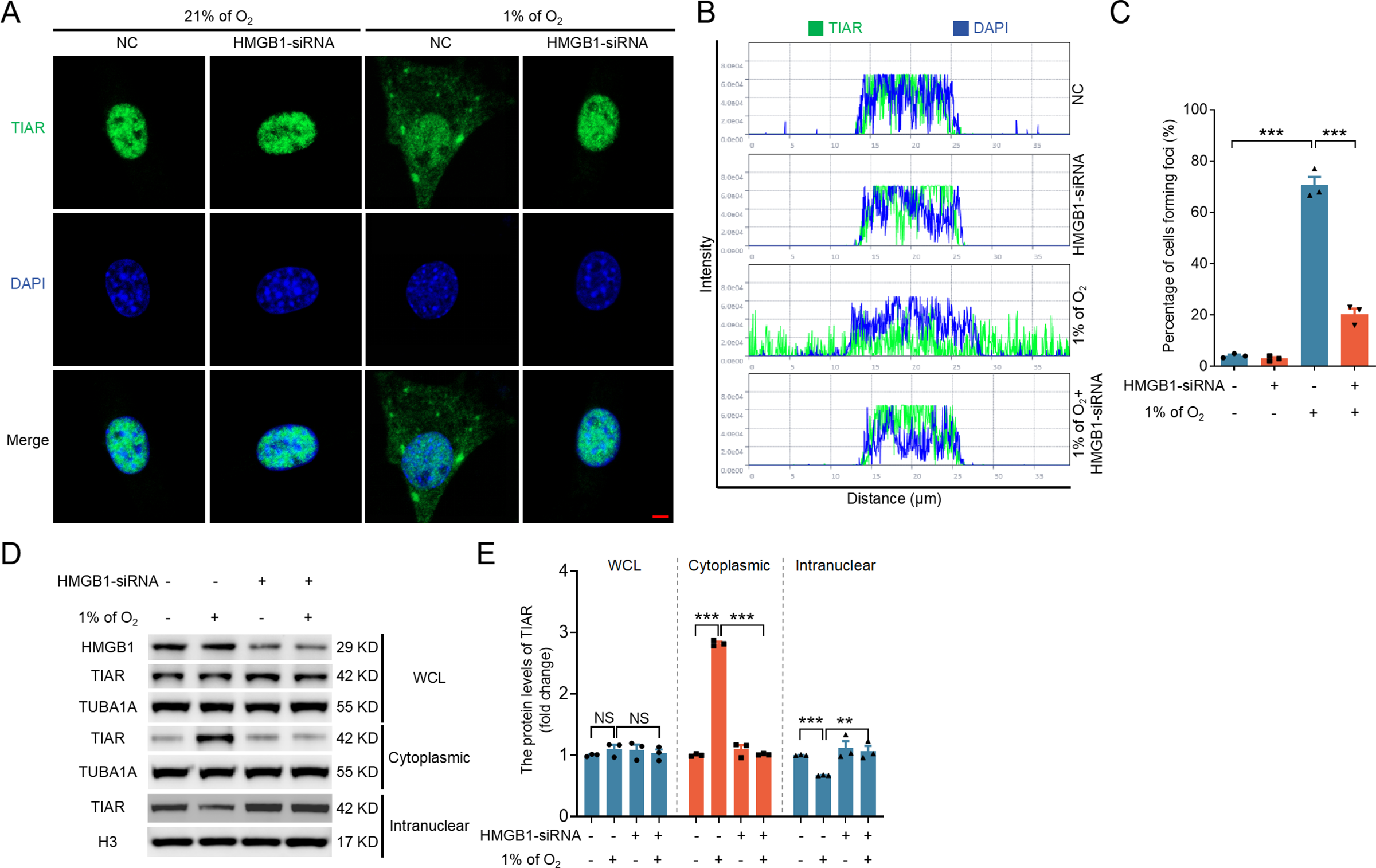


**Figure S3.** HMGB1 regulates the nuclear export of TIAR for the formation of SGs under hypoxia in MEF cells. A–E, MEF cells transfected with HMGB1 siRNA or scramble control siRNA for 24 h were cultured for 2 h under normoxic (21% of O_2_) or hypoxic (1% of O_2_) conditions. Cells were then harvested for immunofluorescence assay to observe the subcellular localization of TIAR (A). The fluorescence intensity curve shows the distribution of TIAR (green) and DAPI (blue) along cells (B). Scale bar = 5 μm. The proportion of cells containing TIAR foci was counted by laser confocal microscopy (C). Cells were collected for measuring TIAR expression via western blot analysis of cytoplasmic and nuclear fractions (D), and the data were quantified (E). Data are presented as mean ± s.e.m. (n = 3). ***P* < 0.01, ****P* < 0.001; NS, not significant (*P* > 0.05).


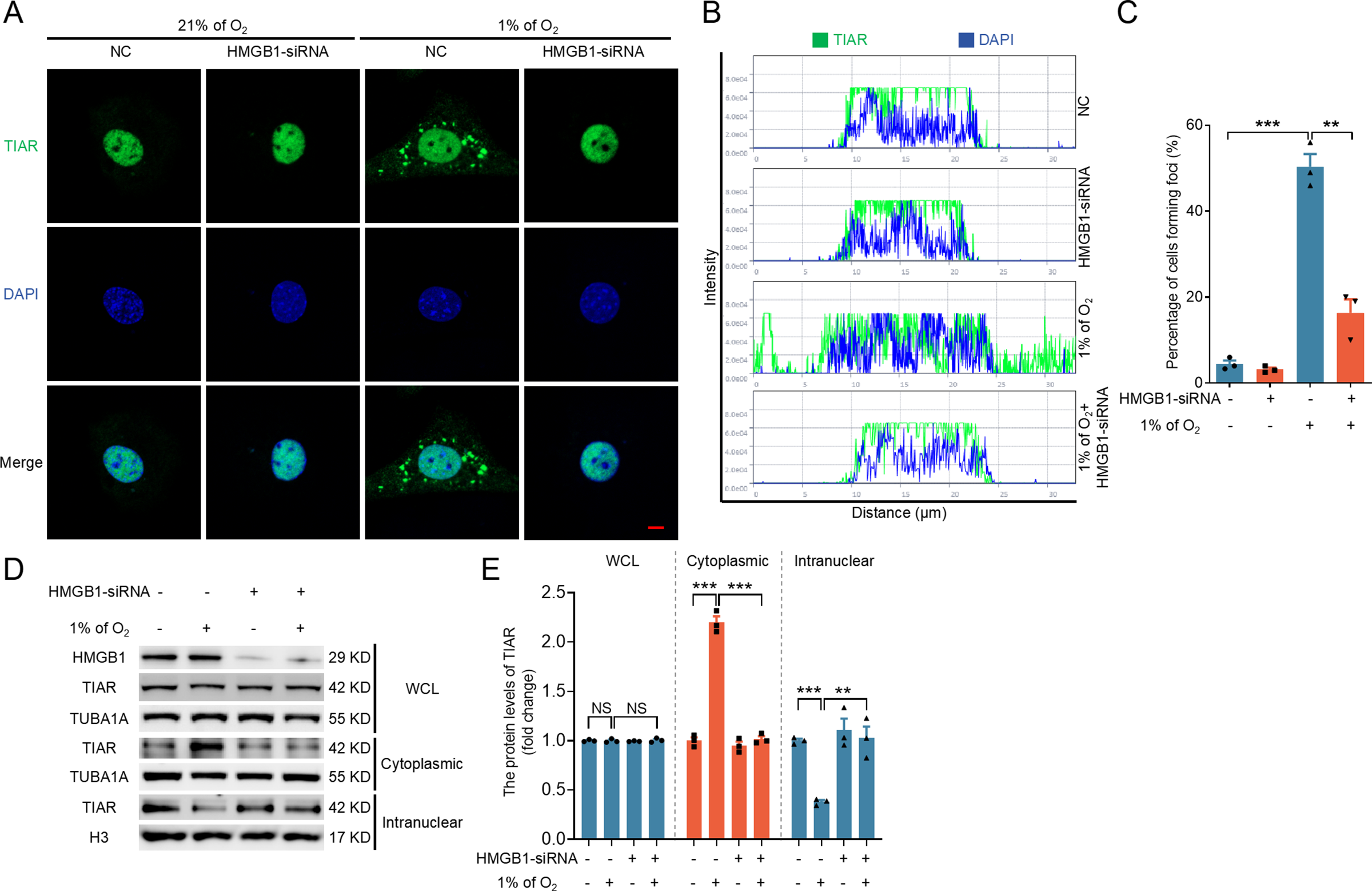


**Figure S4.** HMGB1 regulates the nuclear export of TIAR for the formation of SGs under hypoxia in murine GCs. A–E, Cells transfected with HMGB1 siRNA or scramble control siRNA for 24 h were cultured for 2 h under normoxic (21% of O_2_) or hypoxic (1% of O_2_) conditions. Cells were then harvested for immunofluorescence assay to observe the subcellular localization of TIAR (A). The fluorescence intensity curve shows the distribution of TIAR (green) and DAPI (blue) along the cells (B). Scale bar = 5 μm. The proportion of cells containing TIAR foci was counted by laser confocal microscopy (C). Cells were collected for measuring TIAR expression via western blot analysis of cytoplasmic and nuclear fractions (D), and the data were quantified (E). Data are presented as mean ± s.e.m. (n = 3). ***P* < 0.01, ****P* < 0.001; NS, not significant (*P* > 0.05).


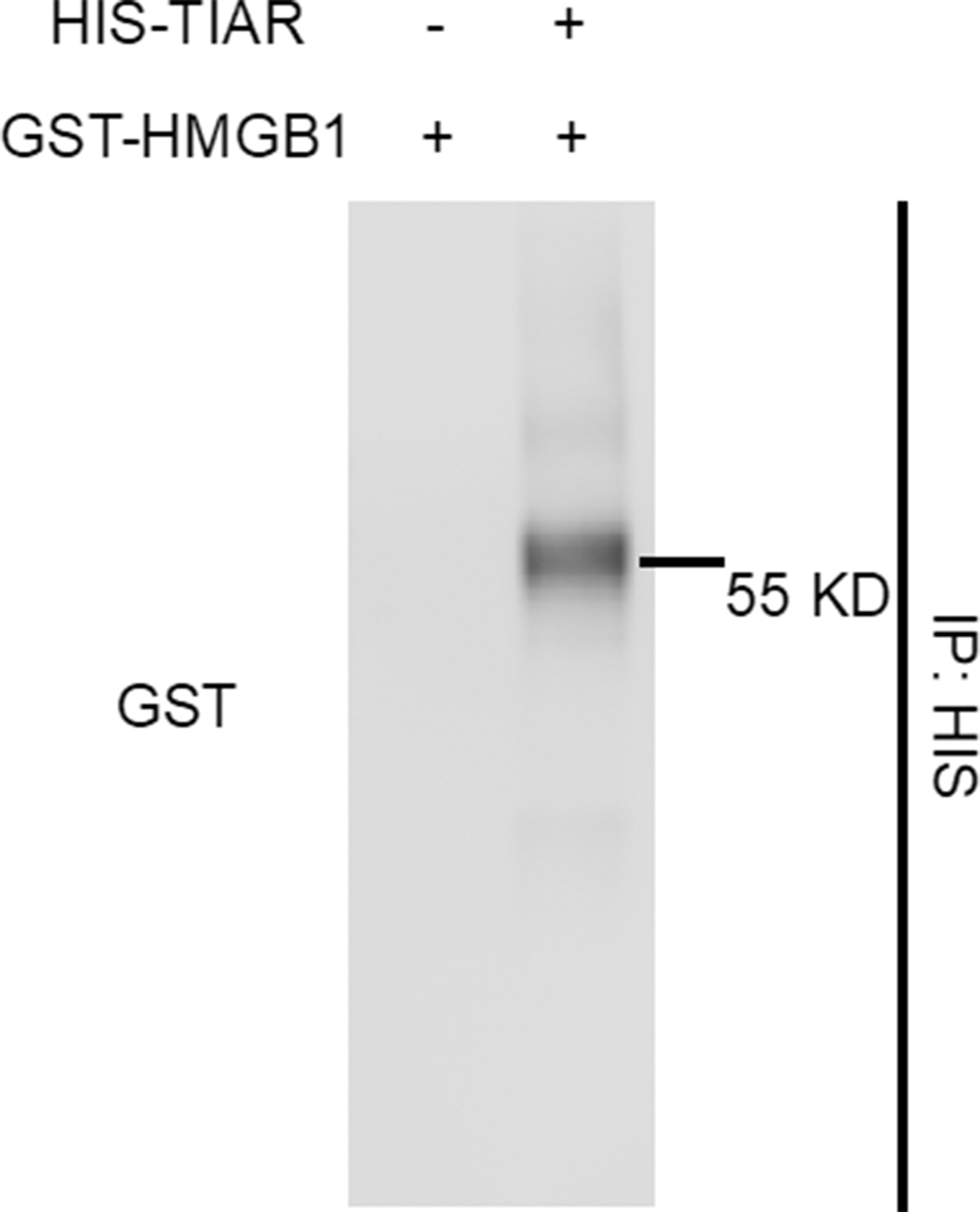


**Figure S5.** Direct interaction between HMGB1 and TIAR. HIS-TIAR and GST-HMGB1 expression vectors were individually transfected into NIH/3T3 cells, and the respective proteins were purified. After in vitro incubation, immunoprecipitation was performed using an anti-HIS antibody, followed by Western blot analysis to detect GST. The experiment was performed in three independent biological replicates.


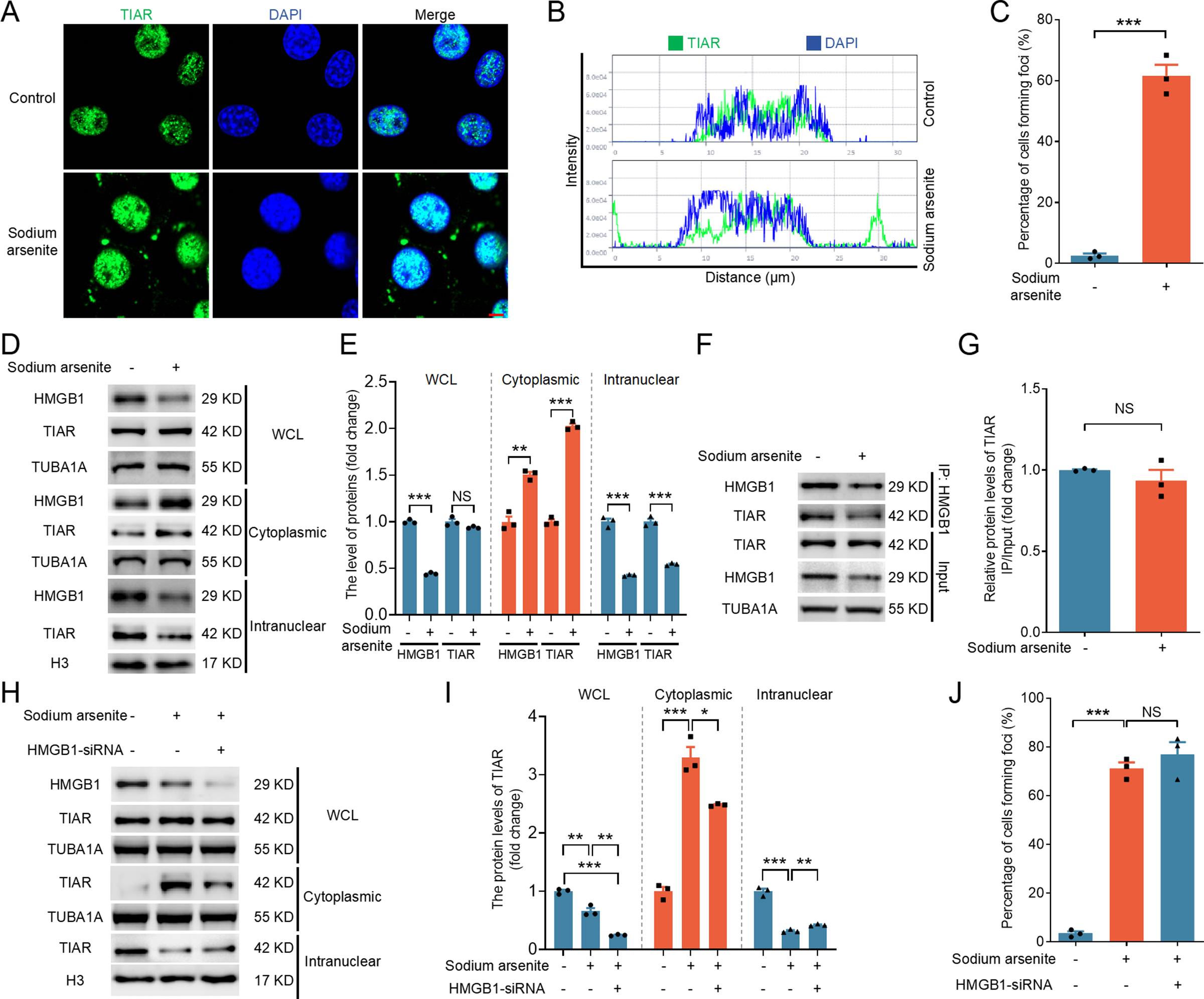


**Figure S6.** Sodium arsenite promotes TIAR nuclear export for the formation of SGs independent of HMGB1 nuclear export. A–G, NIH/3T3 cells were cultured with or without sodium arsenite (200 μM) for 2 h and then harvested to observe the subcellular localization of TIAR by immunofluorescence assay (A). The fluorescence intensity curve shows the distribution of TIAR (green) and DAPI (blue) along the cells (B). Scale bar = 5 μm. The proportion of cells that contain foci formed by TIAR was counted by laser confocal microscopy (C). D–G, Cells were collected for immunoblotting analysis of the protein level of HMGB1 or the protein level of TIAR after nuclear and cytoplasmic extraction (D) and quantified (E). IP assay was conducted to analyze the interaction of HMGB1 with TIAR (F), and the bands were quantified (G). H–J, Cells transfected with HMGB1 siRNA or scramble control siRNA for 24 h were cultured with or without sodium arsenite (200 μM) for 2 h. Cells were collected for measuring TIAR expression via western blot analysis of cytoplasmic and nuclear fractions (H), the data were quantified (I), and the proportion of cells that contain foci formed by TIAR was counted by laser confocal microscopy (J). Data are presented as mean ± s.e.m. (n = 3). **P* < 0.05, ***P* < 0.01, ****P* < 0.001; NS, not significant (*P* > 0.05).


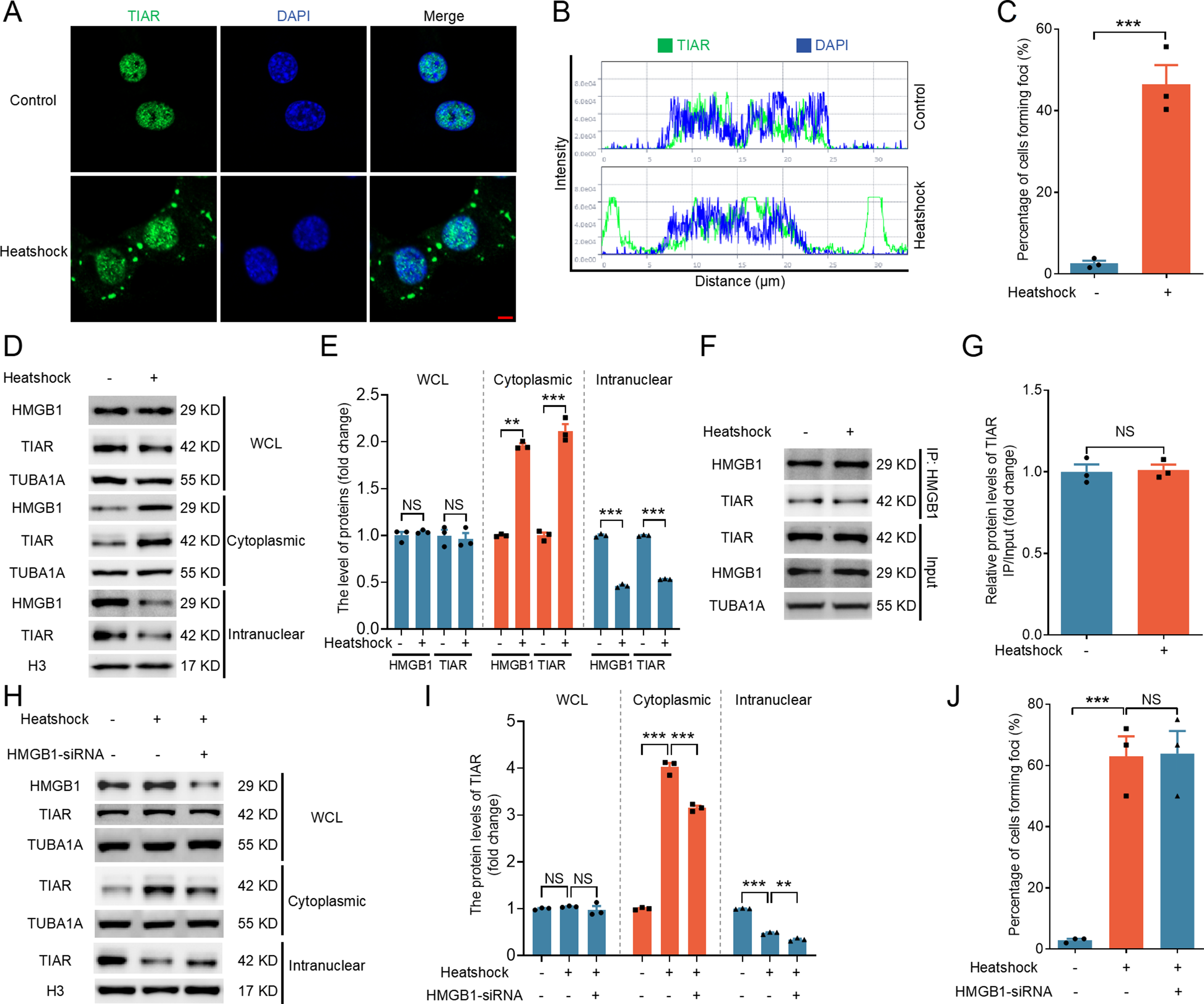


**Figure S7.** Heatshock promotes TIAR nuclear export for the formation of SGs independent of HMGB1 nuclear export. A–G, NIH/3T3 cells were exposed or not exposed to a 43°C water bath for 0.5 h and then harvested to observe the subcellular localization of TIAR by immunofluorescence assay (A). The fluorescence intensity curve shows the distribution of TIAR (green) and DAPI (blue) along the cells (B). Scale bar = 5 μm. The proportion of cells that contain foci formed by TIAR was counted by laser confocal microscopy (C). D–G, Cells were collected for immunoblotting analysis of the protein level of HMGB1 or the protein level of TIAR after nuclear and cytoplasmic extraction (D) and quantified (E). IP assay was conducted to analyze the interaction of HMGB1 with TIAR (F), and the bands were quantified (G). H–J, Cells transfected with HMGB1 siRNA or scramble control siRNA for 24 h were cultured with or without sodium arsenite (200 μM) for 2 h. Cells were collected for measuring TIAR expression via western blot analysis of cytoplasmic and nuclear fractions (H), the data were quantified (I), and the proportion of cells that contain foci formed by TIAR was counted by laser confocal microscopy (J). Data are presented as mean ± s.e.m. (n = 3). ***P* < 0.01, ****P* < 0.001; NS, not significant (*P* > 0.05).


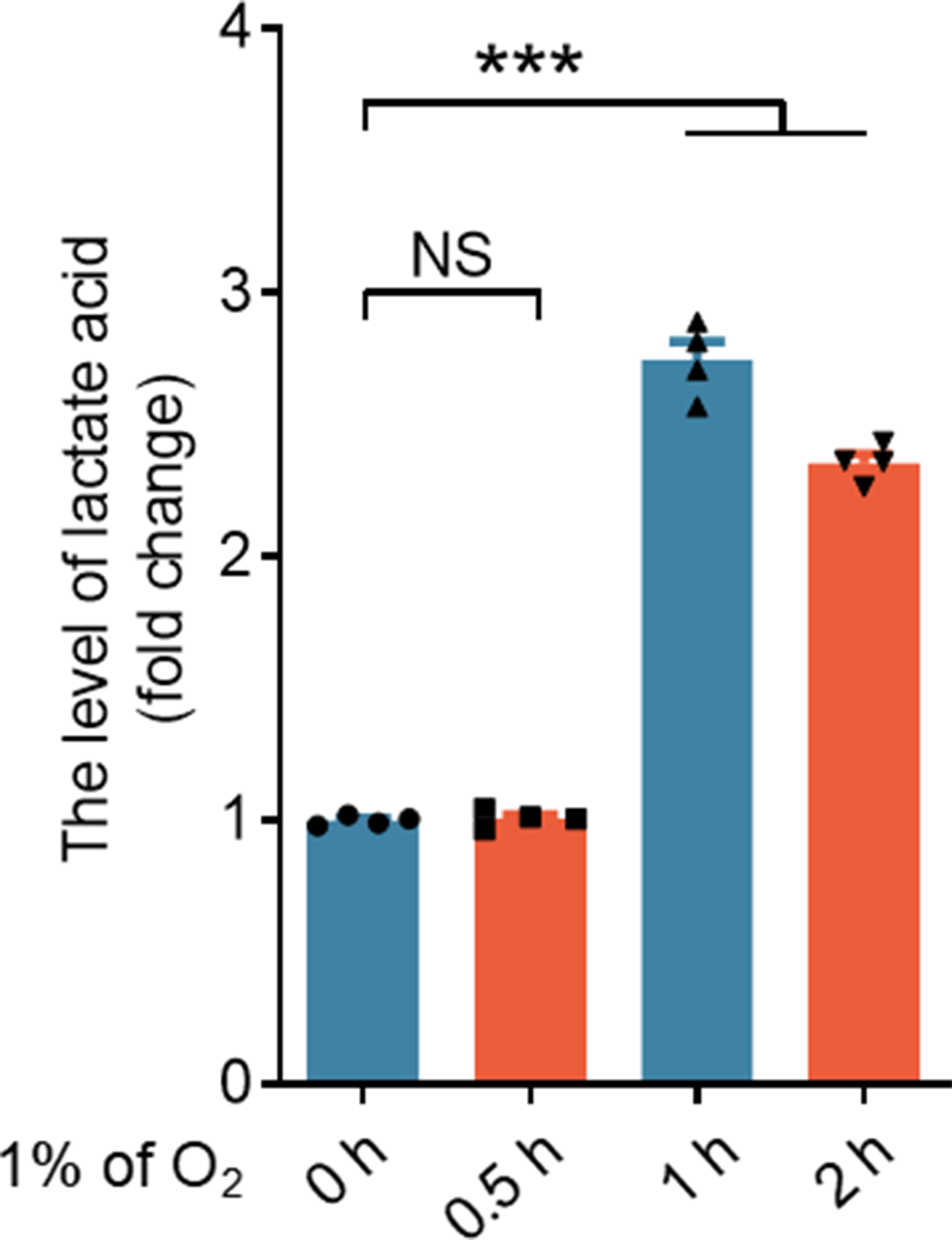


**Figure S8.** Hypoxia promotes lactate production. NIH/3T3 cells were cultured under hypoxic conditions for 0 h, 0.5 h, 1 h, or 2 h, followed by measurement of lactate levels. Data are presented as mean ± s.e.m. (n = 4). ****P* < 0.001; NS, not significant (*P* > 0.05).


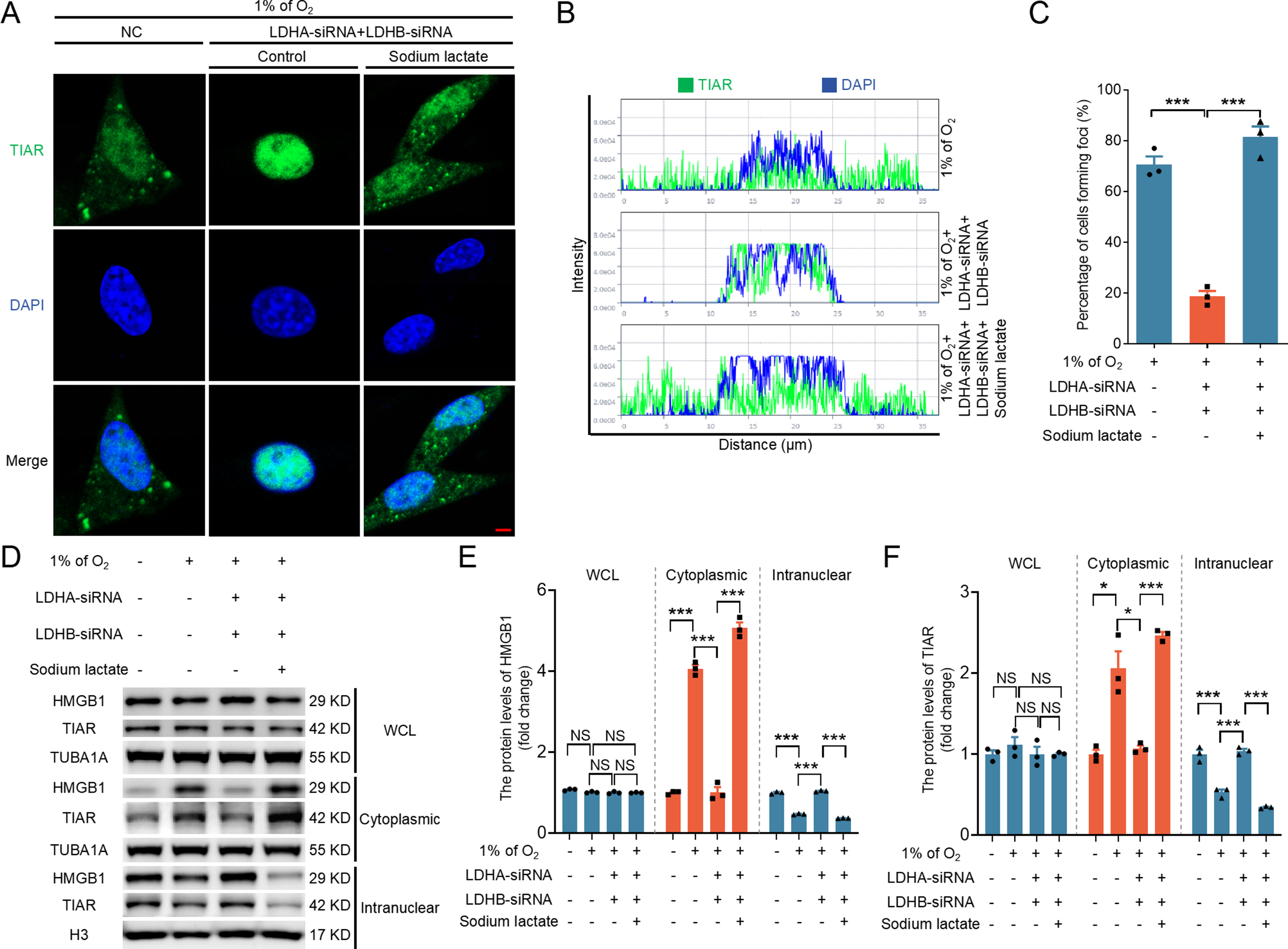


**Figure S9.** Hypoxia promotion of SG formation and nuclear export of HMGB1 or TIAR are dependent on lactate production in MEF cells. A–F, Cells transfected with LDHA/LDHB siRNAs or scramble control siRNA for 24 h were treated with or without 1 mM of sodium lactate and cultured under hypoxia (1% of O_2_) for an additional 2 h. The subcellular localization of HMGB1 and TIAR was observed by immunofluorescence assay (A). The fluorescence intensity curve shows the distribution of TIAR (green) and DAPI (blue) along the cells (B). Scale bar = 5 μm. The proportion of cells that contain foci formed by TIAR was counted by laser confocal microscopy (C). The cytoplasmic and nuclear fractions of cells were analyzed for detecting the protein levels of HMGB1 and TIAR by immunoblotting (D). The bands were quantified (E and F). Data are presented as mean ± s.e.m. (n = 3). **P* < 0.05, ****P* < 0.001; NS, not significant (*P* > 0.05).


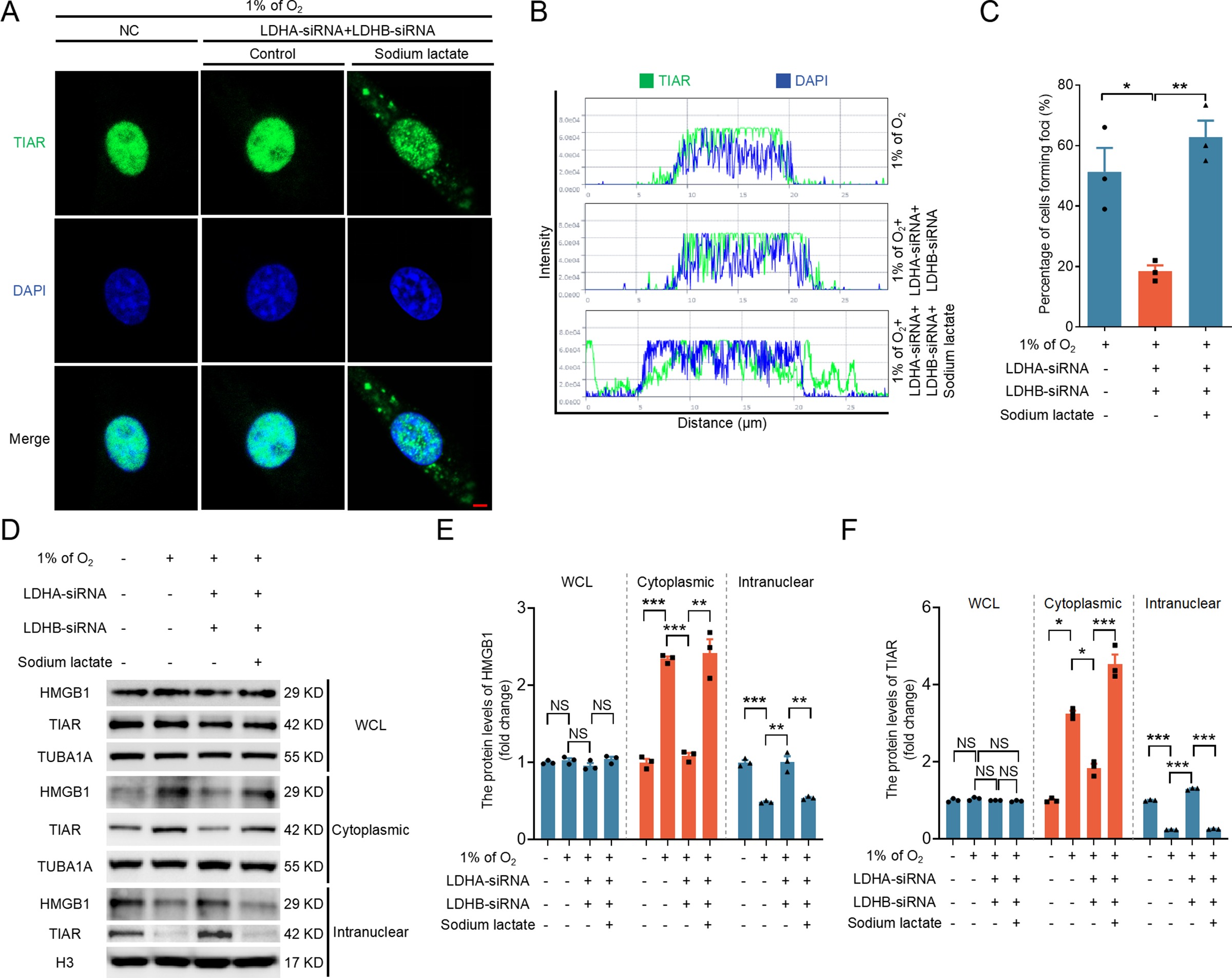


**Figure S10.** Hypoxia promotion of SG formation and nuclear export of HMGB1 or TIAR are dependent on lactate production in murine GCs. A–F, Cells transfected with LDHA/LDHB siRNAs or scramble control siRNA for 24 h were treated with or without 1 mM of sodium lactate and cultured under hypoxia (1% of O_2_) for an additional 2 h. The subcellular localization of HMGB1 and TIAR was observed by immunofluorescence assay (A). The fluorescence intensity curve shows the distribution of TIAR (green) and DAPI (blue) along the cells (B). Scale bar = 5 μm. The proportion of cells that contain foci formed by TIAR was counted by laser confocal microscopy (C). The cytoplasmic and nuclear fractions of cells were analyzed for detecting the protein levels of HMGB1 and TIAR by immunoblotting (D). The bands were quantified (E and F). Data are presented as mean ± s.e.m. (n = 3). **P* < 0.05, ***P* < 0.01, ****P* < 0.001; NS, not significant (*P* > 0.05).


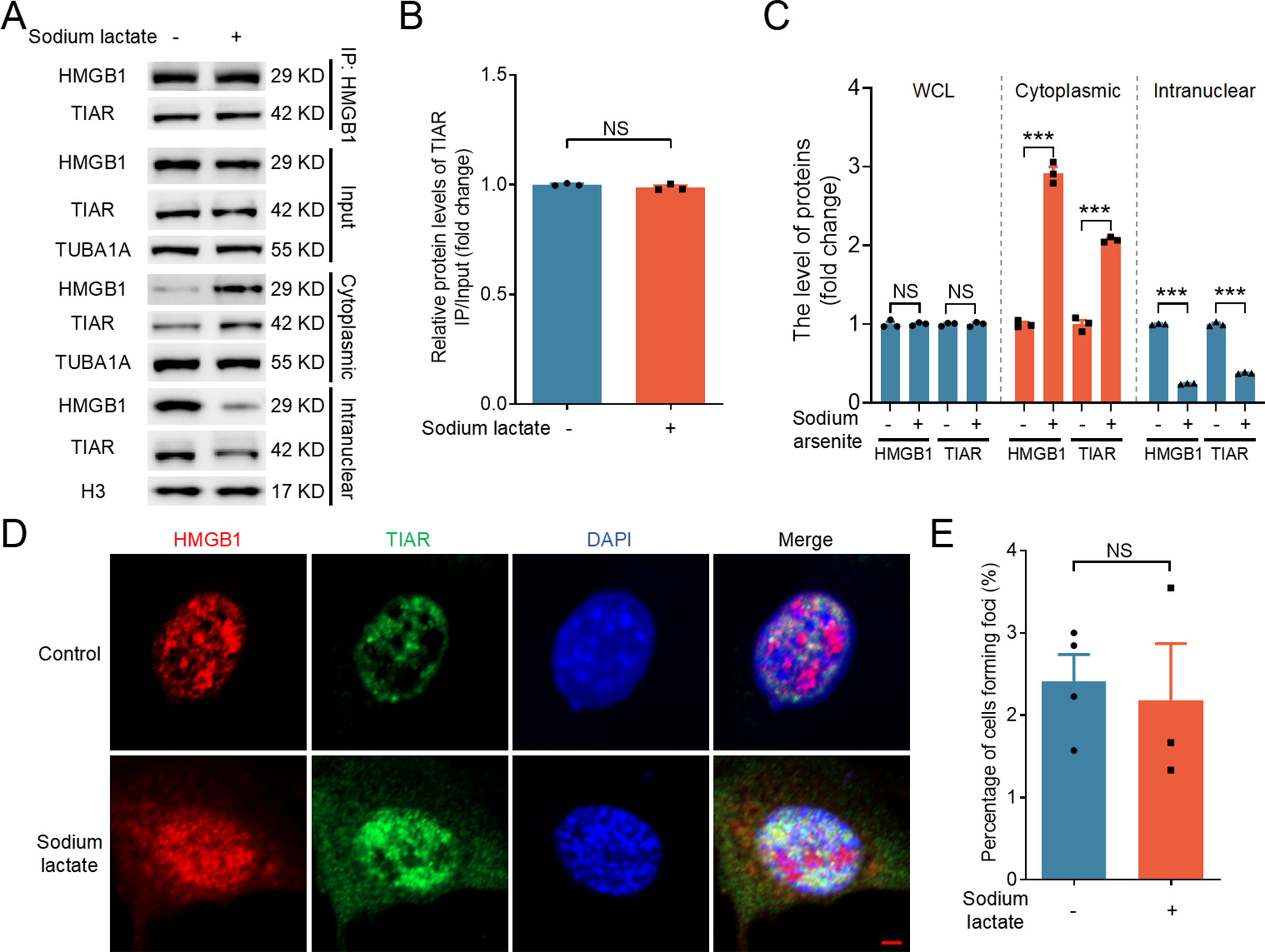


**Figure S11.** Sodium lactate promotes the nuclear export of HMGB1 and TIAR but does not induce SG formation. A–E, NIH/3T3 cells were cultured under normoxic (21% of O_2_) or hypoxic (1% of O_2_) conditions for 2 h. Immunoprecipitation (IP) was performed to assess the interaction between HMGB1 and TIAR (A). Nuclear and cytoplasmic fractions were subjected to immunoblotting to examine the protein levels of HMGB1 and TIAR (A), and band intensities were quantified (B and C). Immunofluorescence staining was conducted to analyze the subcellular distribution of HMGB1 and TIAR (D). The proportion of cells containing TIAR foci was quantified using confocal microscopy (E). Data are presented as mean ± s.e.m. (n = 3). ****P* < 0.001; NS, not significant (*P* > 0.05).


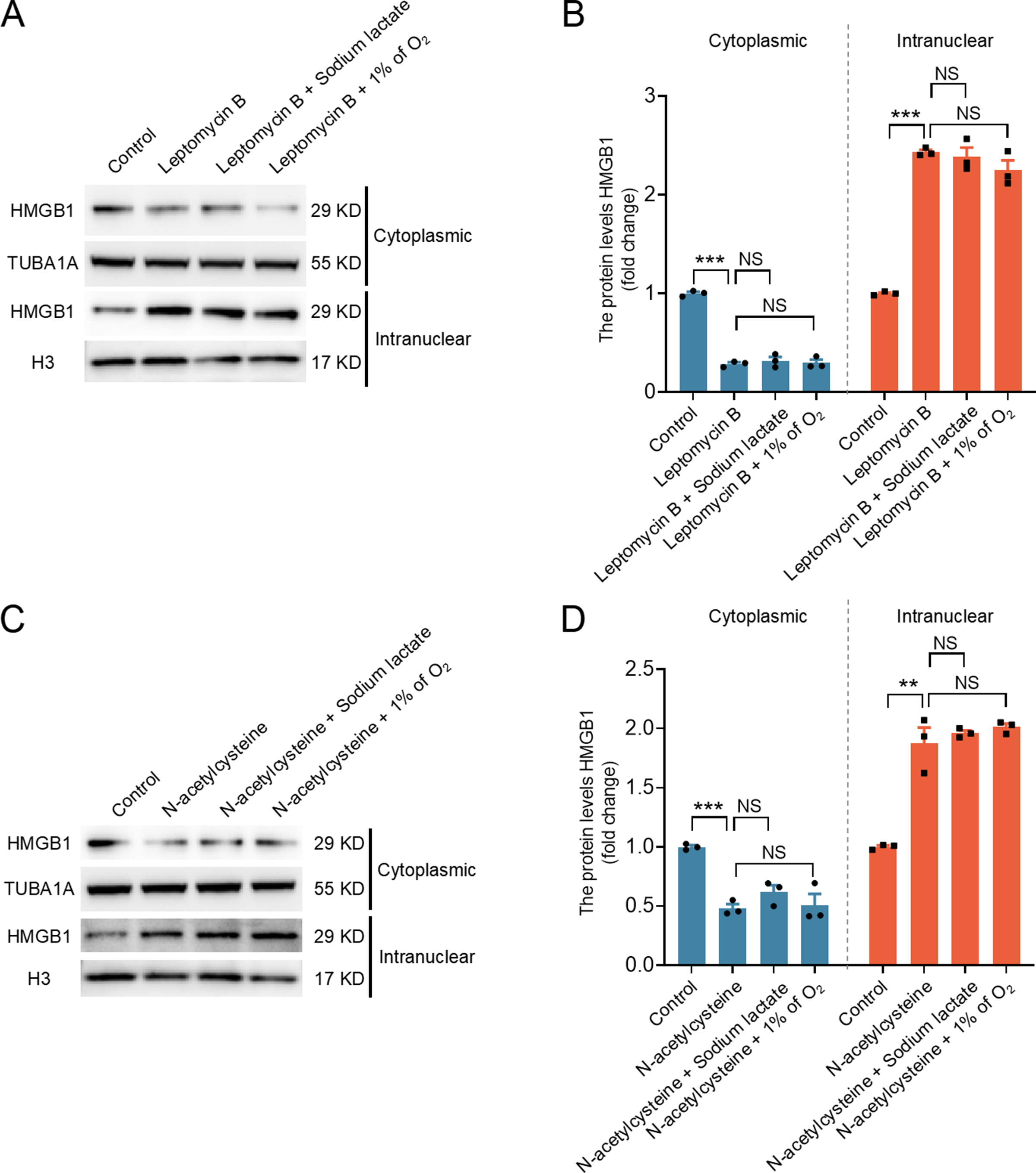


**Figure S12.** Hypoxia or sodium lactate promotes HMGB1 nuclear export. (A-D) NIH/3T3 cells were pretreated with 10 nM Leptomycin B (A and B) or 2 mM N-acetylcysteine (C and D) for 2 h, followed by incubation under hypoxic conditions or with 1 mM sodium lactate for an additional 2 h. Cells were then harvested for nuclear-cytoplasmic fractionation, and the distribution of HMGB1 was analyzed by Western blot (A and C). Band intensities were quantified (B and D). Data are presented as mean ± s.e.m. (n = 3). ***P* < 0.01, ****P* < 0.001; NS, not significant (*P* > 0.05).


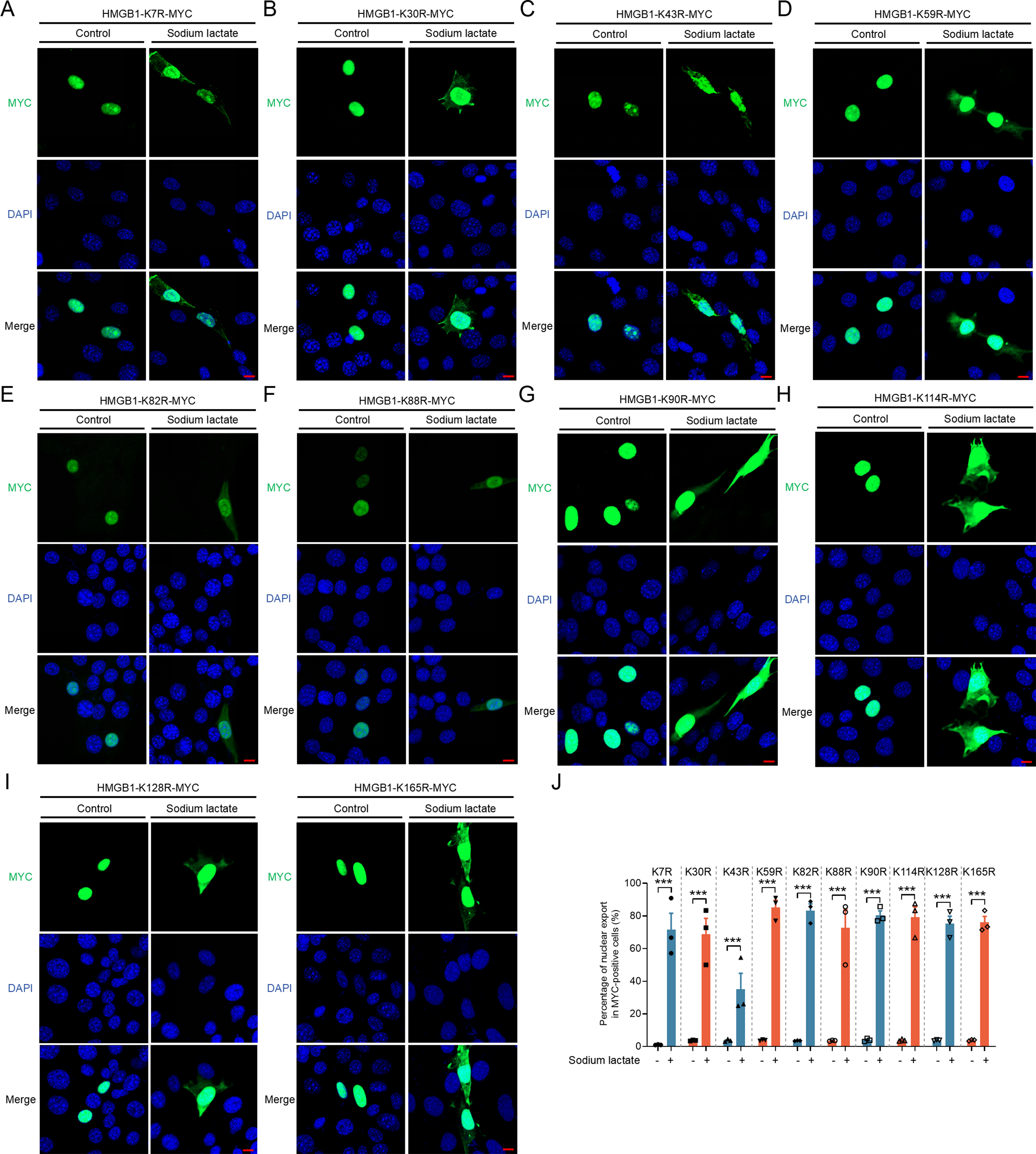


**Figure S13.** Hypoxia promotes the nuclear export of HMGB1, which includes the mutation of lysine to arginine. A–J, NIH/3T3 cells overexpressing vectors encoding MYC-tagged mutant HMGB1 including K7R, K30R, K43R, K59R, K82R, K88R, K90R, K114R, K128R, and K165R for 24 h were cultured with or without 1mM of sodium lactate for a further 2 h. Cells were harvested to detect the subcellular localization of MYC by immunofluorescence assay (A–I), and the proportion of cells with cytoplasmic localization of MYC was counted by laser confocal microscopy (J). Data are presented as mean ± s.e.m. (n = 3). ****P* < 0.001; NS, not significant (*P* > 0.05).


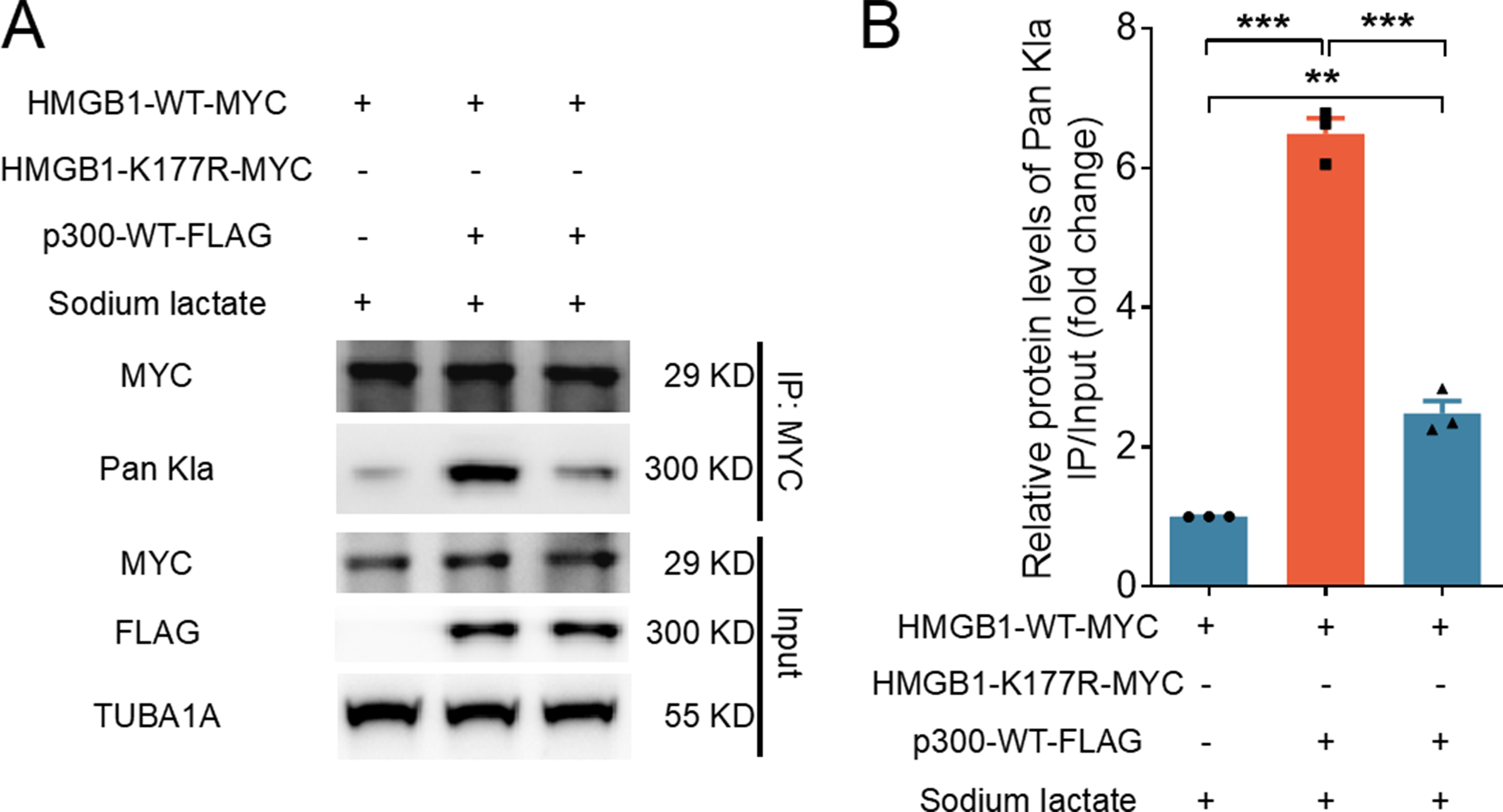


**Figure S14.** p300 promotes HMGB1 lactylation. A and B, NIH/3T3 cells were transfected with or without p300 along with either wild-type HMGB1 or the K177R mutant, followed by treatment with 1 mM sodium lactate for 2 h. Immunoprecipitation (IP) was performed to assess HMGB1 lactylation levels (A), and band intensities were quantified (B). Data are presented as mean ± s.e.m. (n = 3). ***P* < 0.01, ****P* < 0.001.


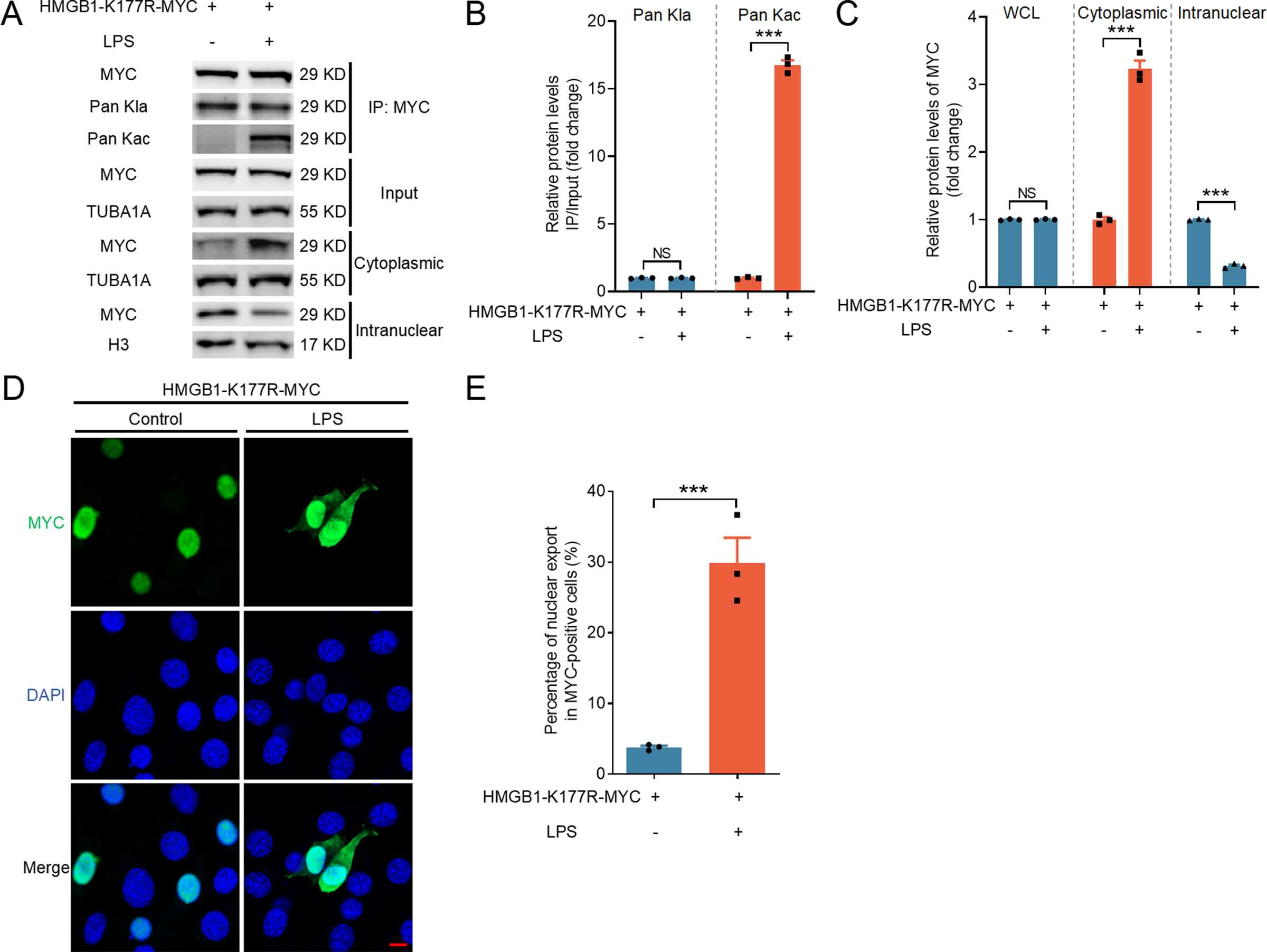


**Figure S15.** Lipopolysaccharide (LPS) promotes the nuclear export of HMGB1-K177R-MYC and is not dependent on the lactylation of HMGB1. A–E, NIH/3T3 cells transfected with HMGB1-K177R-MYC vectors for 24 h were treated with or without LPS (500 ng/ml) for 2 h. Cells were harvested to separate nuclear and cytoplasmic fractions, the protein levels of MYC were analyzed using immunoblotting, and IP analysis of the lactylation and acetylation levels of MYC protein was performed (A). The protein bands were quantified (B and C). The subcellular localization of MYC was detected by immunofluorescence assay (D), and the proportion of cells with cytoplasmic localization of MYC was counted by laser confocal microscopy (E). Data are presented as mean ± s.e.m. (n = 3). ****P* < 0.001; NS, not significant (*P* > 0.05).


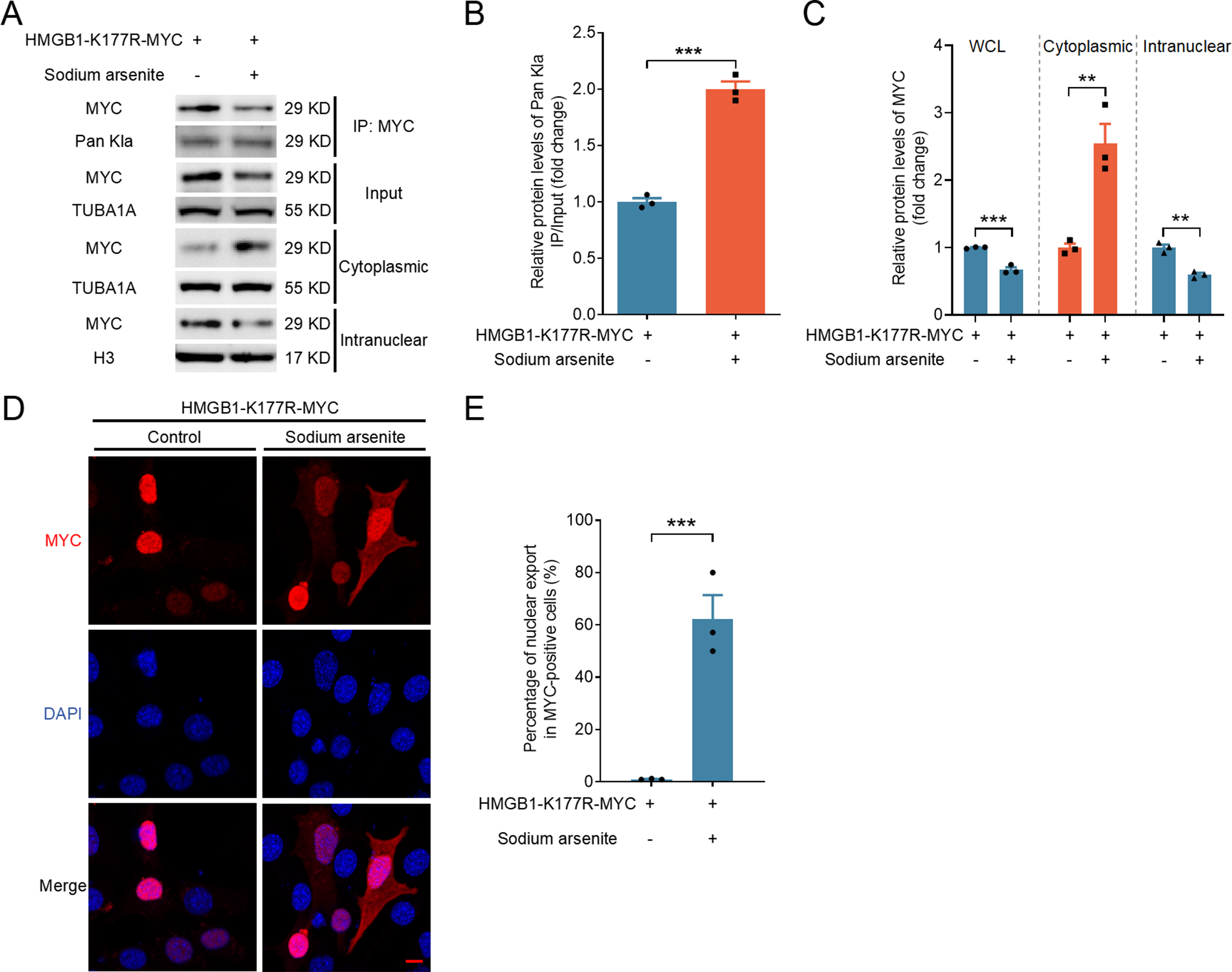


**Figure S16.** Sodium arsenite enhances HMGB1-K177R-MYC nuclear export and is not dependent on HMGB1 lactylation. A–E, NIH/3T3 cells transfected with HMGB1-K177R-MYC vectors for 24 h were treated with or without sodium arsenite (200 μM) for 2 h. Cells were harvested to separate nuclear and cytoplasmic fractions, the protein levels of MYC were analyzed using immunoblotting, and IP analysis of the lactylation and acetylation levels of MYC protein was performed (A). The protein bands were quantified (B and C), the subcellular localization of MYC was detected by immunofluorescence assay (D), and the proportion of cells with cytoplasmic localization of MYC was counted by laser confocal microscopy (E). Data are presented as mean ± s.e.m. (n = 3). ***P* < 0.01, ****P* < 0.001.


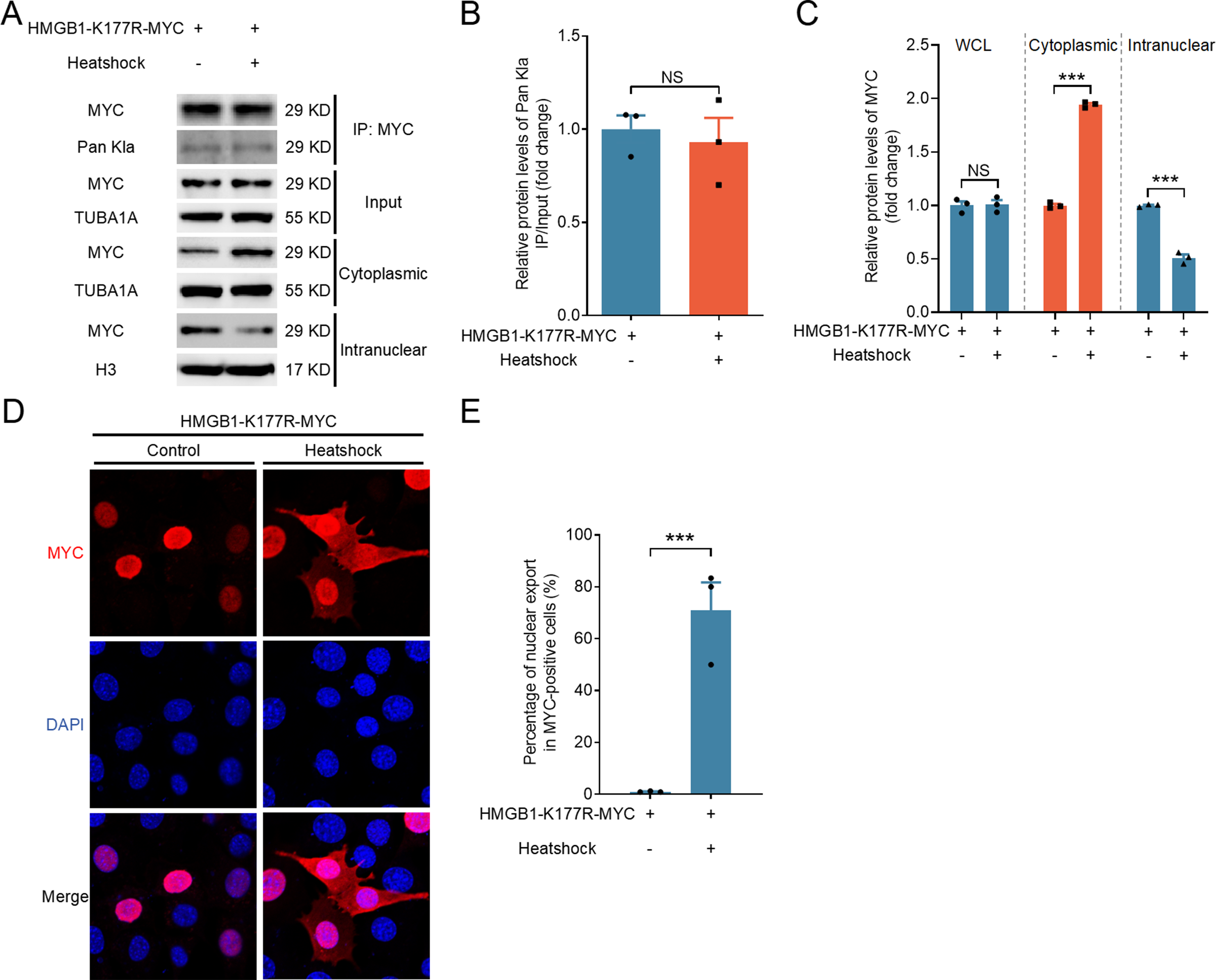


**Figure S17.** Heatshock promotes the nuclear export of HMGB1-K177R-MYC without affecting HMGB1 lactation. A–E, NIH/3T3 cells transfected for 24 h with HMGB1-K177R-MYC vectors were exposed or not exposed to a 43°C water bath for 0.5 h. Cells were harvested to separate nuclear and cytoplasmic fractions, the protein levels of MYC were analyzed using immunoblotting, and IP analysis of the lactylation and acetylation levels of MYC protein was performed (A). The protein bands were quantified (B and C), the subcellular localization of MYC was detected by immunofluorescence assay (D), and the proportion of cells with cytoplasmic localization of MYC was counted by laser confocal microscopy (E). Data are presented as mean ± s.e.m. (n = 3). ****P* < 0.001; NS, not significant (*P* > 0.05).


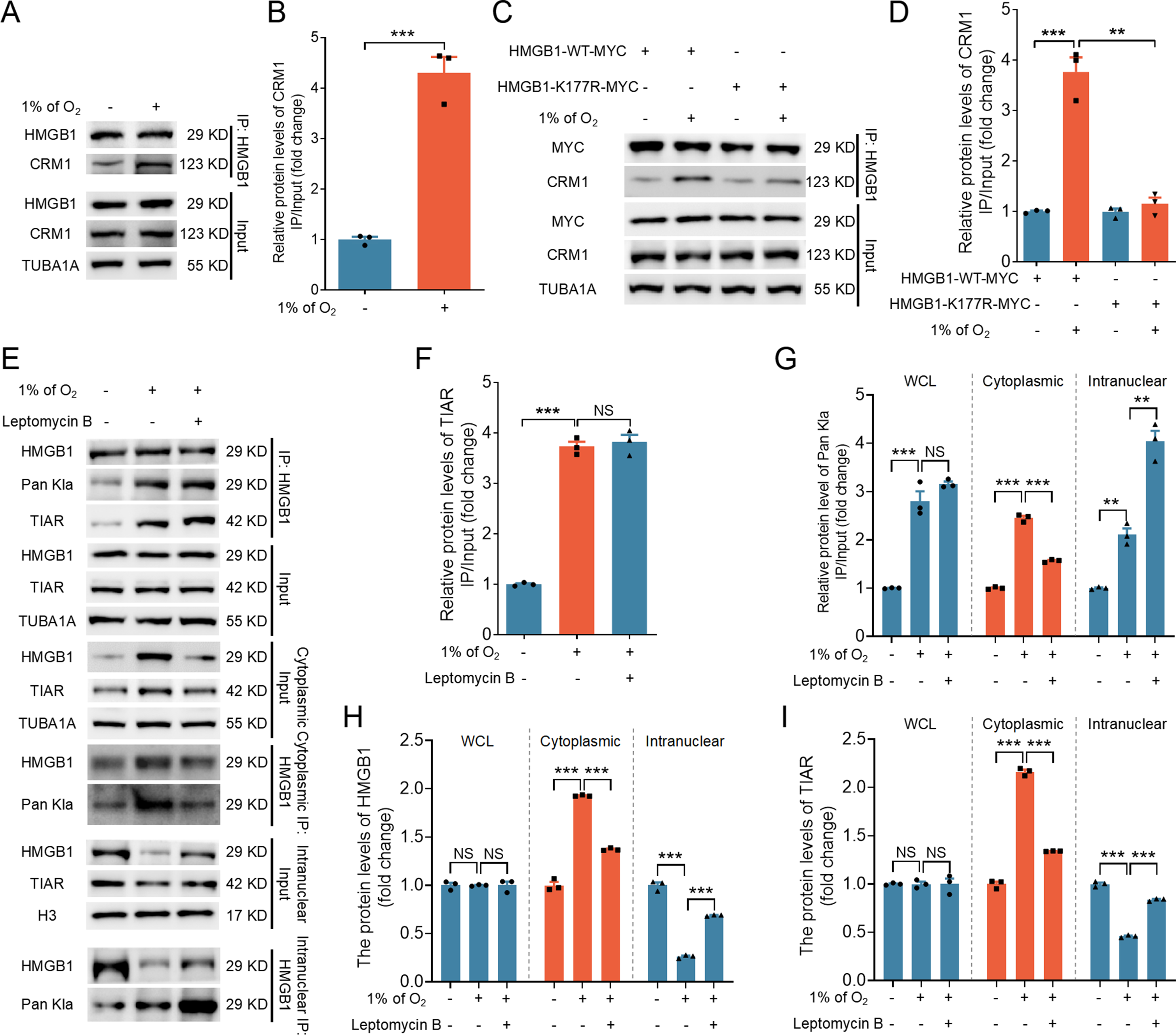


**Figure S18.** CRM1 facilitates the nuclear export of lactylated HMGB1 under hypoxia. A and B, NIH/3T3 cells were cultured under normoxic (21% of O_2_) or hypoxic (1% of O_2_) conditions for 2 h. IP was performed to assess the interaction between HMGB1 and CRM1 (A), and band intensities were quantified (B). C and D, Cells were transfected with vectors encoding HMGB1-WT-MYC or HMGB1-K177R-MYC for 24 h, followed by incubation under normoxia or hypoxia for an additional 2 h. IP was conducted to evaluate the interaction between MYC-tagged HMGB1 and CRM1 (C), and band intensities were quantified (D). E–I, Cells were pretreated with 10 nM Leptomycin B for 2 h, followed by normoxic or hypoxic culture for another 2 h. Whole-cell lysates or nuclear/cytoplasmic fractions were collected. IP was used to assess the interaction between HMGB1 and TIAR, as well as the lactylation levels of HMGB1 (E), and band intensities were quantified (F–I). Data are presented as mean ± s.e.m. (n = 3). ***P* < 0.01, ****P* < 0.001; NS, not significant (*P* > 0.05).

Supplementary Table S1 siRNA sequences.

| **siRNA Name** | **Sense（5'-3'）** | **Antisense（5'-3'）** |
| --- | --- | --- |
| *Scrambled siRNA*  *(SC)* | UUCUCCGAACGUGUCACGUTT | ACGUGACACGUUCGGAGAATT |
| *HMGB1-siRNA* | GGAAGAUGAUGAGGAGGAUTT | AUCCUCCUCAUCAUCUUCCTT |
| *TIAR-siRNA* | CCAUAUUGCUUUGUGGAAUTT | AUUCCACAAAGCAAUAUGGTT |
| *LDHA-siRNA* | CACGUACACGGAGACCUCGGUAUUA | UAAUACCGAGGUCUCCGUGUACGUG |
| *LDHB-siRNA* | CCGUGUCUACCAUGGUGAAGGGAAU | AUUCCCUUCACCAUGGUAGACACGG |

**Supplementary Table S2 The reciprocal amino acid sequences were visualised using Pymol software.**

| **Pose** | **Protein** | **Interface residue** |
| --- | --- | --- |
| 1 | TIAR | 12Y, 14G, 15N, 41I, 43E, 45T, 46S, 47N, 48D, 50Y, 52F, 79K, 81N, 84T, 86P, 90K, 127R, 129V, 132 M, 133A, 138K, 140Y, 142F, 155I, 156V, 157H, 159G, 160G, 167Q, 174T, 176K, 177P, 179A, 180P, 181K, 189K, 192R, 195D, 367Y, 368G, 369M, 370A, 371S, 372F, 373P, 374T |
|  | HMGB1 | 1M, 24R, 27H, 28K, 31H, 32P, 34A, 35S, 36V, 37N, 38F, 41F, 50 K, 53S, 54A, 55K, 197E, 201E, 203E, 204E, 206E, 207D, 208E, 209E, 210E, 211D, 212D, 213D, 214D, 215E, 216C |
| 2 | TIAR | 10T, 12Y, 39K, 41I, 43E, 45T, 46S, 47N, 50Y, 52F, 81N, 82W, 83A, 84 T, 85T, 86P, 90K, 127R, 129V, 130 K, 131D, 132M, 133A, 138K, 140Y, 155 I, 156V, 157H, 159G, 160G, 161Q, 162W, 167Q, 172W, 176K, 177P, 179A, 180P, 181K, 189K, 364Q, 365A, 366G, 367Y, 368G, 369M, 370A, 371S, 372F, 373P, 374T, 375Q |
|  | HMGB1 | 1M, 2G, 3K, 4G, 10R, 24R, 28 K, 32P, 33D, 34A, 35S, 36V, 37N, 47E, 50K, 51T, 52M, 53S, 54A, 57K, 203E, 204E, 205D, 206E, 207D, 208E, 209E, 210E, 211D, 212D, 213D, 214D, 215E, 216C |
| 3 | TIAR | 12Y, 47N, 52F, 81N, 82W, 84T, 86P, 88 S, 90K, 100F, 120F, 127R, 129V, 130K, 132M, 138K, 140Y, 142F, 157H, 169R, 171N, 172W, 173A, 174T, 175R, 176K, 177P, 179A, 181K, 367Y, 368G, 369M, 370A, 371S, 372F, 373P, 374T, 375Q, 376C |
|  | HMGB1 | 1M, 2G, 3K, 4G, 5D, 6P, 8K, 9P, 10R, 11G, 24R, 36V, 38F, 54A, 57K, 71Y, 78Y, 80P, 81P, 83G, 84E, 85T, 196D, 199E, 200E, 201E, 203E, 204E, 205D, 206E, 207D, 208E, 209E, 210E, 211D, 212D, 213D, 214D, 215E, 216C |
| 4 | TIAR | 4D, 5D, 6G, 7Q, 8P, 12Y, 45T, 46S, 47N, 52F, 81N, 82W, 83A, 84T, 85T, 86P, 87S, 88S, 89Q, 90K, 91K, 93T, 110T, 125D, 126A, 127R, 129V, 130K, 131D, 132M, 138K, 140Y, 142F, 155I, 156V, 159G, 160G, 162W, 167Q, 171N, 176K, 177P, 178P, 179A, 180P, 181K, 189K, 194E, 195D, 198N, 344G, 345F, 346G, 347A, 348Q, 365A, 366G, 367Y, 368G, 369M, 370A, 371S, 372F, 373P |
|  | HMGB1 | 1M, 24R, 27H, 28K, 30K, 31H, 32P, 33D, 34A, 35S, 36V, 50K, 51T, 52M, 53S, 54A, 57K, 61E, 111P, 112K, 114K, 115G, 116E, 118P, 182K, 186E, 193D, 194E, 196D, 197E, 198E, 199E, 200E, 201E, 202E, 203E, 204E, 205D, 206E, 207D, 208E, 209E, 210E, 211D, 212D, 214D, 215E, 216C |
| 5 | TIAR | 10T, 12Y, 37S, 39K, 41I, 45T, 47N, 50Y, 52F, 83A, 84T, 85T, 86P, 90K, 100F, 127R, 129V, 130K, 131D, 132M, 138K, 140Y, 142F, 148K, 149L, 152E, 153N, 156V, 157H, 169R, 174T, 176K, 179A, 180P, 181K, 182S, 361P, 362P, 363N, 364Q, 365A, 366G, 367Y, 368G, 369M, 370A, 371S, 372F |
|  | HMGB1 | 1M, 2G, 3K, 4G, 5D, 6P, 8K, 47E, 50K, 51T, 52M, 57K, 80P, 81P, 82K, 83G, 84E, 85T, 86K, 87K, 88K, 90K, 110R, 122I, 199E, 202E, 203E, 205D, 206E, 207D, 208E, 209E, 210E, 211D, 212D, 213D, 214D, 215E, 216C |

**Supplementary material S3 Identification of the lactylation sites of the HMGB1 protein by means of mass spectrometry (See next page).**


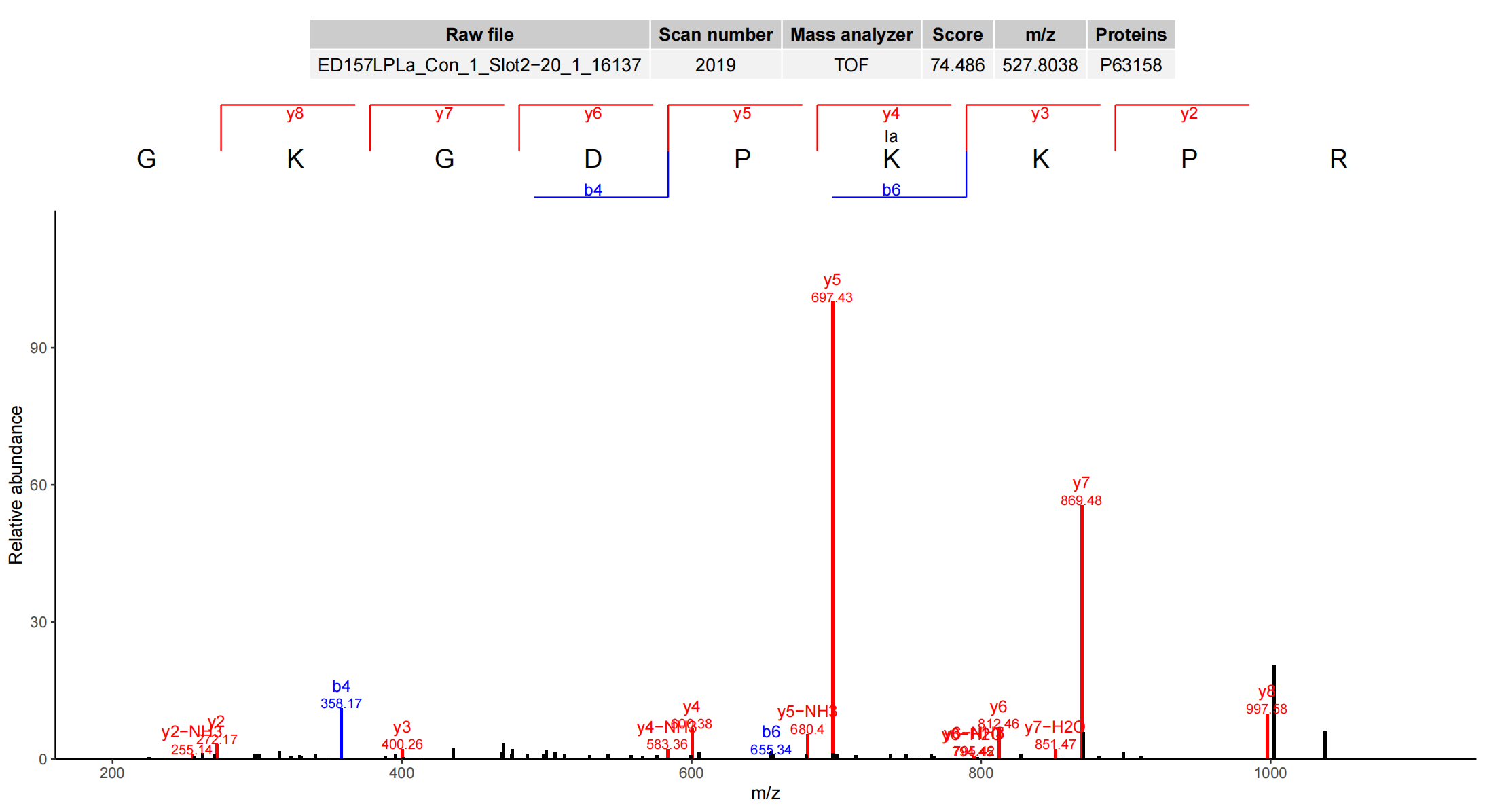


K7


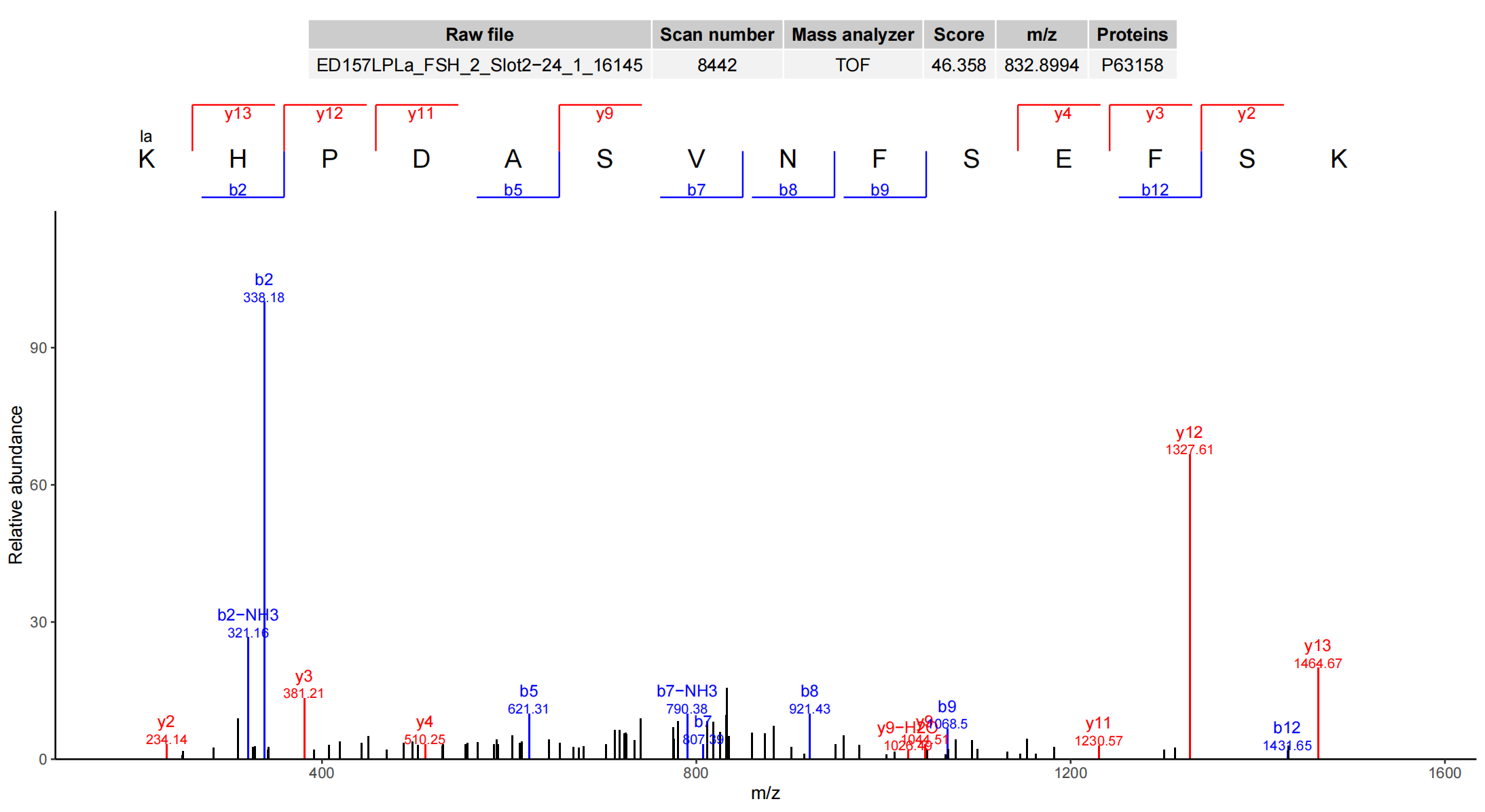


K30


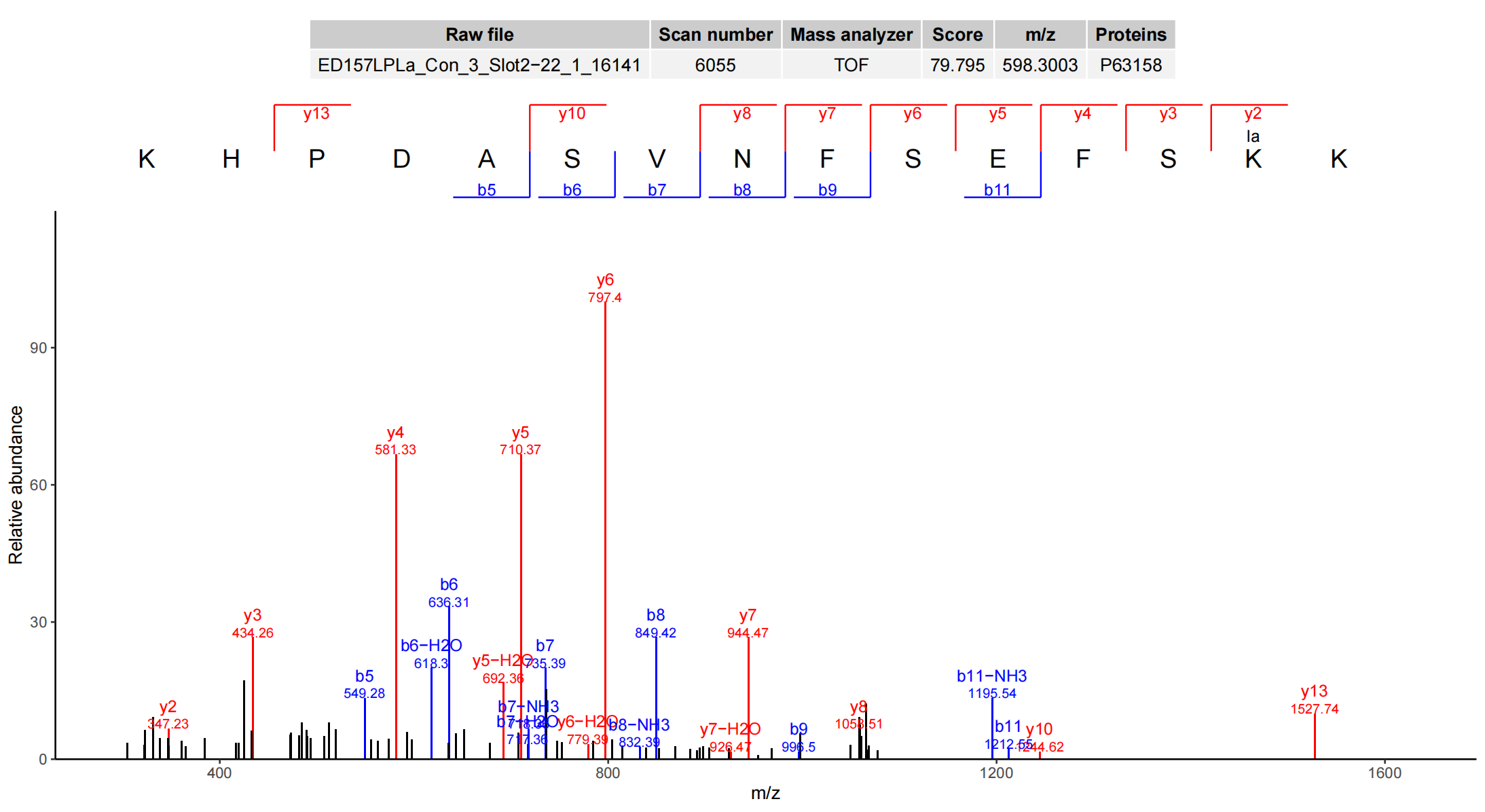


K43


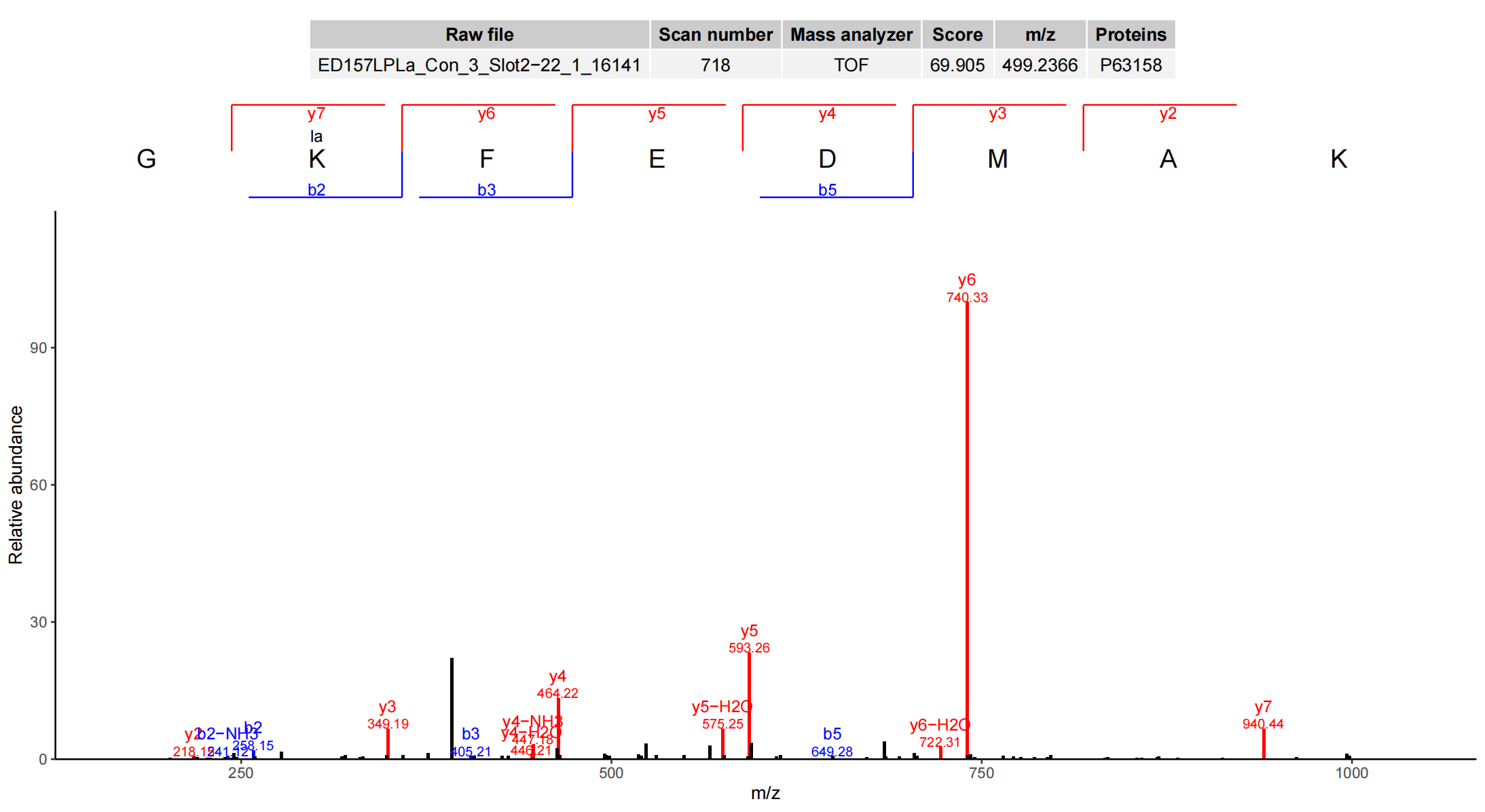


K59


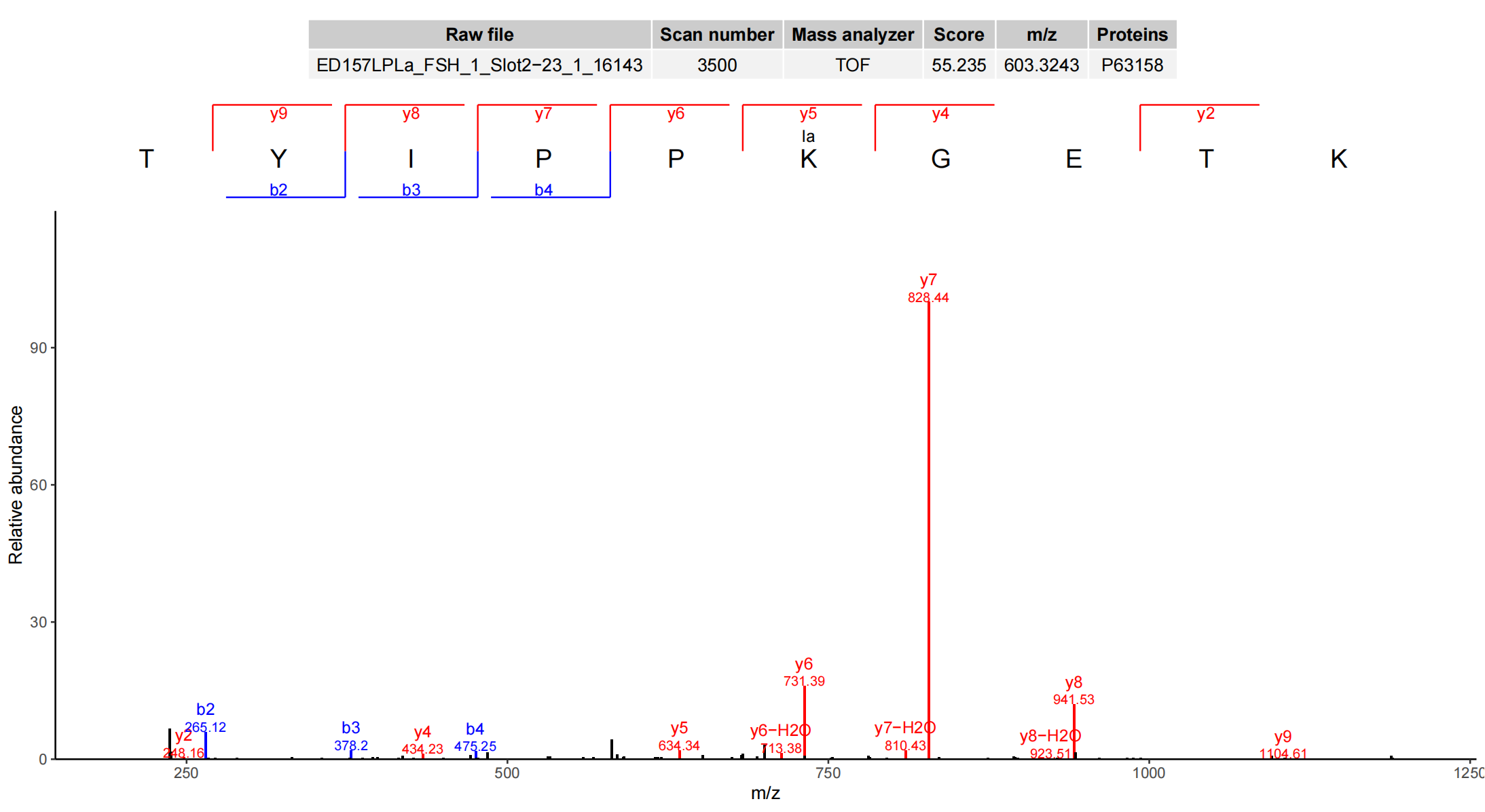


K82


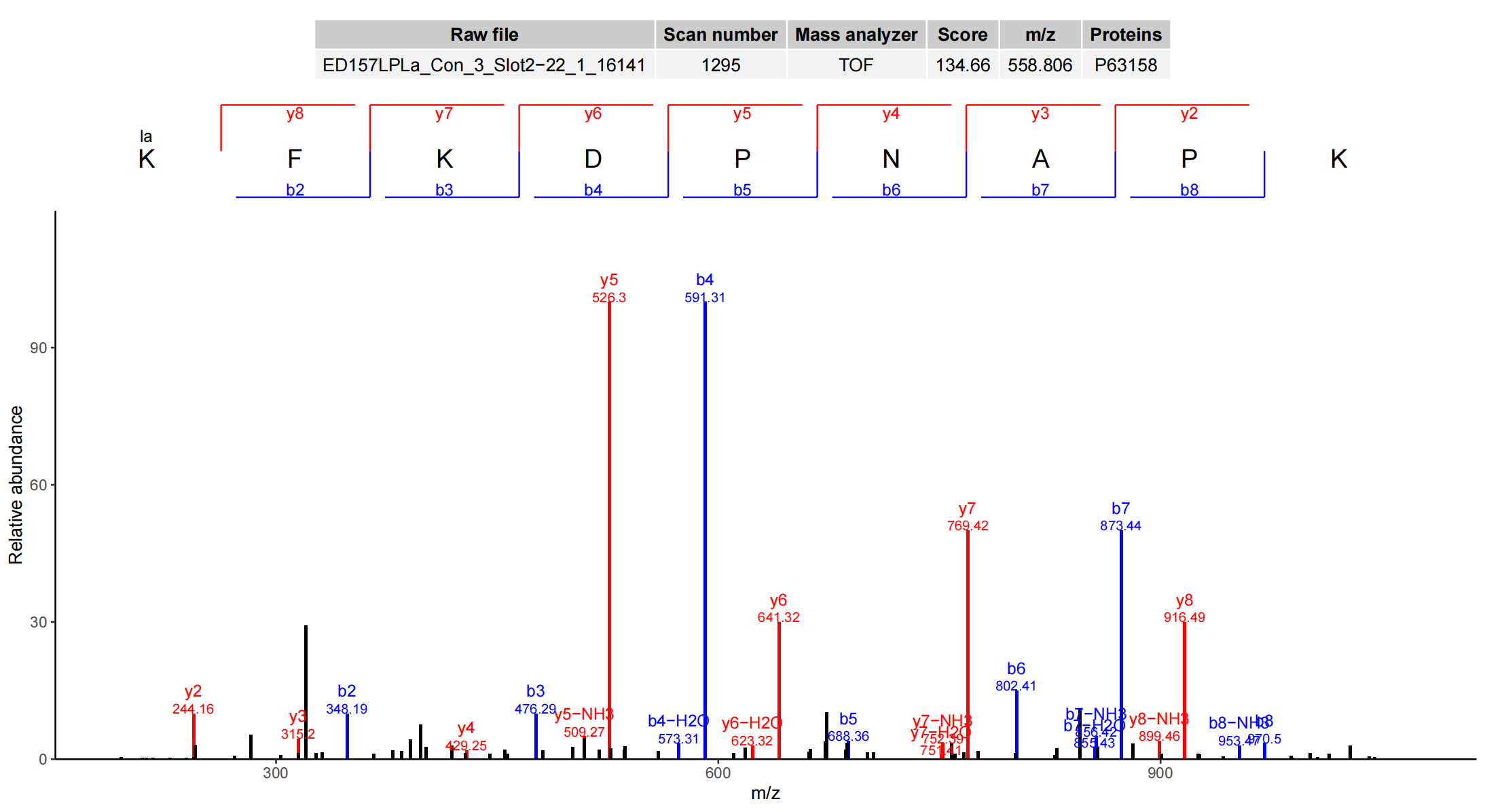


K88


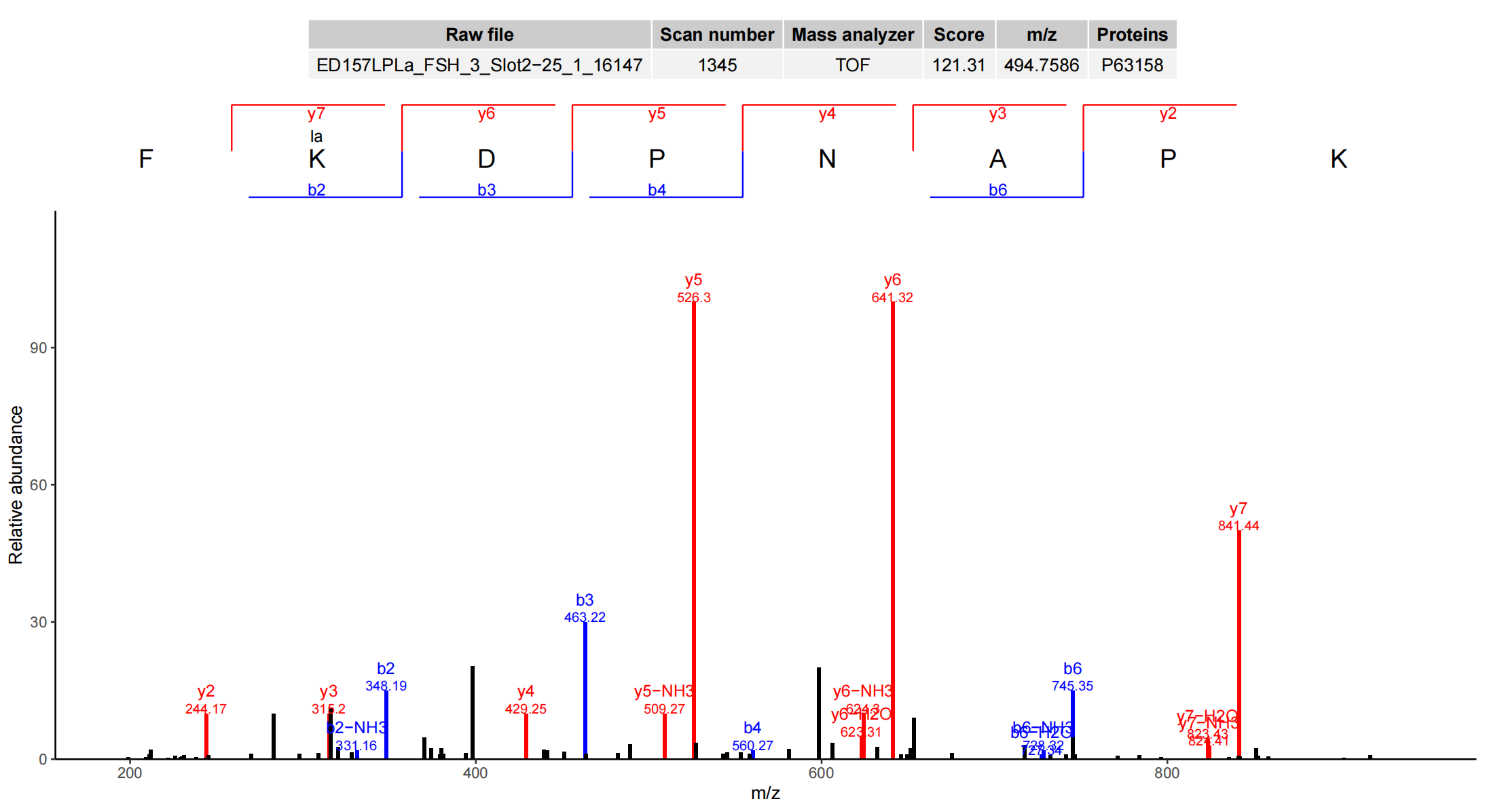


K90


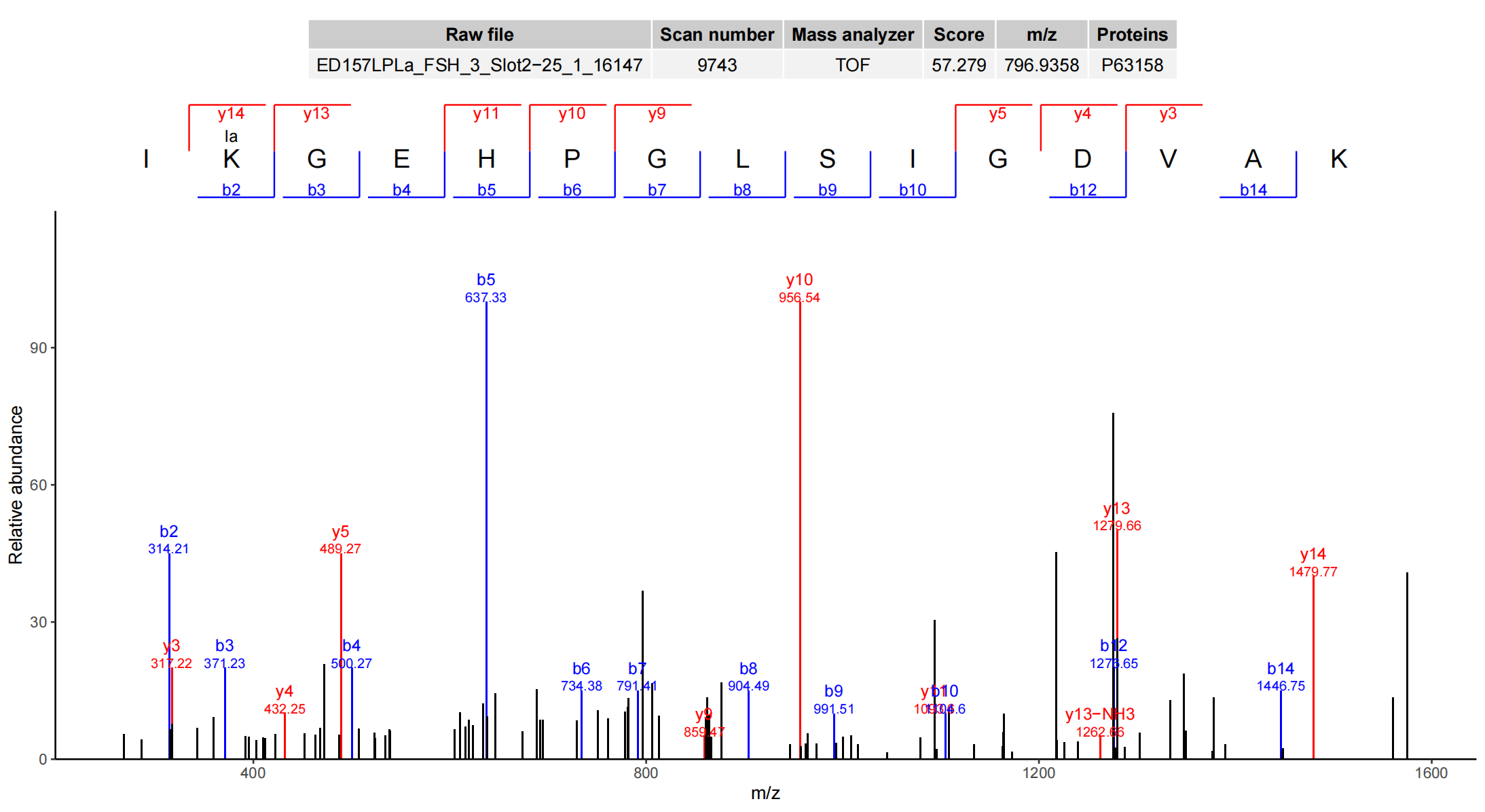


K114


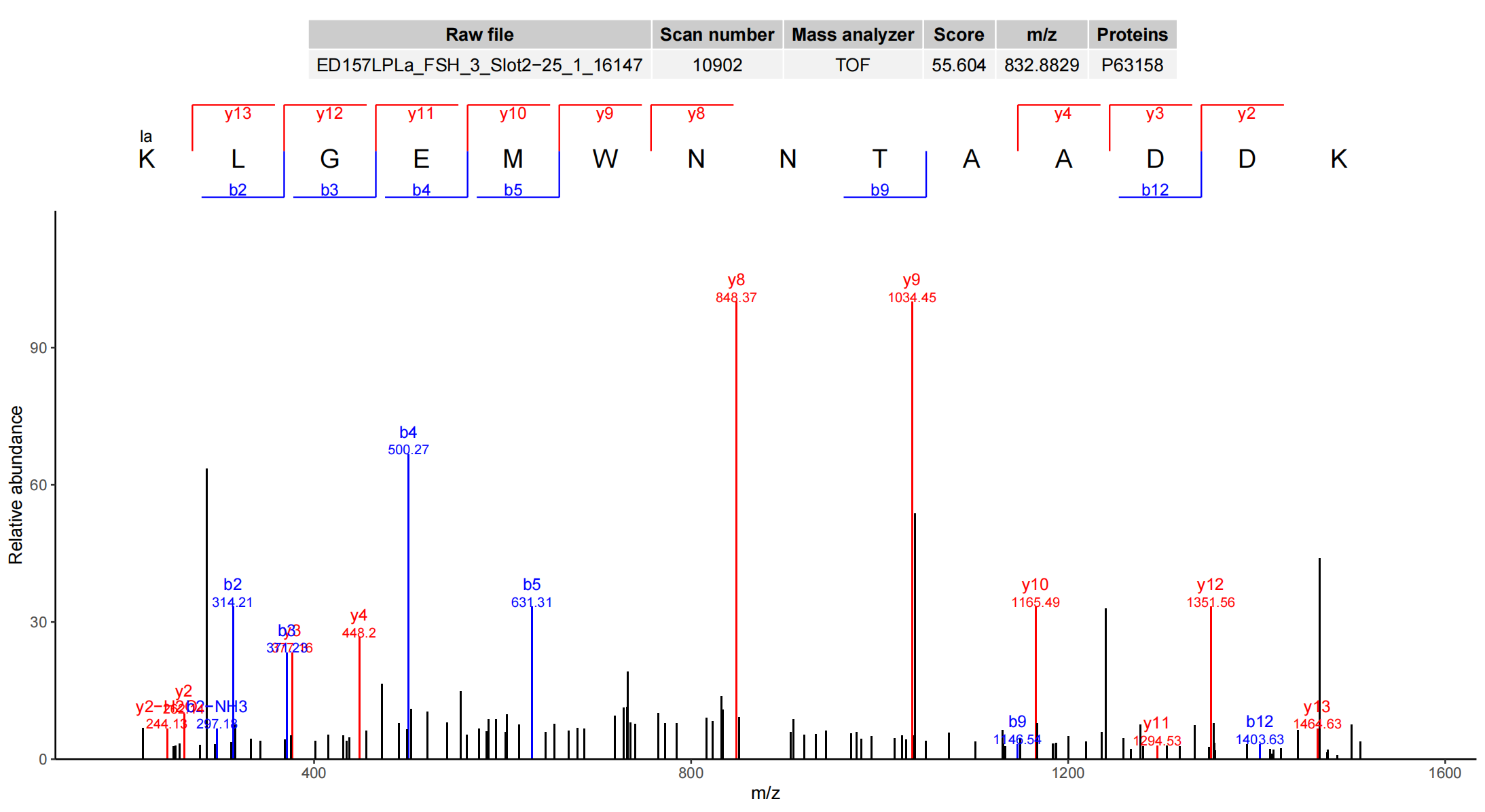


K128


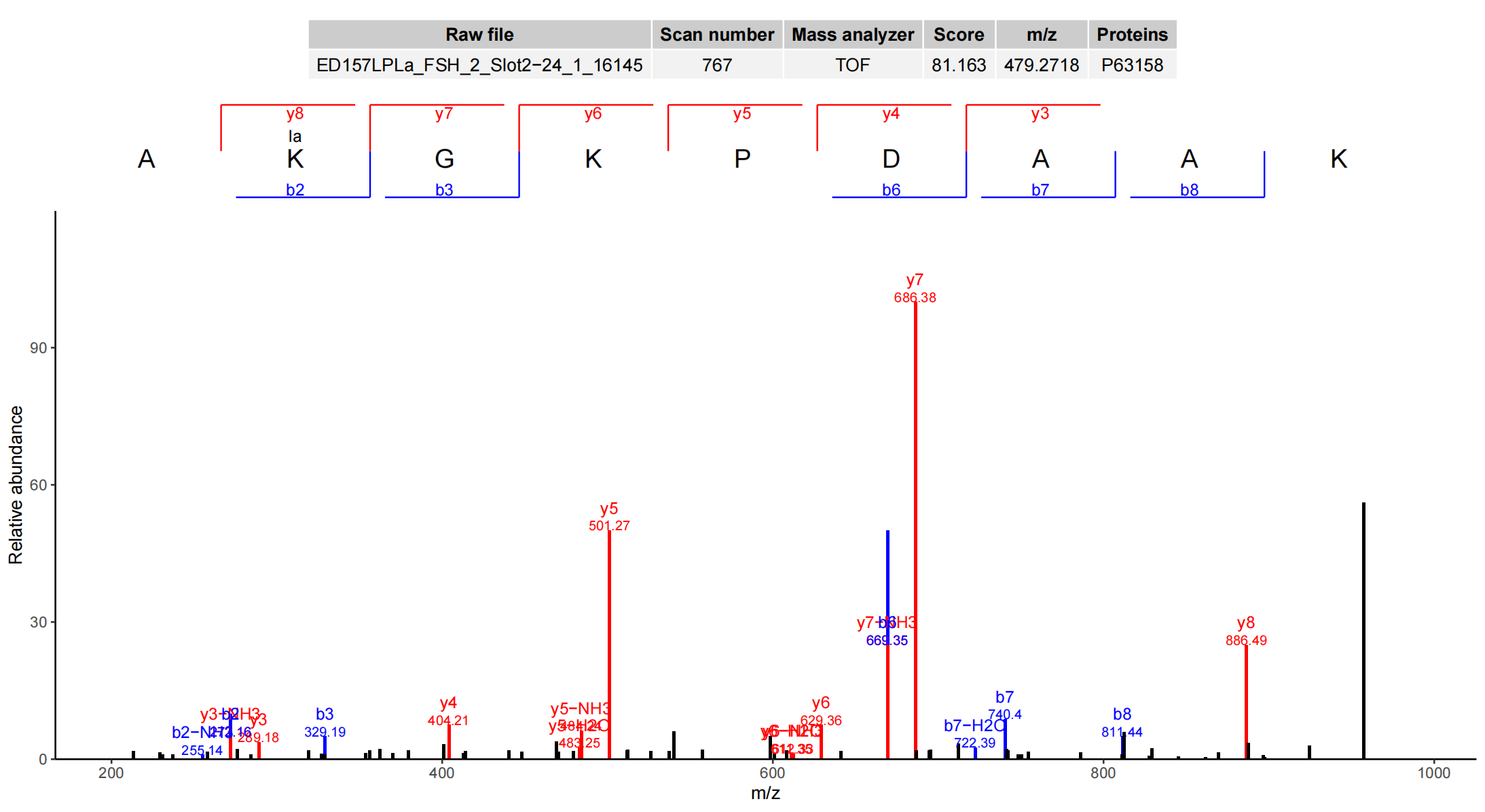


K165


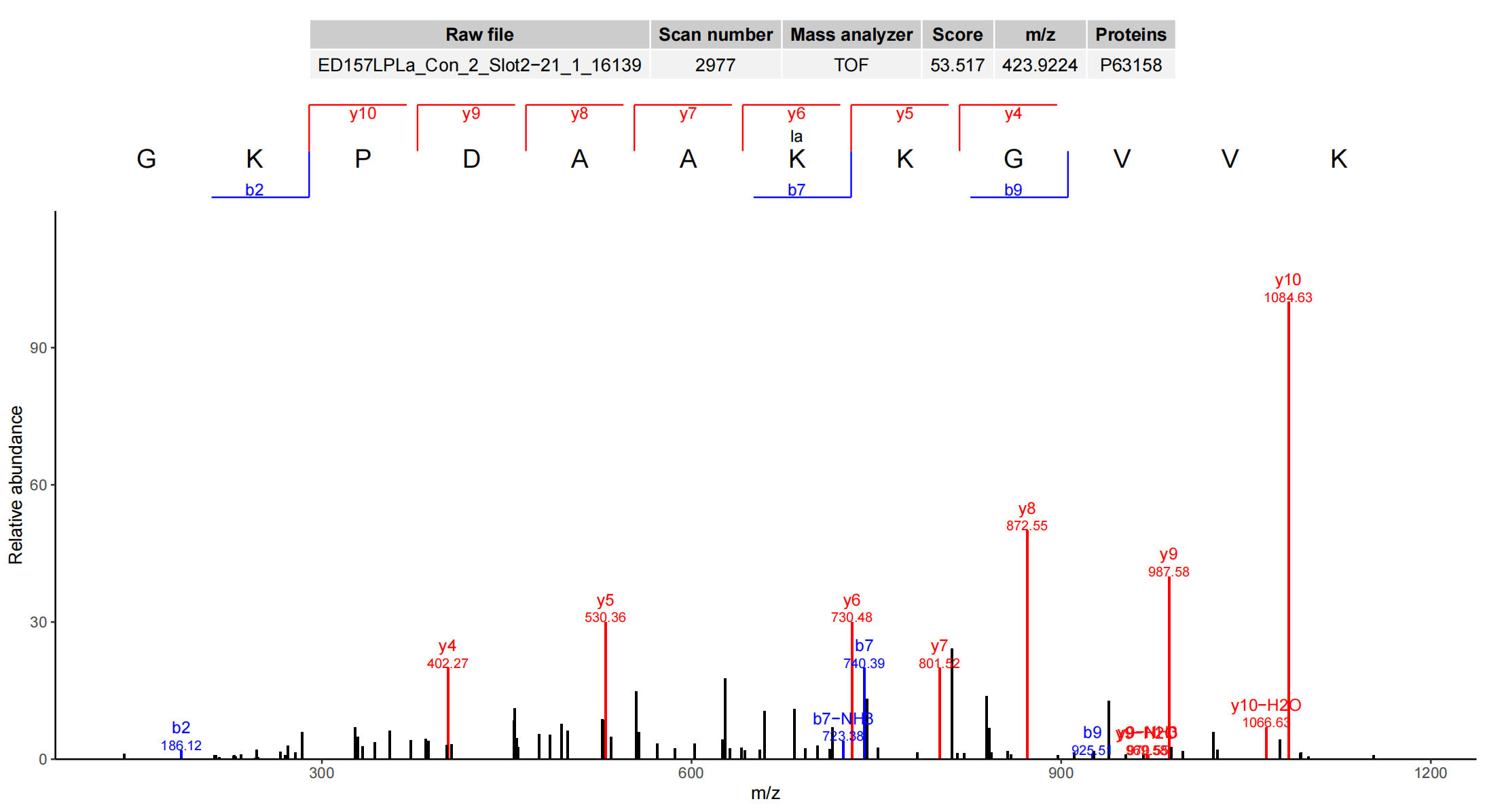


K172


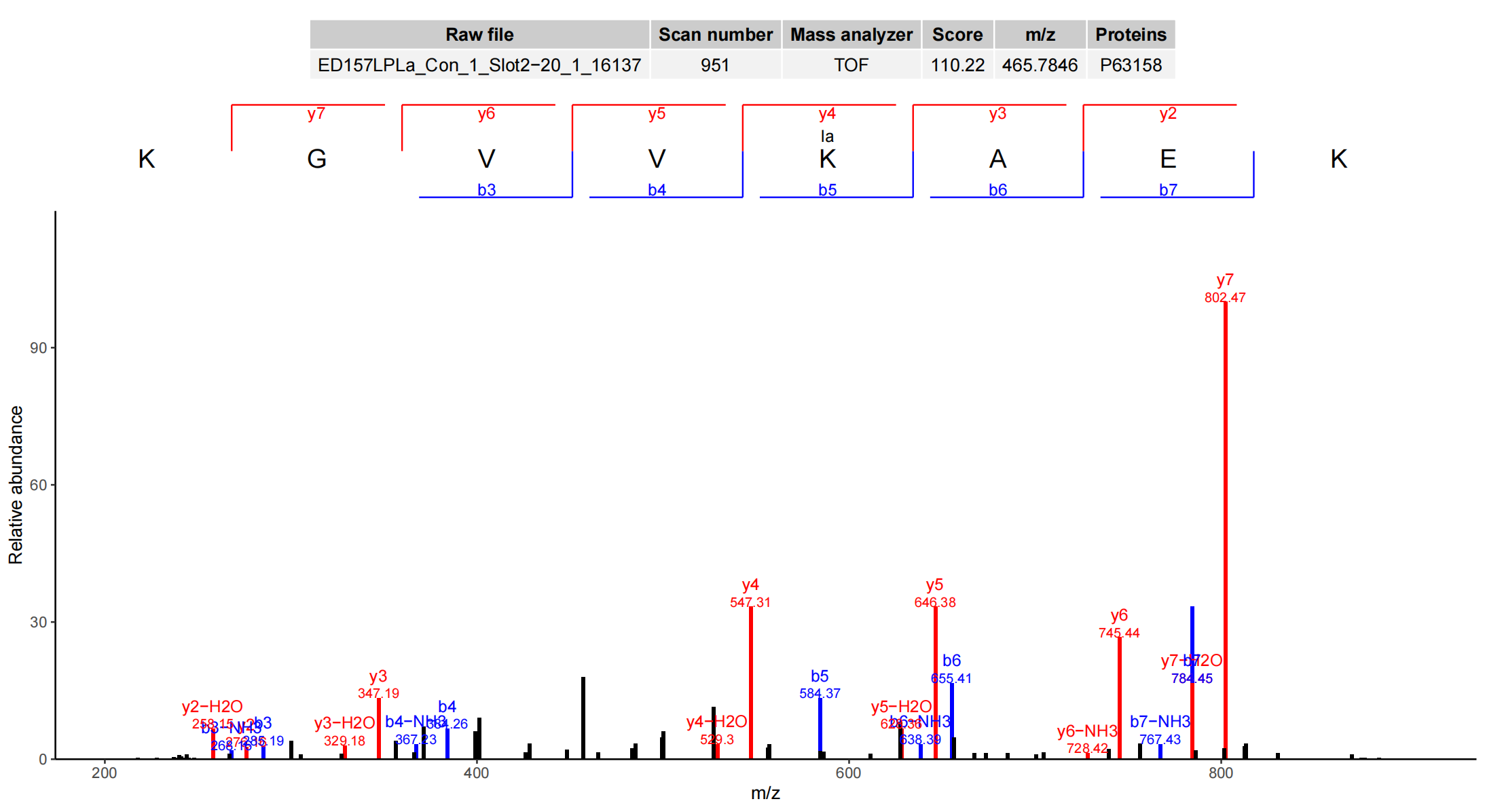


K177
