# Supplementary material for: Lactylation of HMGB1 at K177 Drives Nuclear Export of TIAR to Promote Hypoxia‐Induced Stress Granule Formation
Source: Adv Sci (Weinh). 2025 Aug 11;12(41):e04896. doi: 10.1002/advs.202504896 (PMC12591107; doi:10.1002/advs.202504896)
Supplement: Supplementary file 2 — Supporting Information [file ADVS-12-e04896-s002.pdf]

Fig.1G

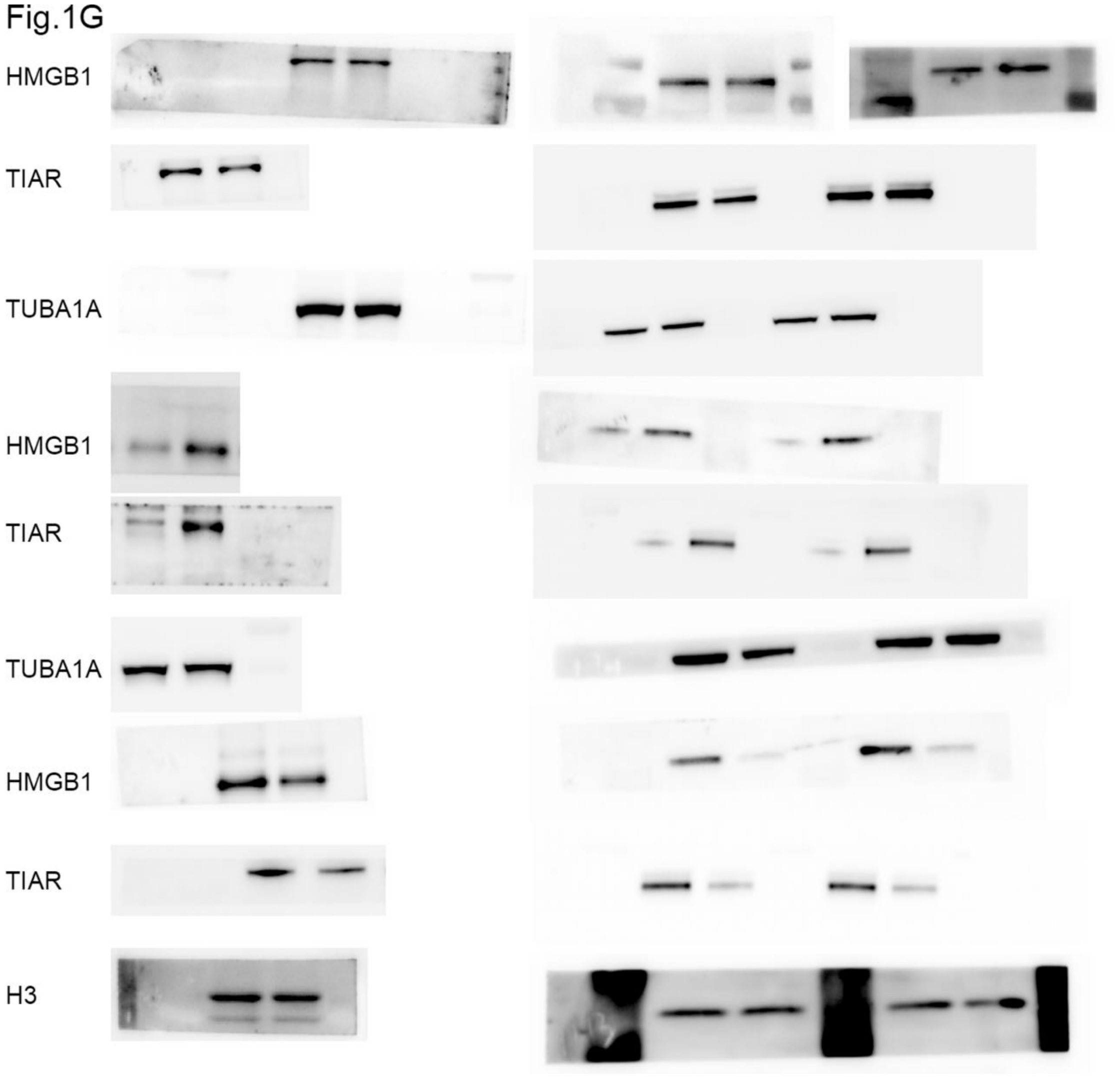

Fig. 2D

HMGB1

TIAR

TUBA1A

TIAR

TUBA1A

TIAR

H3

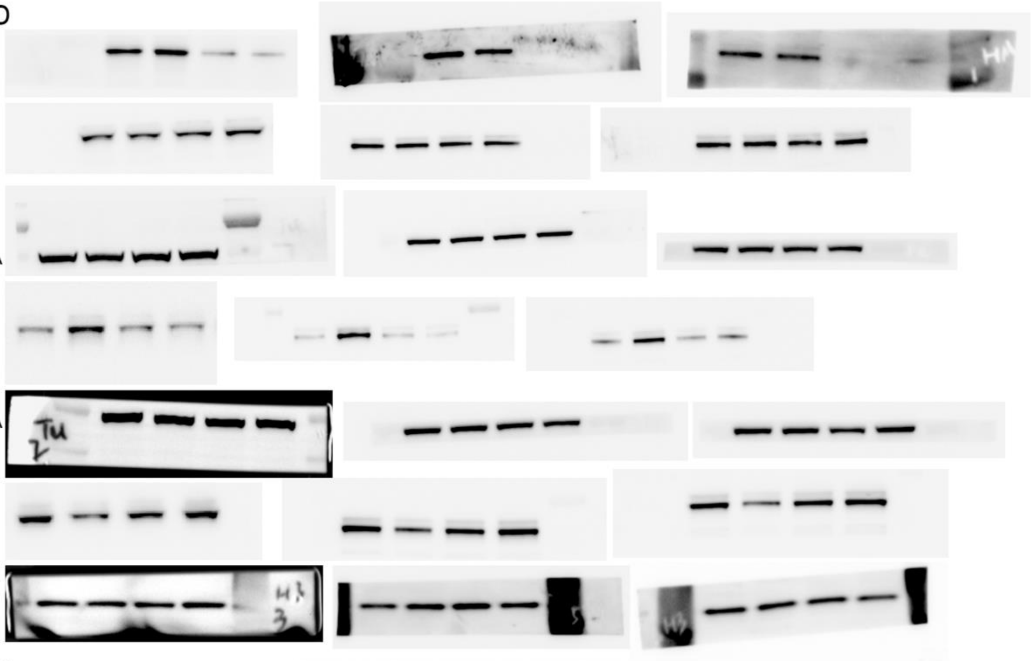

Fig. 2H

TIAR

HMGB1

TUBA1A

HMGB1

TUBA1A

HMGB1

H3

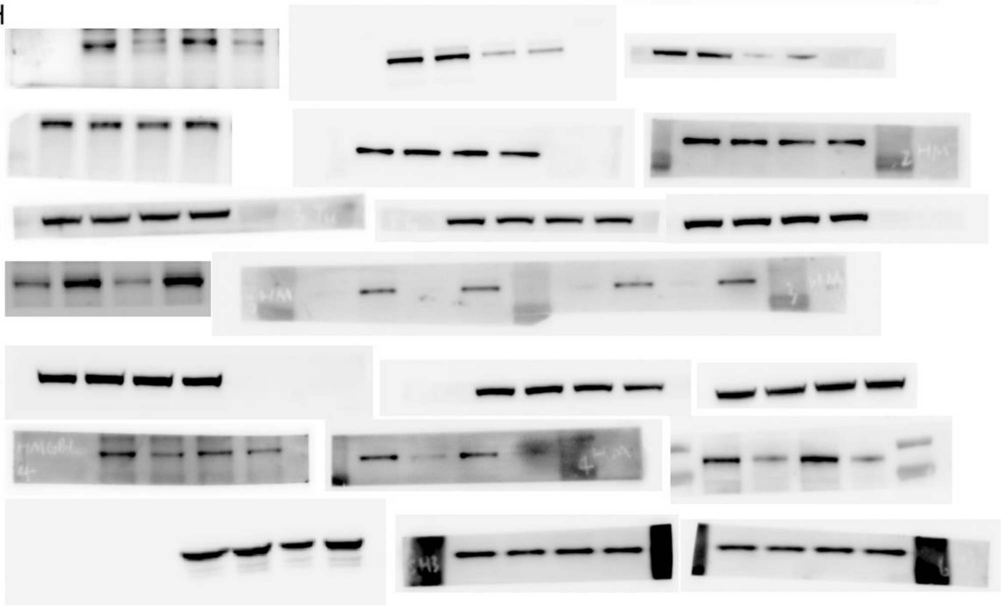

Fig. 3A

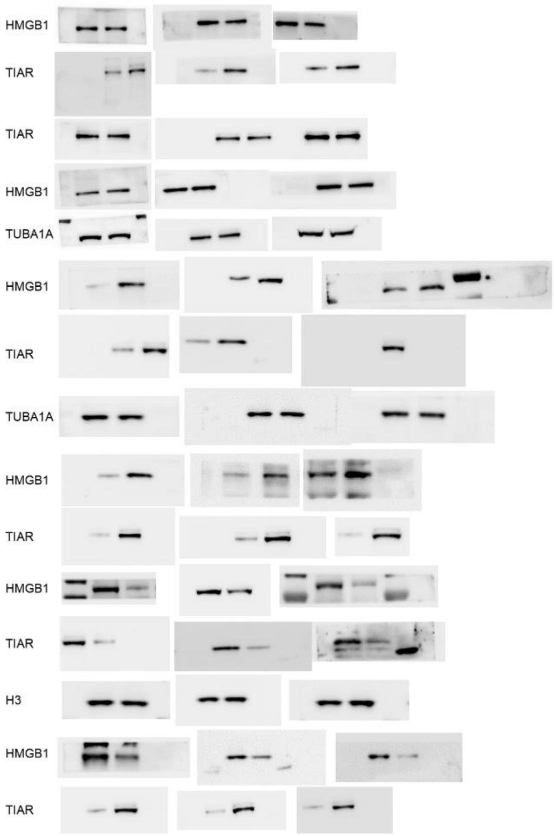

HMGB1
TIAR
TIAR
HMGB1
TUBA1A
HMGB1
TIAR
TUBA1A
HMGB1
TIAR
HMGB1
TIAR
H3
HMGB1
TIAR

Fig. 3G

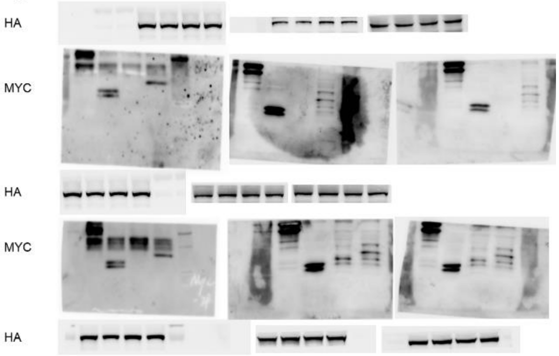

HA
MYC
HA
MYC
HA

Fig. 3I

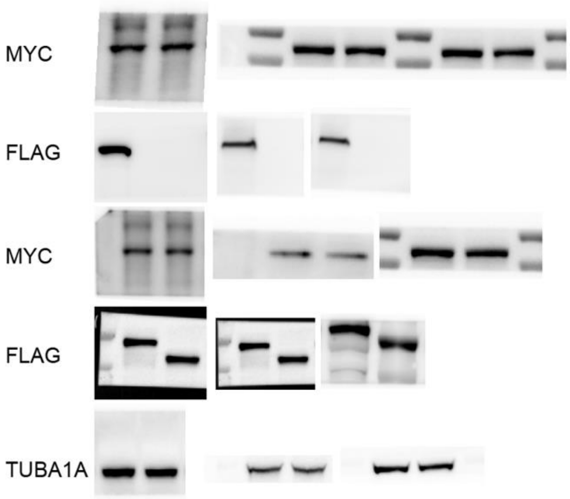

Fig. 3K

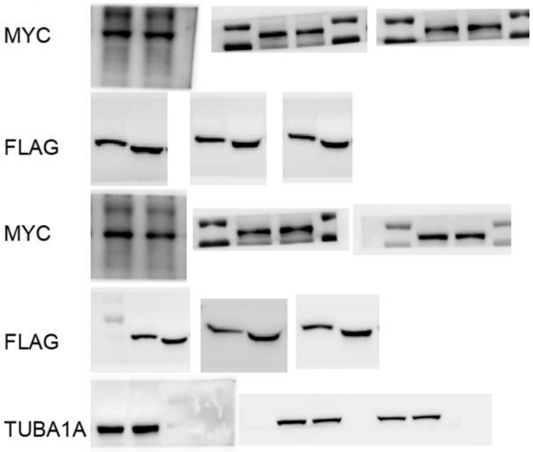

Fig. 3M

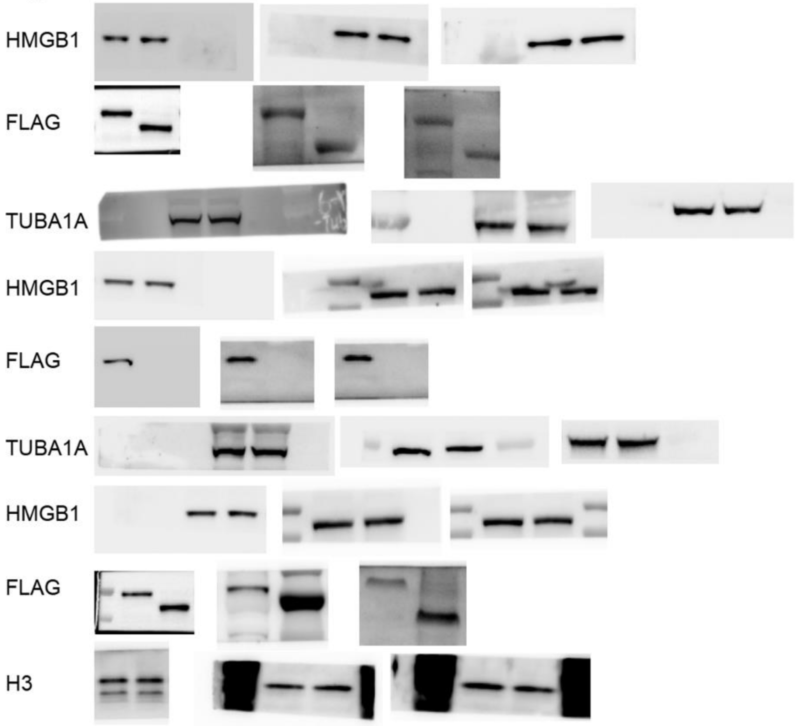

Fig. 4L

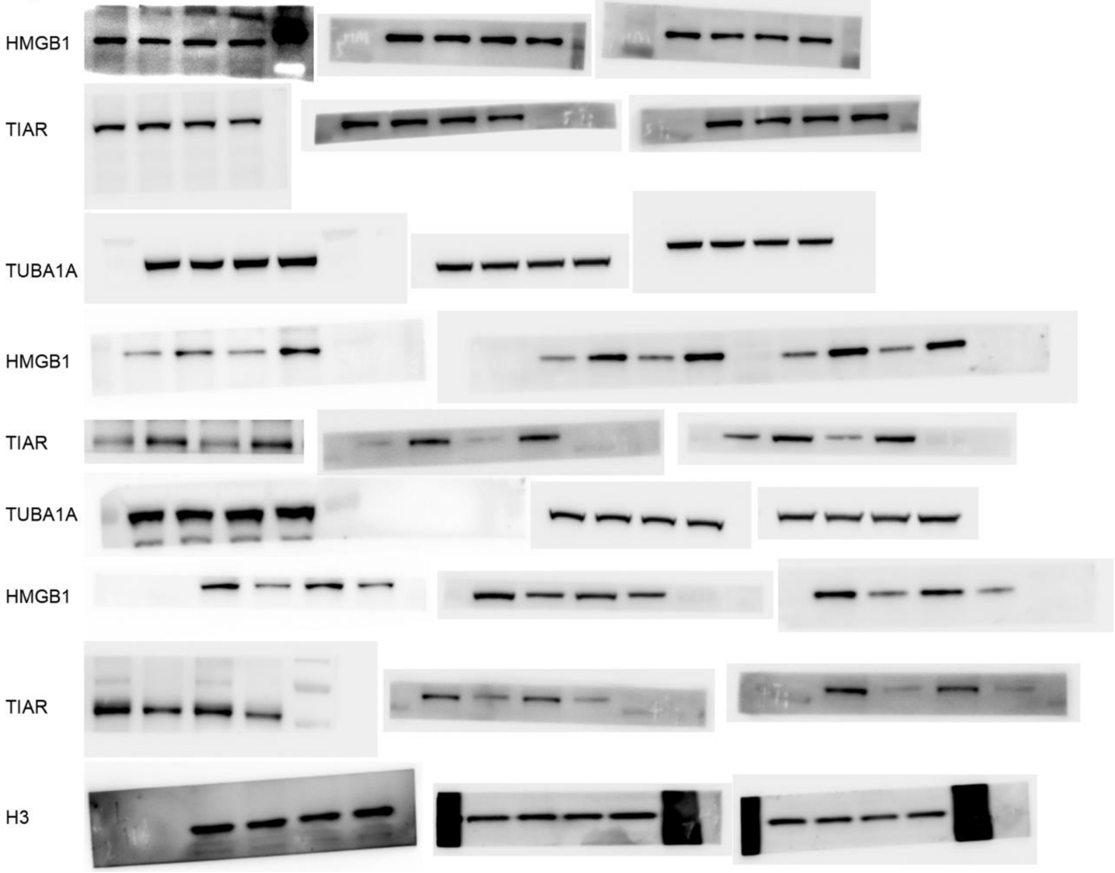

Fig. 5A

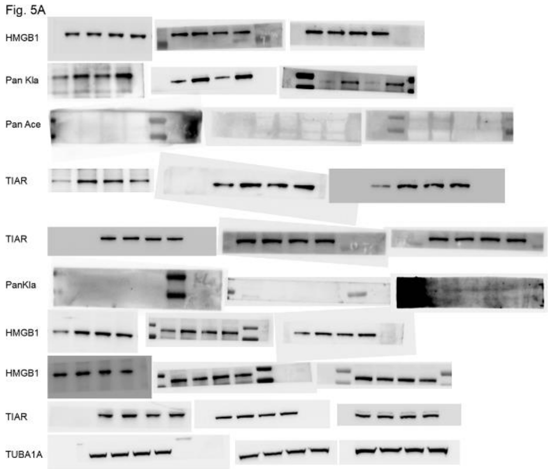

Fig. 5D

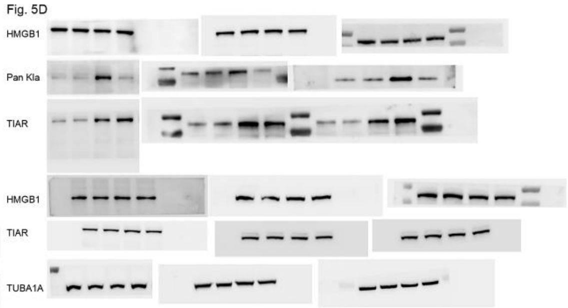

Fig. 5F

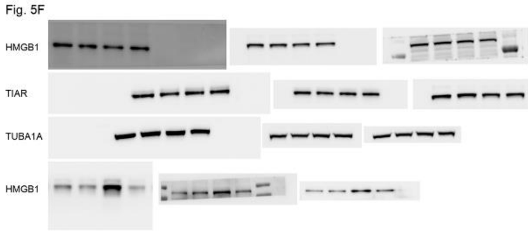

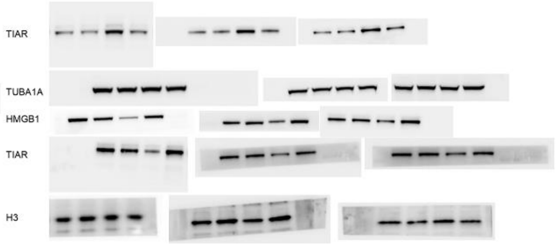

Fig. 6A

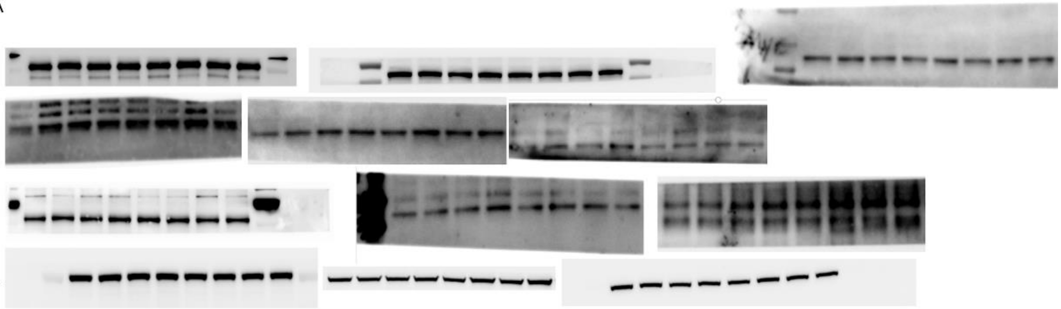

MYC
Pan Kla
MYC
TUBA1A

Fig. 6B

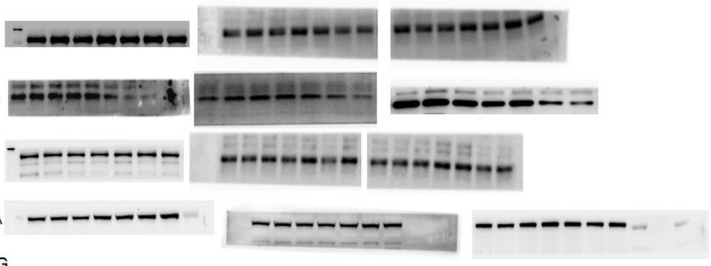

MYC
Pan Kla
MYC
TUBA1A

Fig. 6G

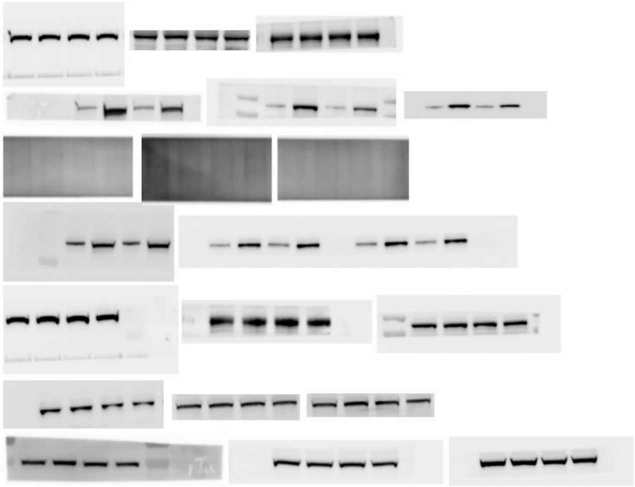

MYC
Pan Kla
Pan Ace
TIAR
MYC
TIAR
TUBA1A

Fig. 6l

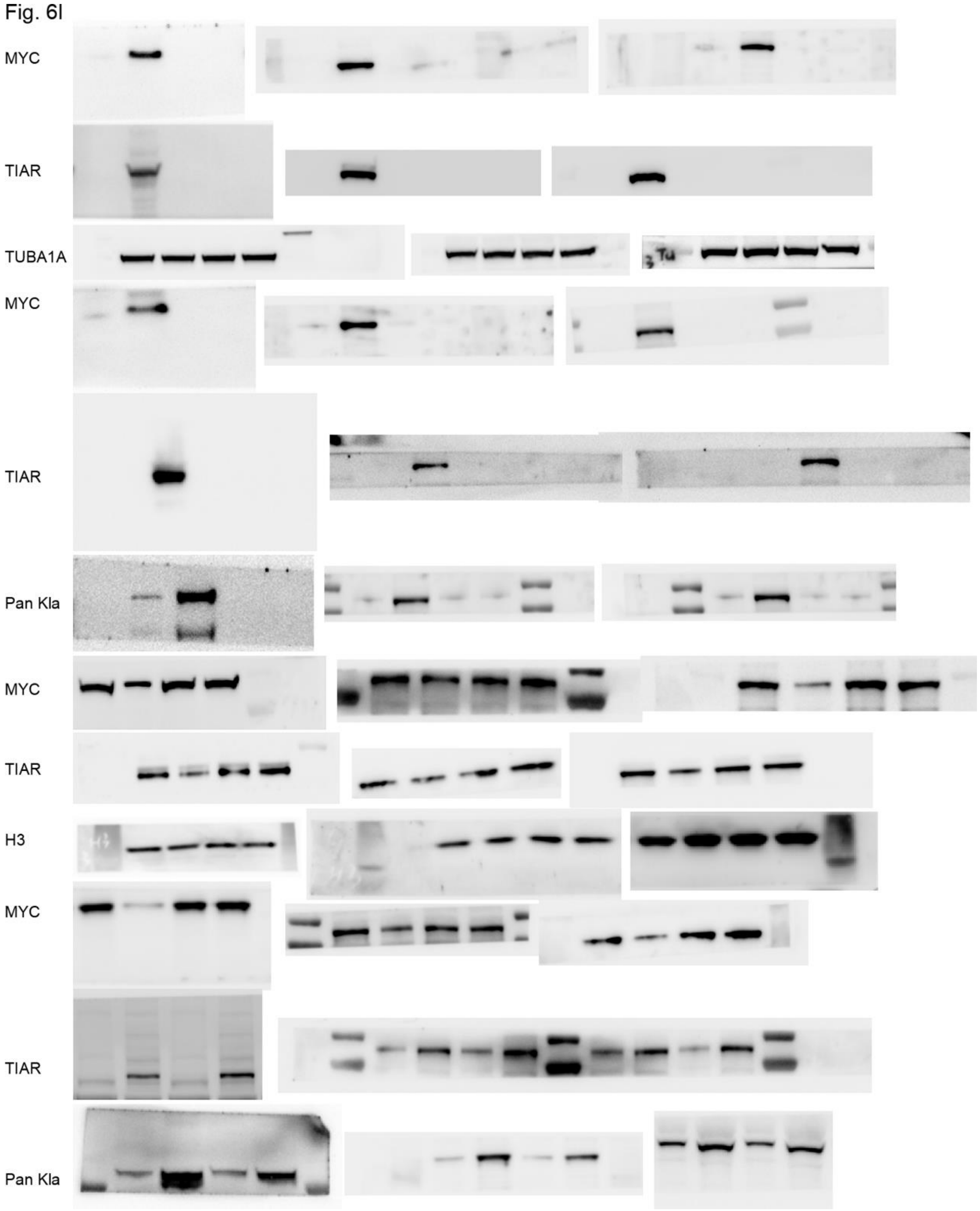

Fig. 7B

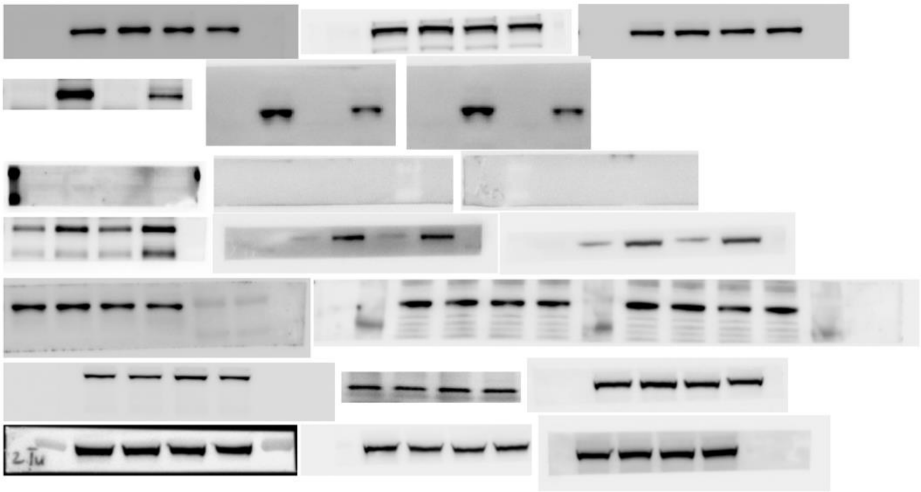

MYC
Pan Kla
Pan Ace
TIAR
MYC
TIAR
TUBA1A

Fig. 7D

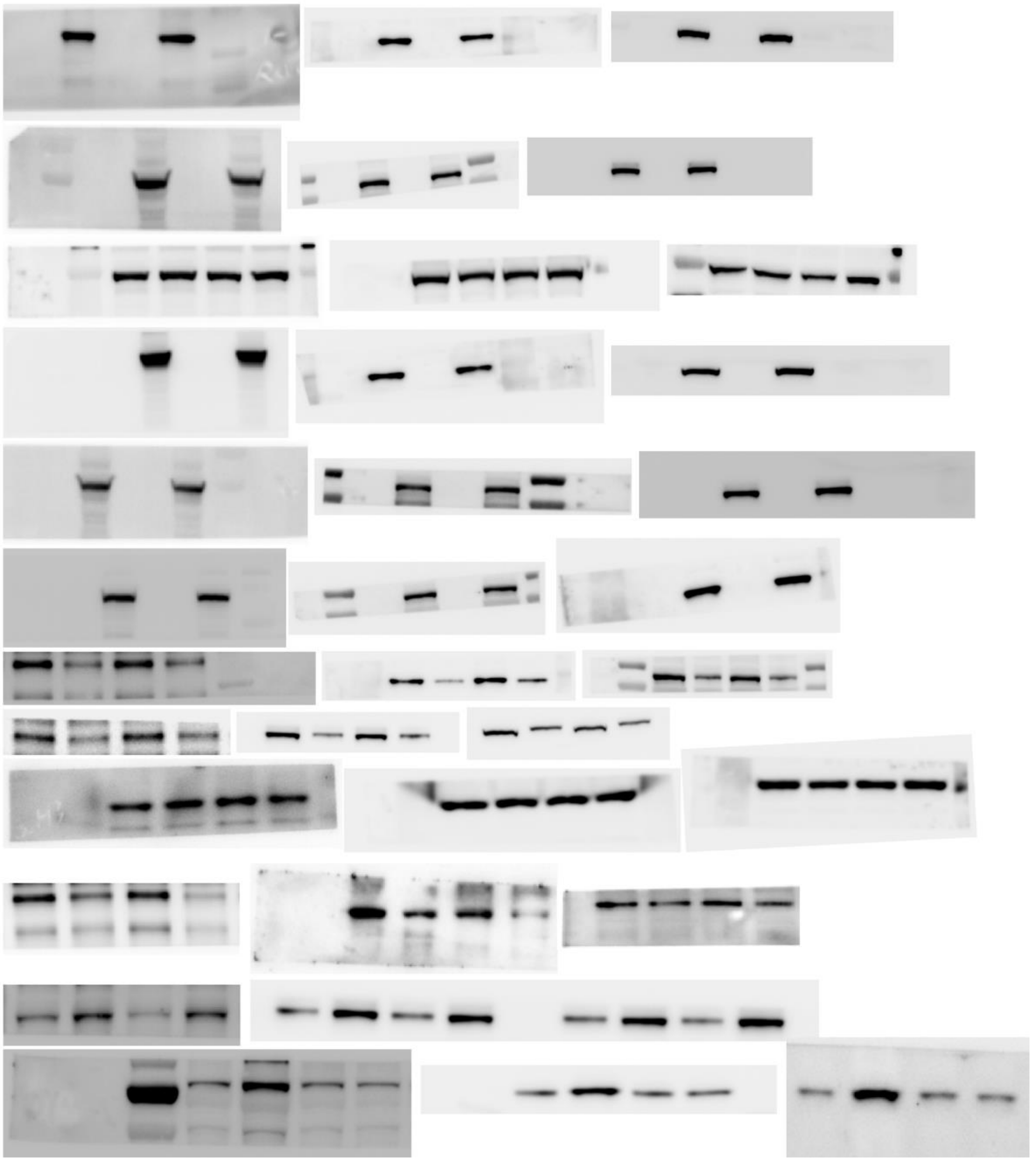

MYC
TIAR
TUBA1A
MYC
TIAR
Pan Kla
MYC
TIAR
H3
MYC
TIAR
Pan Kla

Fig. 8A

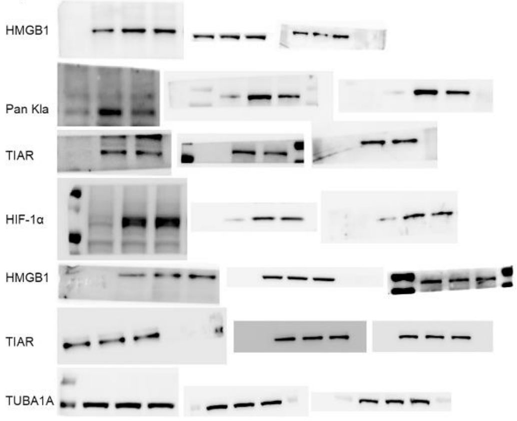

Fig. 8E

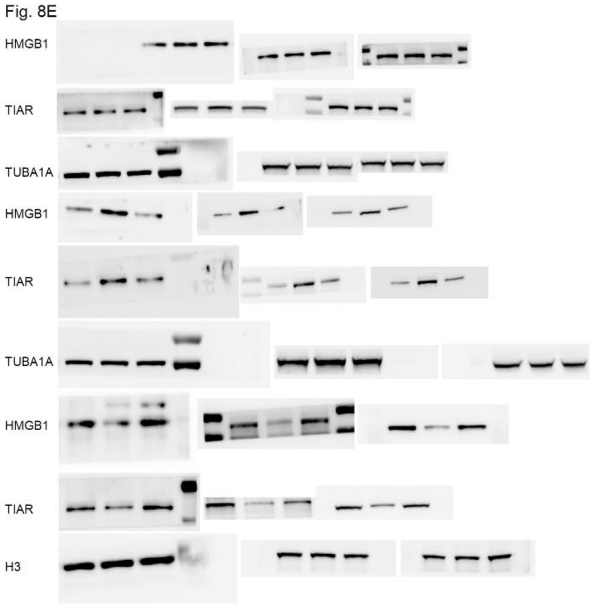

Fig. S1A

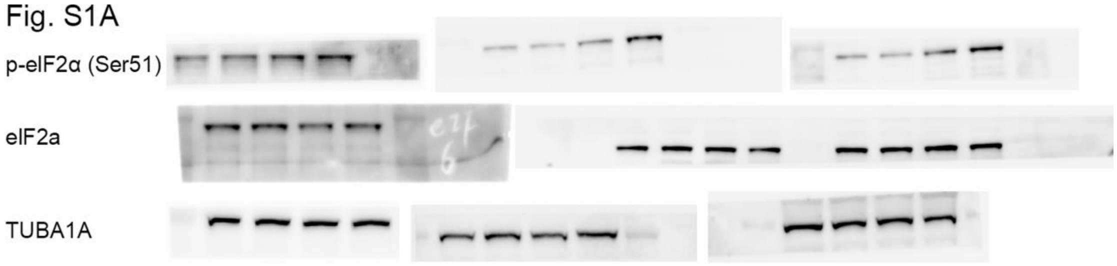

Fig. S3D

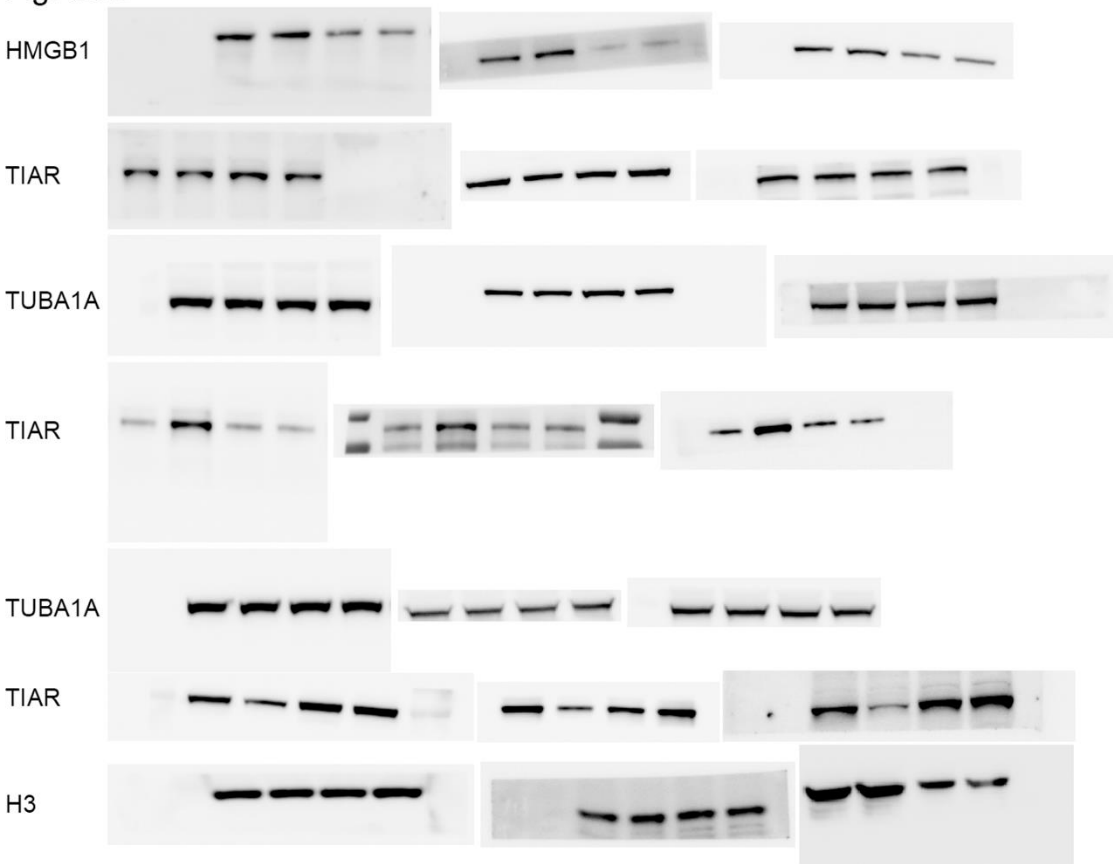

Fig. S4D

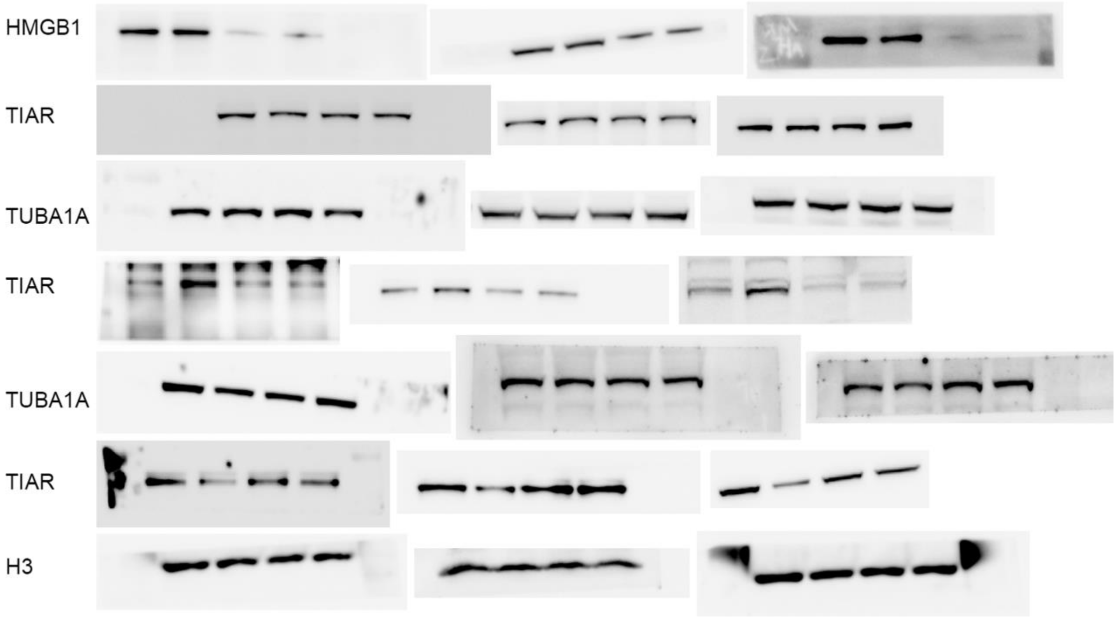

Fig. S5

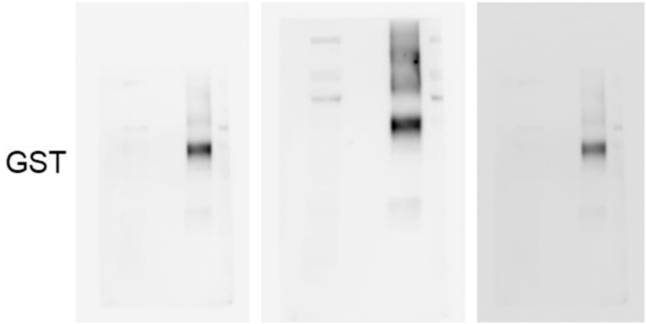

Fig. S6D

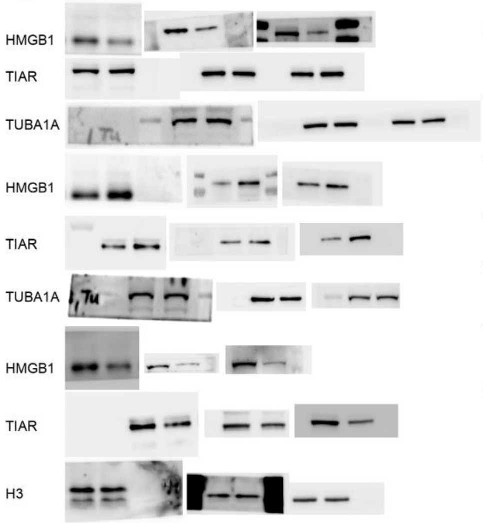

Fig. S6H

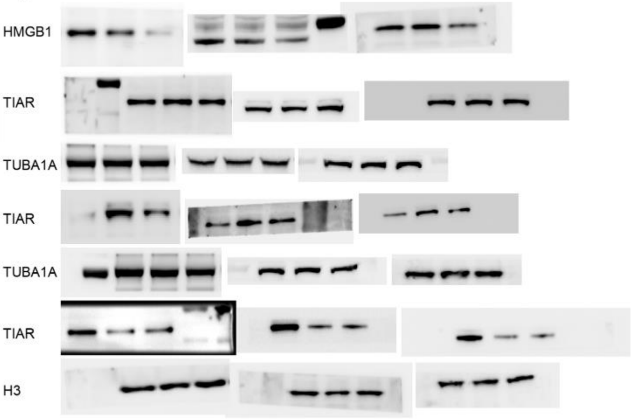

Fig. S6F

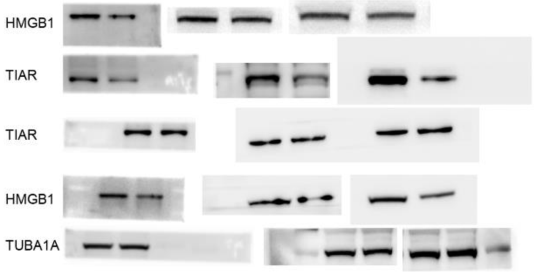

Fig. S7D

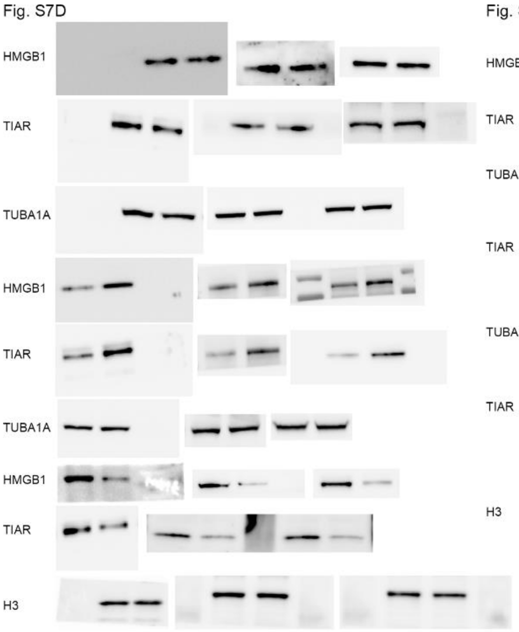

Fig. S7H

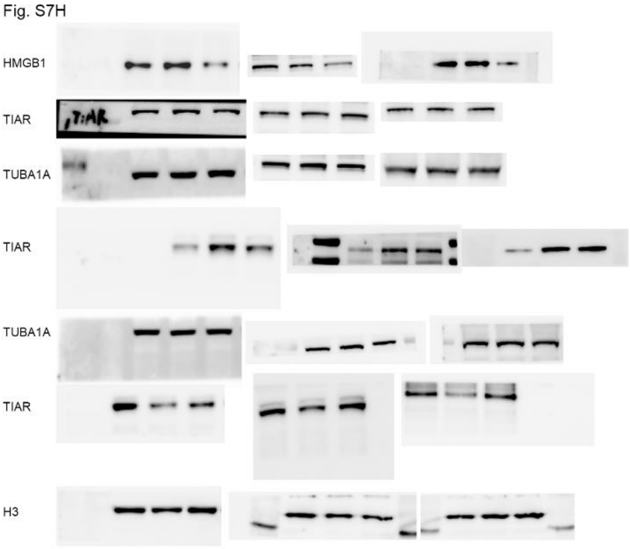

Fig. S7F

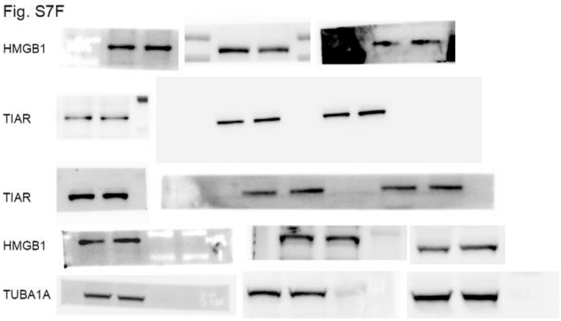

Fig. S9D

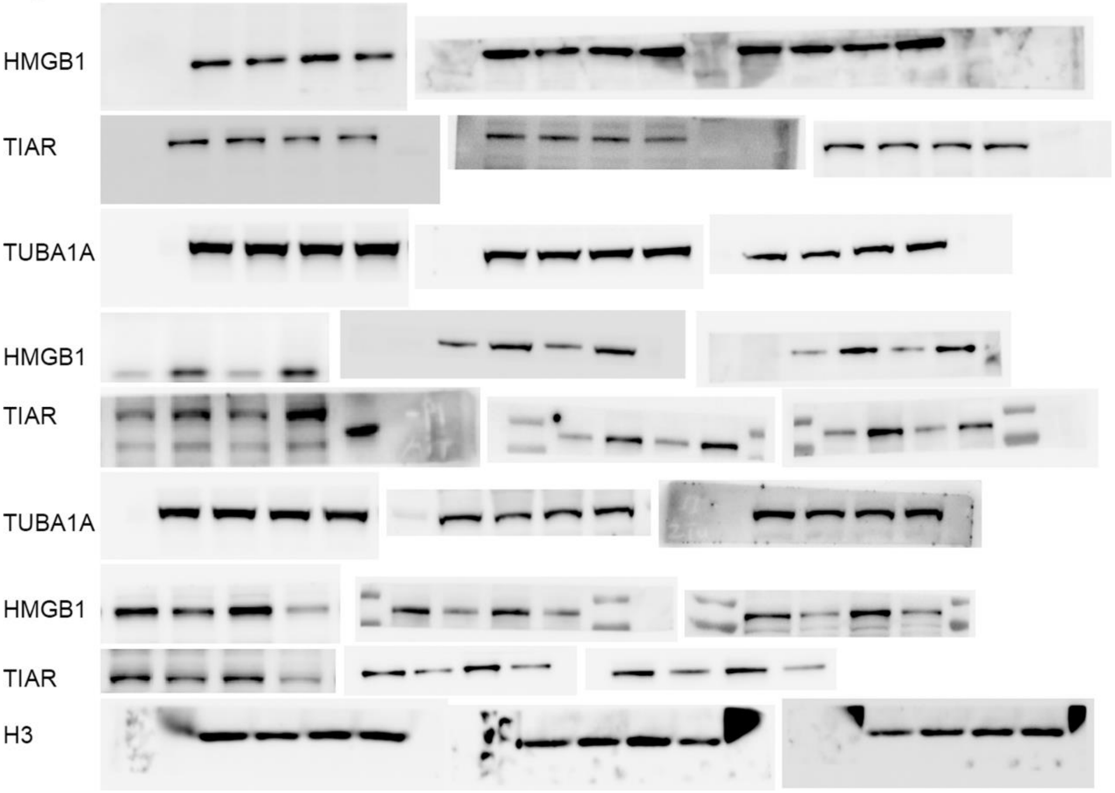

Fig. S10D

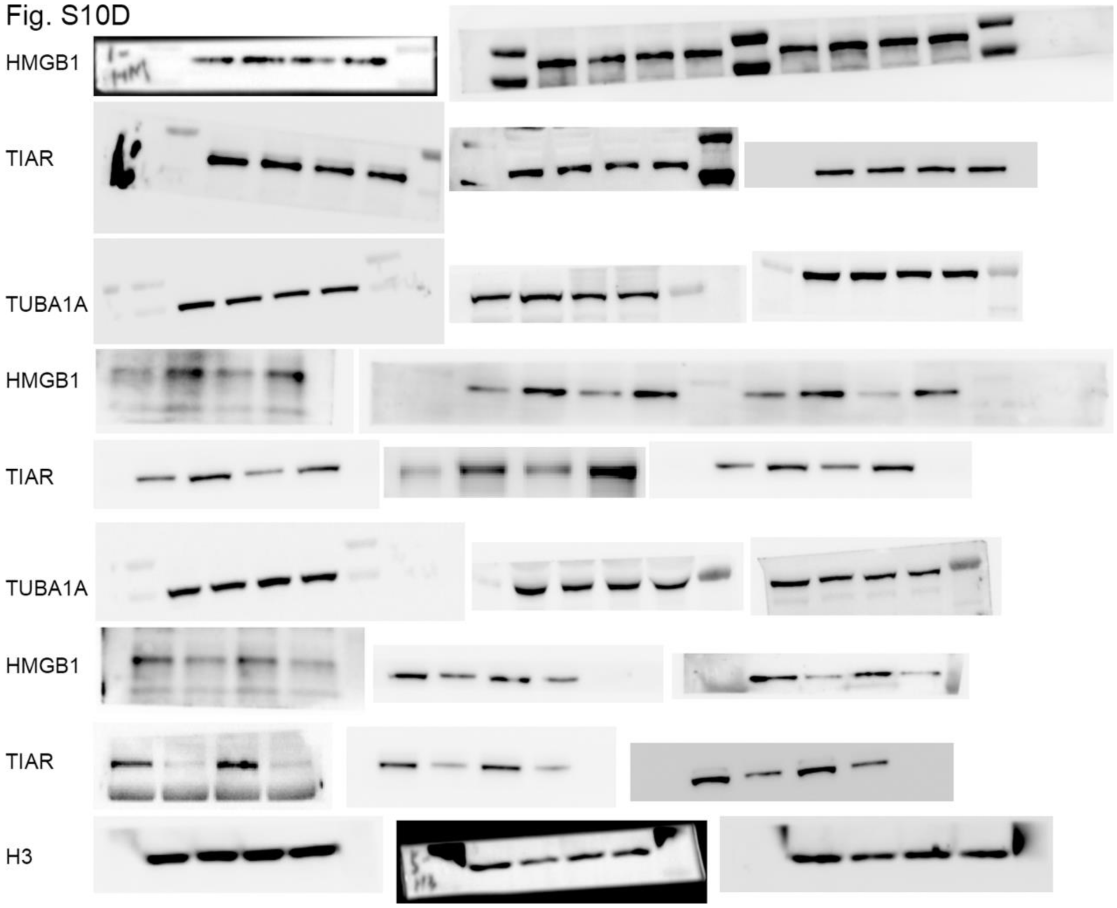

Fig. S11A

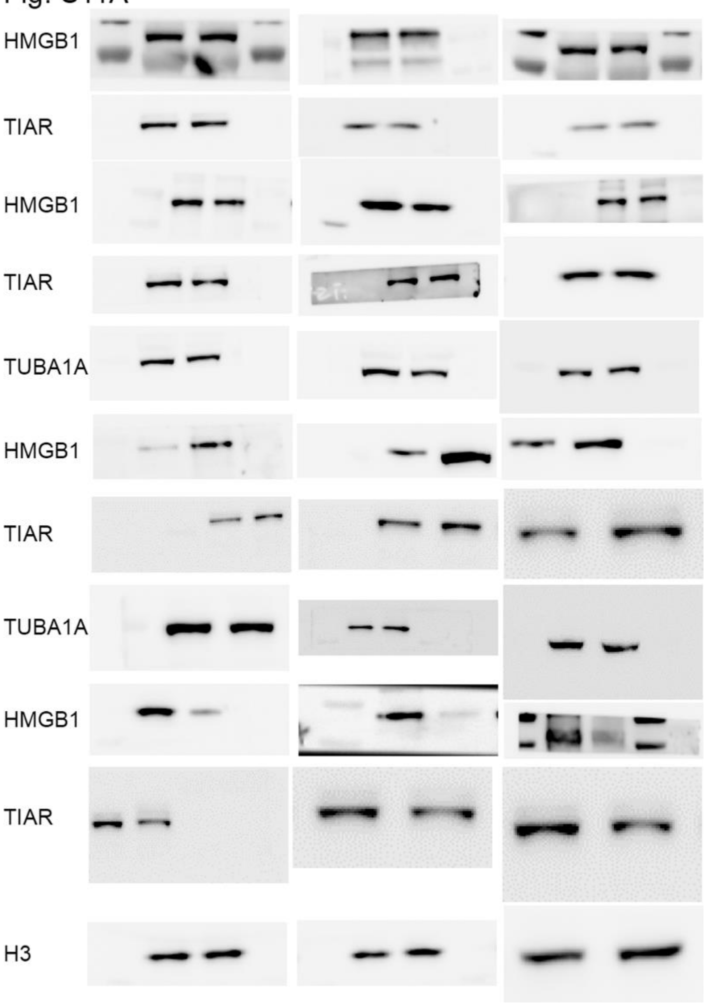
HMGB1
TIAR
HMGB1
TIAR
TUBA1A
HMGB1
TIAR
TUBA1A
HMGB1
TIAR
H3

Fig. S12A

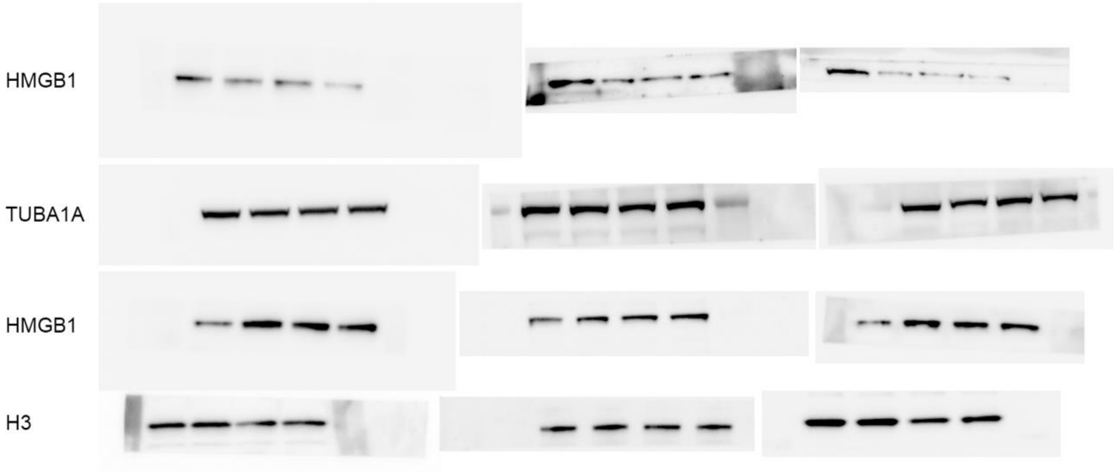

Fig. S12C

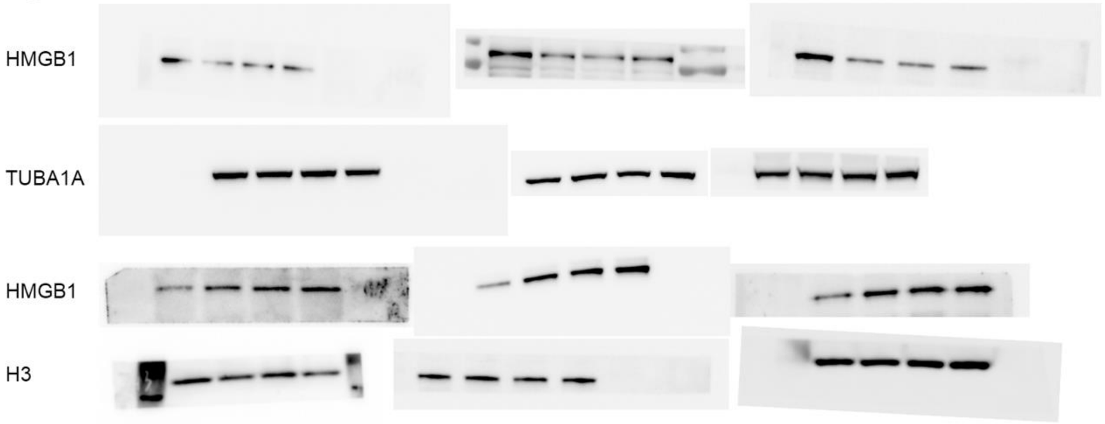

Fig. S14A

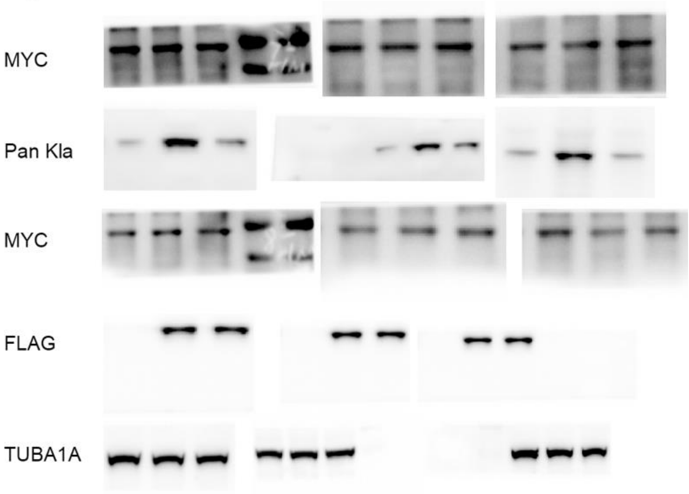

Fig. S15A

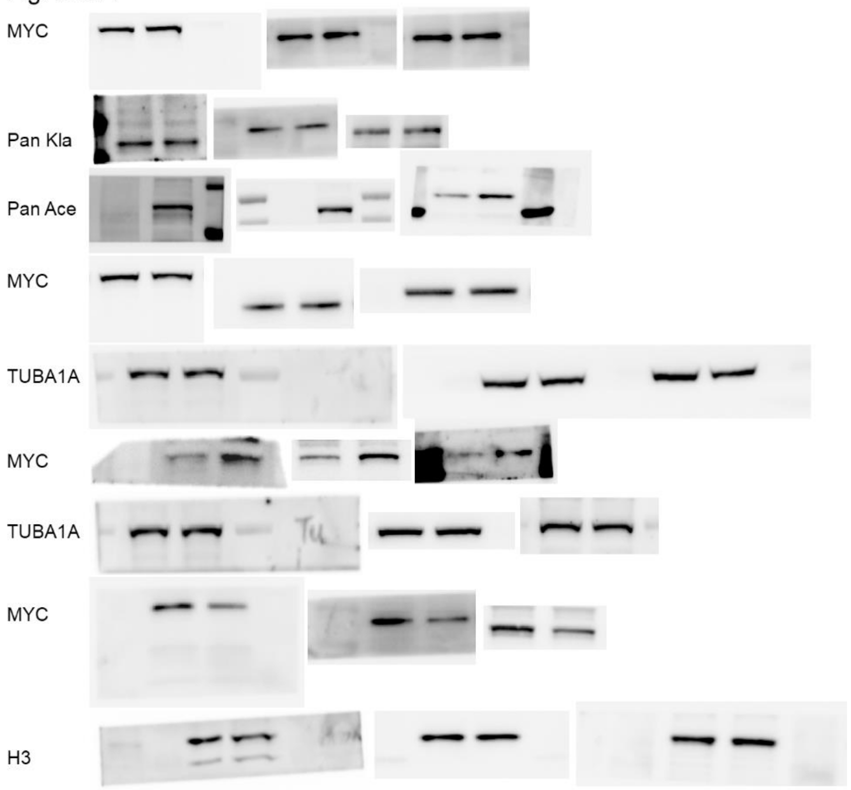

Fig. S16A

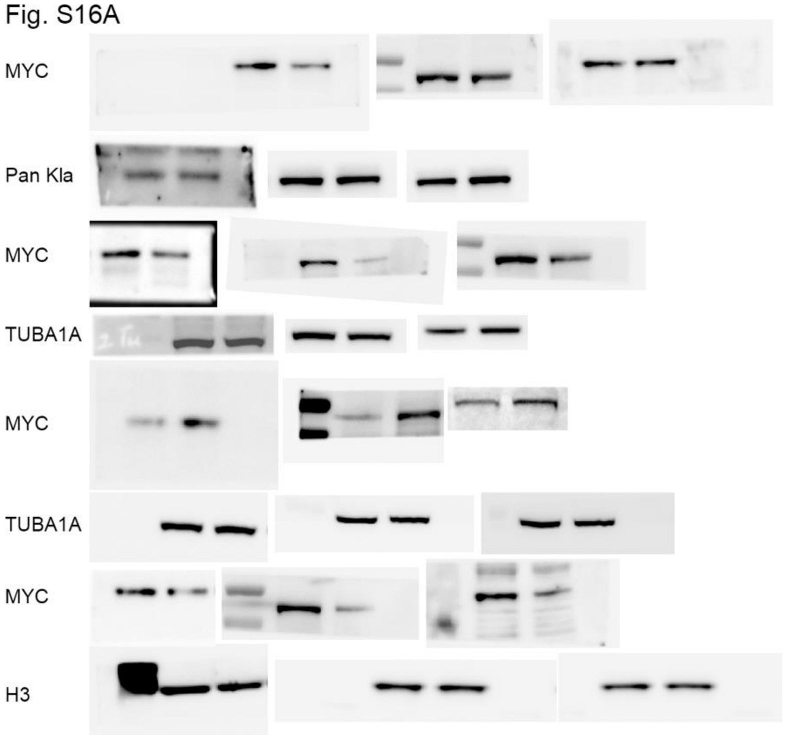

Fig. S17A

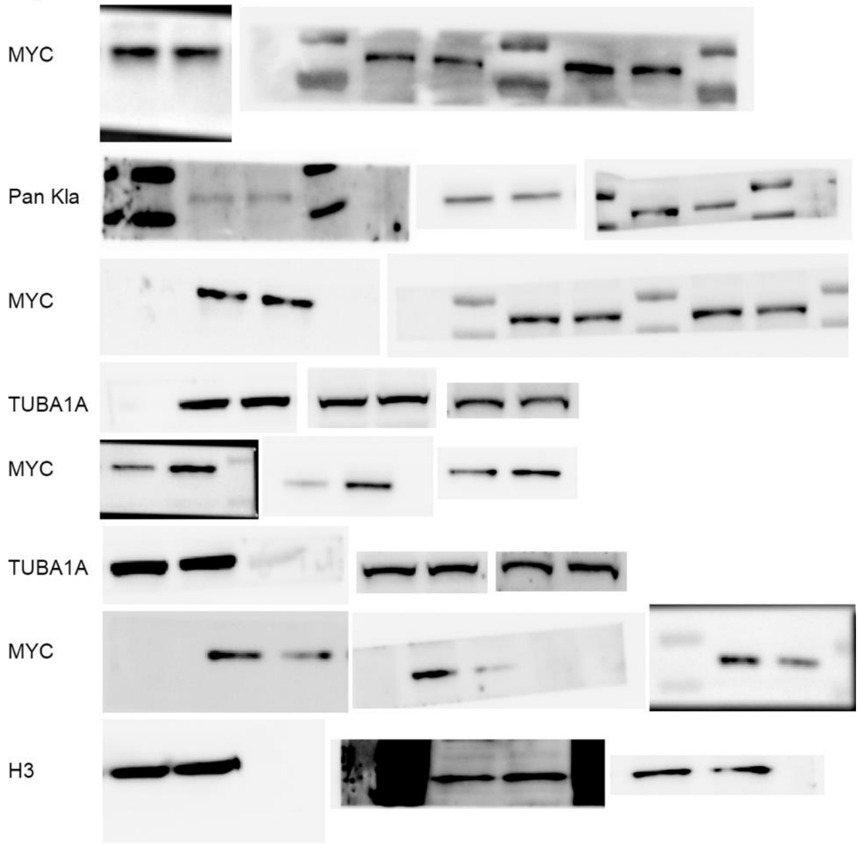

Fig. S18A

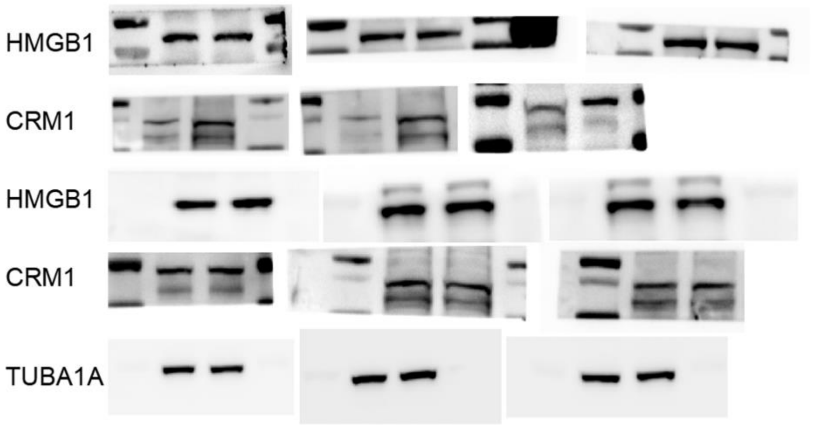

Fig. S18C

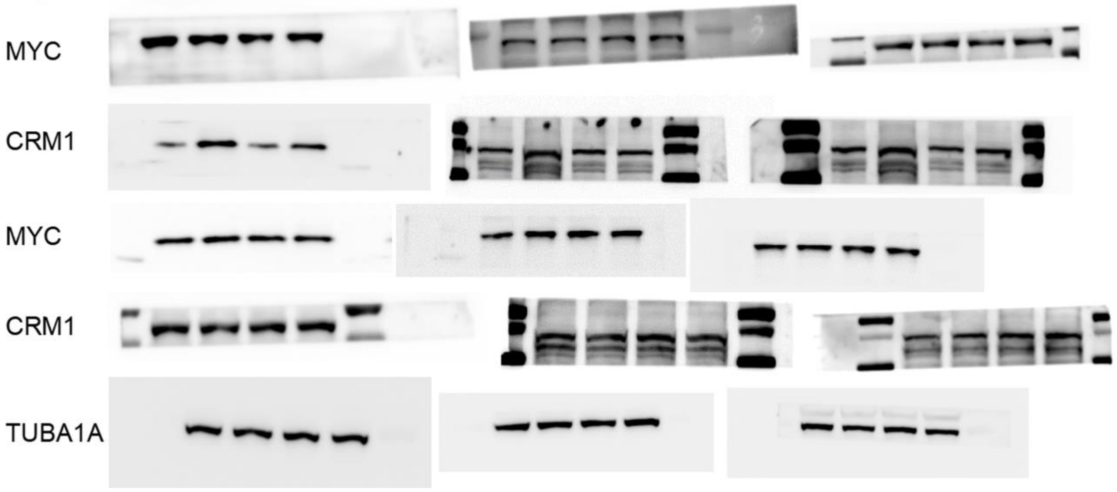

Fig. S18E

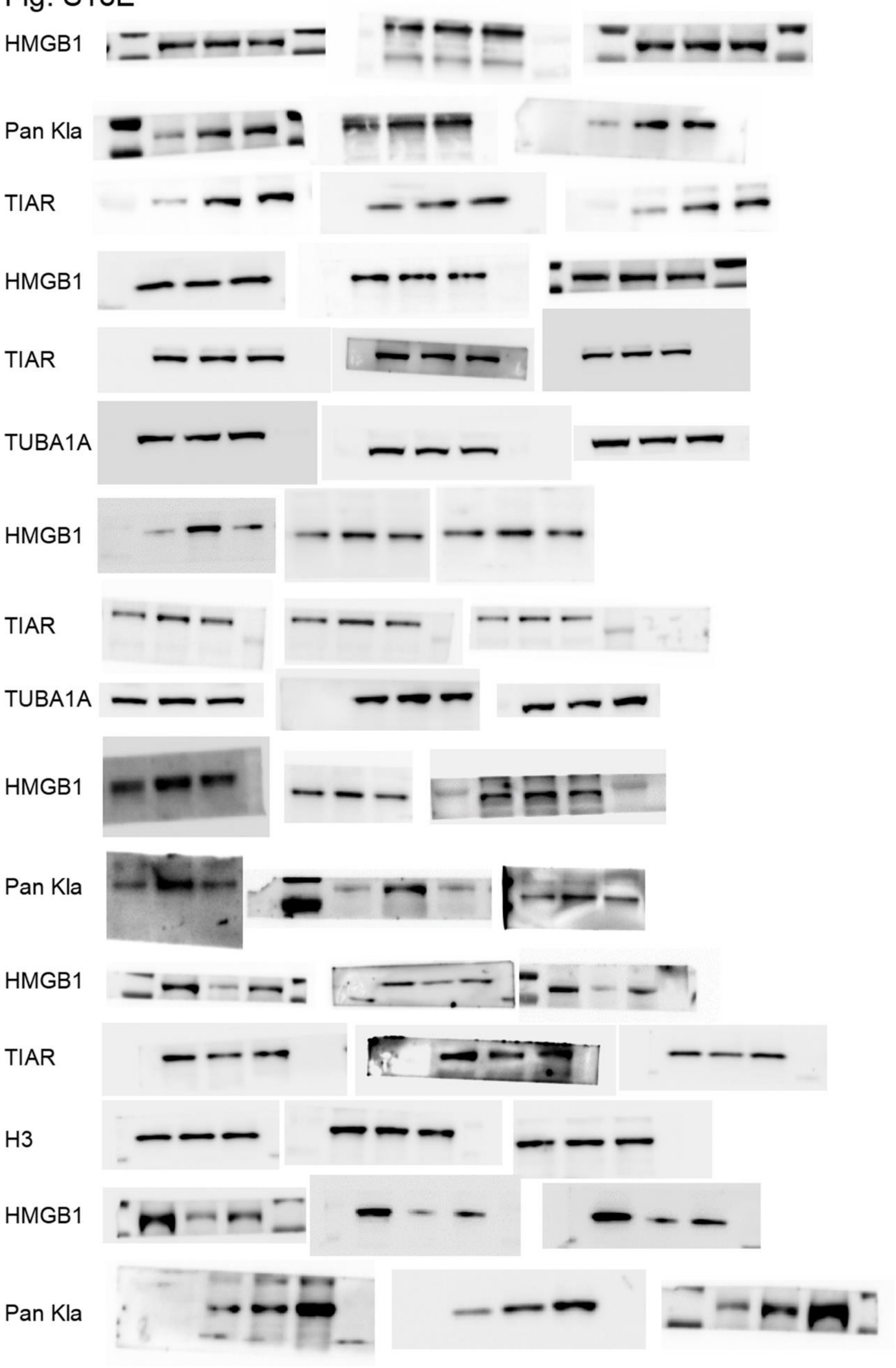
HMGB1
Pan Kla
TIAR
HMGB1
TIAR
TUBA1A
HMGB1
TIAR
TUBA1A
HMGB1
Pan Kla
HMGB1
TIAR
H3
HMGB1
Pan Kla
